# Molecular function recognition by supervised projection pursuit machine learning

**DOI:** 10.1038/s41598-021-83269-y

**Published:** 2021-02-19

**Authors:** Tyler Grear, Chris Avery, John Patterson, Donald J. Jacobs

**Affiliations:** 1 Department of Physics and Optical Science, University of North Carolina at Charlotte, Charlotte, NC 28262 USA; 2 Department of Bioinformatics and Genomics, University of North Carolina at Charlotte, Charlotte, NC 28262 USA; 3 Center for Biomedical Engineering and Science, University of North Carolina at Charlotte, Charlotte, NC 28262 USA

**Keywords:** Biophysics, Computational biology and bioinformatics, Drug discovery, Mathematics and computing

## Abstract

Identifying mechanisms that control molecular function is a significant challenge in pharmaceutical science and molecular engineering. Here, we present a novel projection pursuit recurrent neural network to identify functional mechanisms in the context of iterative supervised machine learning for discovery-based design optimization. Molecular function recognition is achieved by pairing experiments that categorize systems with digital twin molecular dynamics simulations to generate working hypotheses. Feature extraction decomposes emergent properties of a system into a complete set of basis vectors. Feature selection requires signal-to-noise, statistical significance, and clustering quality to concurrently surpass acceptance levels. Formulated as a multivariate description of differences and similarities between systems, the data-driven working hypothesis is refined by analyzing new systems prioritized by a discovery-likelihood. Utility and generality are demonstrated on several benchmarks, including the elucidation of antibiotic resistance in TEM-52 beta-lactamase. The software is freely available, enabling turnkey analysis of massive data streams found in computational biology and material science.

## Introduction

The last two decades have witnessed the widespread use of molecular dynamics (MD) simulations in the fields of material science and biophysics to study molecular systems at an exquisite level of detail^1–4^. When MD simulation data is combined with experimental data, functional mechanisms can be identified which are pivotal for molecular engineering^5–8^ and drug discovery^9–13^. To improve function recognition in molecular engineering^14,15^, challenges in the multivariate analysis of MD simulation data must be overcome^6,16^. As disparate methodologies^10,11,17–20^ are developed, an effective automated process remains in high demand.

Unsupervised machine learning (ML) methods have played an important role in the analysis of MD trajectories. Principal component analysis (PCA) applied to MD data provides dimension reduction (DR) which can characterize the essential dynamics of macromolecules such as proteins^18,21,22^ while reducing the degrees of freedom (df) for the data matrix. PCA identifies large-scale motions that are assumed critical to function; consequently, functional motions will be misidentified as noise if the dynamics have a smaller amplitudinal variance than what is contained in the top PCA modes^23^. Clustering algorithms are often combined with DR and feature extraction techniques such as PCA in order to identify key conformations that facilitate molecular function^24,25^.

Supervised machine learning is capable of identifying functional dynamics by associating experimental and simulation data^26^ in binary classification. To define a labeled training dataset, each system is categorically classified as functional or nonfunctional based on experiment then paired to an MD simulation as a digital twin, a term coined in manufacturing^27^. In practice, supervised ML techniques for discriminant analysis^13,28,29^ such as linear/quadratic discriminant analysis (LDA/QDA), support vector machines (SVMs), and gradient-based neural networks (NNs) perform poorly on a collection of MD data streams. To overcome many severe limitations, we developed a novel projection pursuit framework.

Projection pursuit (PP) is an exploratory statistical technique for projecting high-dimensional data into a lower-dimensional space in order to reveal underlying structure. The PP method consists of two parts: a projection index that quantifies the features of univariate projections, and the optimization of an objective function^30,31^. The projection index orders projections of the data from the most to least relevant for effective dimension reduction. Many variations of projection indices exist; for example, a kurtosis-based index is effective for classification^32^, although noise can be misidentified as a signal^33^. Supervised PP has received recent attention^34,35^, and shown to promote deep learning^36^.

**Figure 1:**
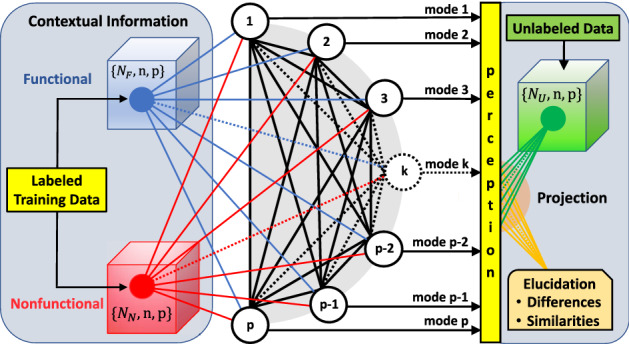
Schematic of SPLOC as a recurrent neural network and data flow. For *p* variables there are *p* perceptrons, labeled from 1 to *p*, comprising the input layer that receives $N_F$ functional and $N_N$ nonfunctional data packets of *n* samples. Each perceptron maps to a mode, and has access to all data packets organized in the form of two types of data packet cubes. Each perceptron recurrently interacts with all other perceptrons through competitive learning. The basis set rotates as the neural network evolves to maximize efficacy. Upon convergence, all perceptrons comprise the output layer for the specification of an orthonormal complete basis set. A rectifying function is assigned to each perceptron, defining a viewpoint for controlling sensitivity and selectivity in feature extraction. For a given viewpoint, the final basis set defines perception when the neural network achieves maximum efficacy. Unlabeled data packets are subsequently classified within the context of training data, having multivariate discriminant and conserved features that are readily interpretable.

Supervised Projective Learning with Orthogonal Completeness (SPLOC) was initially developed for molecular function recognition as a PP optimized by an NN. However, SPLOC transcends applications as a general purpose recurrent NN (RNN). The SPLOC-RNN depicted in Fig. 1 provides discriminant analysis and creates perception. Details follow in Methods and in the Supplemental Information.

## Overview of SPLOC-RNN

**Figure 2:**
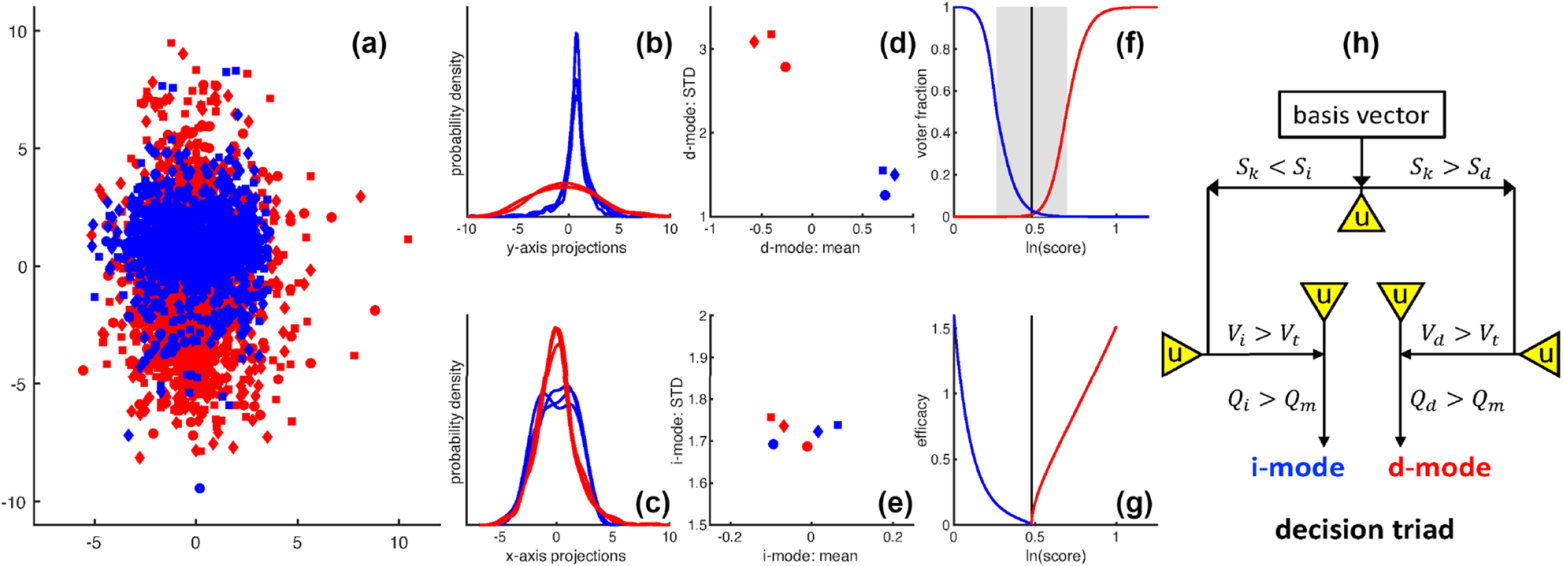
Key aspects of SPLOC-RNN. **(a)** Example scattered data in a plane for six data streams. **(b)** Along the y-axis, data packets separate into two distinct classes. **(c)** Along the x-axis, all six data packets share similar emergent properties. These observations follow from the probability densities for scattered data projections. **(d)** A mode feature space plane (MFSP) plots the mean and standard deviation (STD) of the probability density along the y-axis for a discriminant-mode (d-mode). **(e)** The MFSP is shown for the probability density along the x-axis for an indifferent-mode (i-mode). **(f)** Voting activation functions for an i-mode (blue) and d-mode (red) are shown. The re-entrant behavior at the 50% levels bound the indecision region (gray area) due to uncertainty in discerning differences or similarities between systems. **(g)** The rectifying adaptive nonlinear unit is shown when d-mode (red) and i-mode (blue) functions have MFSP clustering quality factors set to unity. The bifurcation line is shown as a vertical black line in panels **(f,g)**. **(h)** Flow chart for mode selection. The decision triad stratifies the complete set of basis vectors into discriminant, undetermined and indifferent modes. Discriminant and indifferent modes respectively quantify differences and similarities in features when signal-to-noise, statistical significance and quality of clustering all surpass minimum thresholds, otherwise the projection is an undetermined-mode, denoted by yellow triangles.

The PP-based NN was shown to be effective^37,38^ in the 1990s. Here, several ML strategies are integrated with PP operating on data packets. Advantages for using data packets are illustrated in Fig. 2a–e for overlapping data streams. The mean and standard deviation (STD) of a data stream projected onto a basis vector (a mode direction) quantifies differences and similarities. These emergent properties are visualized in a mode feature space plane (MFSP), representing a two-dimensional (2D) cross-section in high dimensions.

The RNN setup maps each mode to a perceptron, each with access to two distinct classes of data packet cubes^39^. Each perceptron has a rectifying unit to quantify mode efficacy as a function of signal-to-noise and clustering quality within the MFSP. Signal-to-noise is used to rank order the modes, and bifurcate emergent properties into discriminant and indifferent characteristics. Statistical significance is evaluated using voting activation functions shown in Fig. 2f.

A *rectifying adaptive nonlinear unit* (RANU), shown in Fig. 2g, controls feature extraction. Perceptron pairs undergo competitive learning to maximize efficacy of the perceptron network using directed orthogonal rotations with data-driven importance sampling. The decision tree shown in Fig. 2h selects discriminant, undetermined, and indifferent modes; respectively denoted as d-modes, u-modes and i-modes. The discriminant and indifferent subspaces respectively explain differences and similarities between systems. Despite low information content in the undetermined subspace, randomized orthogonal rotations on u-modes induce creativity in discovery as latent information is extracted. The psuedocode for SPLOC is given in Algorithm 1. The algorithm fits the general pattern of PP, however, PP is being used to maximize efficacy over a network of perceptrons in a recurrent fashion.
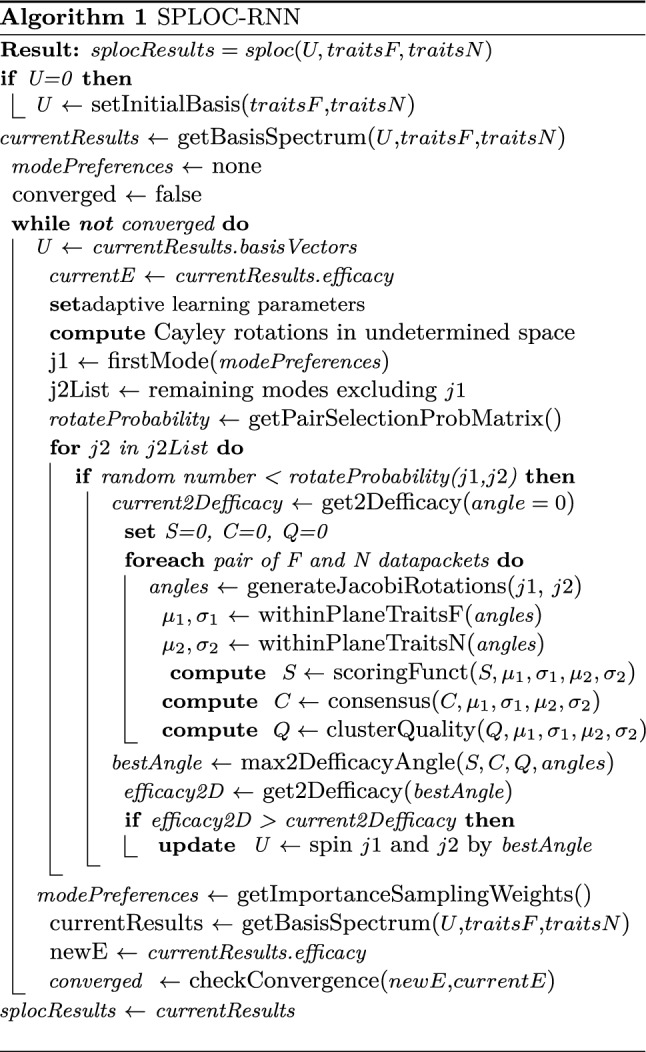


Without required preprocessing of input data and void of hyperparameters, SPLOC-RNN performs derivative-free optimization within a nonparametric model on high dimensional data without limit on sample size. Furthermore, mitigation of overfitting to training data is an automated process that improves with greater observations per variable (OPV). For efficient hypothesis refinement, a discovery-likelihood (DL) is introduced using Bayesian inference for candidate ranking.

## Results and discussion

### Iris and wine dataset benchmark

The Iris^40,41^ and wine^42^ datasets each have three classes of data containing $p=4$ and $p=13$ variables respectively. Bootstrapping was employed to create data packets comprised of 10 and 15 samples for Iris and wine datasets, yielding an OPV of 2.5 and 1.15 respectively. Only part of the labeled data was used for training. For Iris and wine datasets, 4 and 11 d-modes were extracted respectively. Similar results are obtained when correlation matrices replace covariance matrices (results not shown); subsequently, only 3 and 7 d-modes are extracted for Iris and wine datasets respectively. The reduction of d-modes using correlation matrices reflects a loss of information due to normalization.

**Figure 3:**
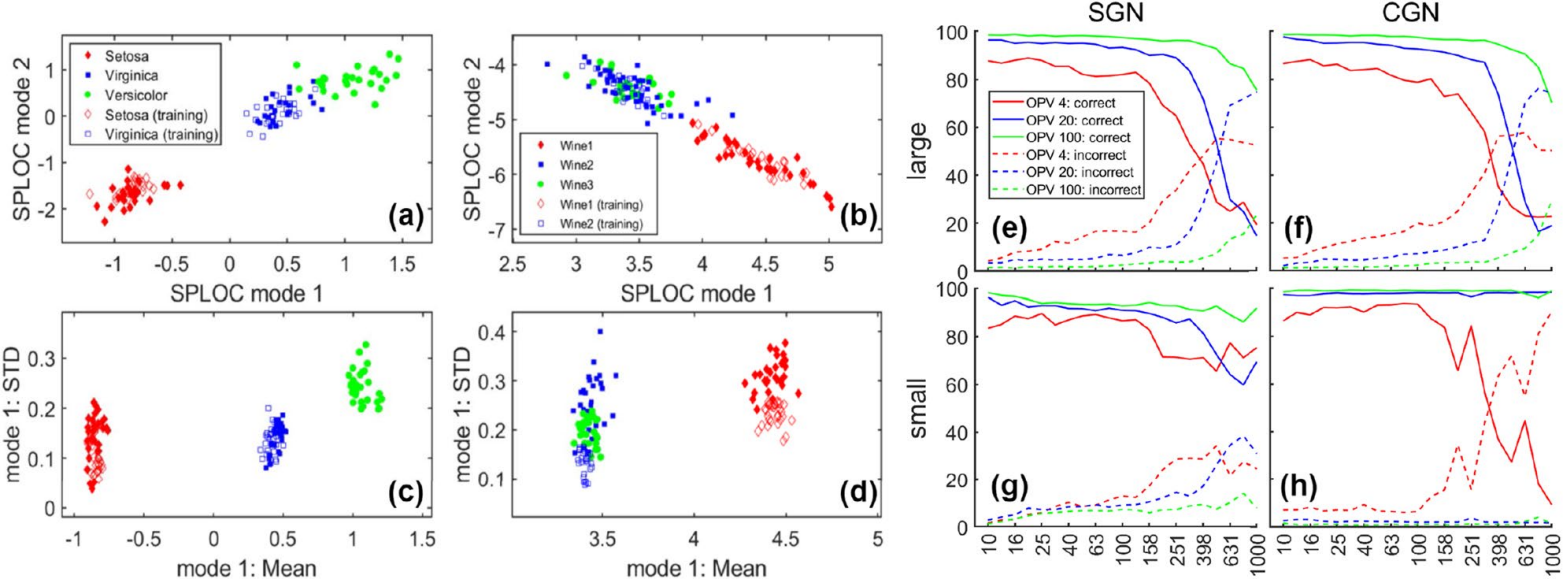
Classification and feature extraction benchmarks. The top two d-modes project the raw data into two dimensions for the **(a)** Iris and **(b)** wine datasets. The mean and STD of data packets are shown within a mode feature space plane for the top d-mode that describes the **(c)** Iris and **(d)** wine datasets. In the right panel, four tabulated graphs show egg reconstruction percentages in the discriminant (correct) and indifferent (incorrect) subspaces as a function of system size for 4, 20 and 100 observations per variable (OPV). System size is characterized by the number of df. **(e–h)** Four tabulated plots are shown for a (large, small) egg placed in (SGN, CGN). Egg reconstruction percentages are shown for discriminant (correct) and indifferent (incorrect) subspaces as a function of df at an OPV of 4, 20 and 100.

The raw data was first projected into the top two d-modes. For Iris data, Fig. 3a shows perfect class separation is achieved between Setosa and Virginica, with unlabeled Versicolor being more like Virginica. For wine data, Fig. 3b shows class separation is nearly achieved between wine 1 and wine 2. Furthermore, wine 3 is indistinguishable to wine 2. A prudent approach for discriminant analysis is to work with the emergent MFSP associated with each d-mode separately.

For the top d-mode, Fig. 3c shows perfect class separation across all species in the Iris data, and Fig. 3d exhibits perfect class separation between wine 1 and wine 2. Wine 3 shares wine 2 features captured by the first d-mode. Scatter plots within an MFSP for all d-modes with Iris and wine datasets are shown in Supplementary Figures S1 through S3. Emergent properties from unseen data packets may be similar or different to that of the training data packets on a per mode basis.

### Egg hunt benchmark

The ability of SPLOC to detect a latent signal embedded within a noisy environment and its susceptibility to misidentifying noise as a signal are assessed systematically using four models. A noisy environment in *p* dimensions was created using two multivariate Gaussian distributions. First, structureless Gaussian noise (SGN) models each random variable as independent and normally distributed. Second, correlated Gaussian noise (CGN) was generated using a covariance matrix that has an eigenvalue spectrum shown in Supplementary Figure S4, which qualitatively mimics a typical PCA spectrum for the essential dynamics of globular proteins.

Nonfunctional systems modeled as SGN or CGN provide a concealing environment. A perturbation was applied to place a 2D “egg” in the system. Thereafter, the egg was scrambled within a 6-dimensional subspace. This yields SGN-egg and CGN-egg representing functional systems that have the same statistical properties as their SGN and CGN counterparts, except for 2 out of *p* dimensions where the 2D egg is laid. Large and small egg characteristics are visualized in Supplementary Figures S5 through S8.

An egg hunt means training SPLOC on (SGN-egg)-SGN or (CGN-egg)-CGN as two examples of a function-nonfunction pair. After training, the sets of (d-modes, u-modes, i-modes) are used to calculate the percent of the egg reconstructed in the (discriminant, undetermined, indifferent) subspaces denoted as ($X_d, X_u, X_i$), where $X_d + X_u + X_i = 100$. Note that $X_d$ and $X_i$ respectively reflect true-positive and false-negative predictions, while $X_u$ is noncommittal error. For perfect DR: $X_d = 100$ and $X_u = X_i = 0$ using 2 d-modes and $p-2$ i-modes. An egg hunt was also performed on SGN-SGN and CGN-CGN as a control, where *p* i-modes should be extracted.

A dozen egg hunts were performed with large and small eggs placed in SGN and CGN concealing environments for 4, 20 and 100 OPV. Typical results are shown in Supplementary Figure S9. Figure 3e–h summarizes average egg reconstruction percentages from d-modes and i-modes over 10 trials per system size ranging from 10 to 1000 df. At $\approx$ 200 df the onset of a sharp drop in DR accuracy occurs for large eggs. For small eggs, DR accuracy gradually drops in SGN; and for CGN, high DR accuracy is maintained at an OPV of 20 or more.

**Figure 4:**
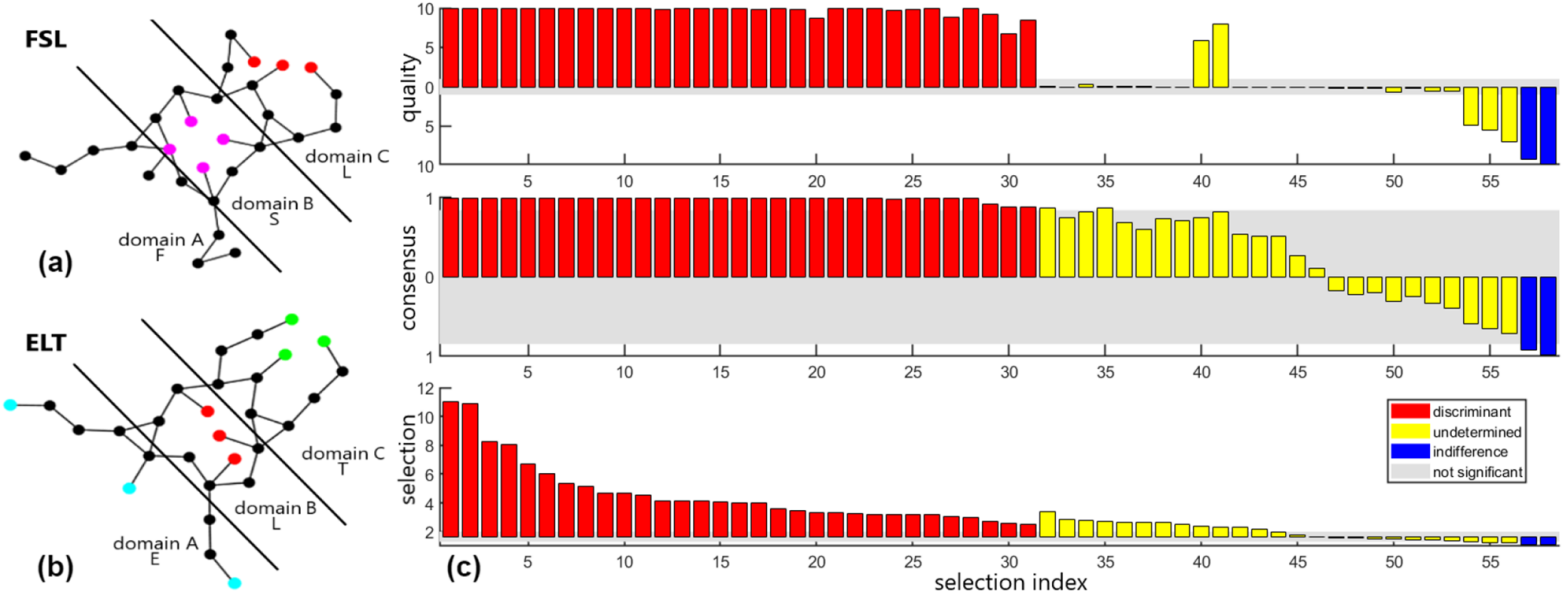
Discriminant analysis on synthetic molecules. **(a)** The synthetic molecule FSL indicates Free-Square-Linear geometric signatures are present within domains A, B, and C respectively. Free indicates no restraining forces are applied in that region. **(b)** The synthetic molecule ELT indicates Extended-Linear-Triangular geometrical signatures within domains A, B, and C respectively. The extended structure emulates a pose for binding to a receptor. **(c)** The vector space is partitioned into (discriminant, undetermined, indifferent) subspaces, spanned by (d-modes, u-modes, i-modes) shown in bar-graphs, colored as (red, yellow, blue). The decision triad criteria are shown as stacked bar graphs with selection (bottom) corresponding to the signal-to-noise scoring function that rank-orders the modes within each subspace separately, consensus (middle) and quality (top). Consensus and quality values are plotted (above, below) the 0-reference line when selection is (above, below) the bifurcation reference. Gray background shows the decision triad acceptance thresholds. As an output from SPLOC-RNN, the stacked bar graph format highlights the characteristic strengths and weaknesses of each mode.

The average number of extracted d-modes and u-modes are shown in Supplementary Figures S10 and S11 for twelve egg hunts and the control. At 4 OPV and beyond 200 df, a rapid increase in the number of extracted u-modes occurs as *p* increases. Generally, accuracy increases as OPV increases, and decreases as *p* increases. On average, training time increases as OPV decreases because greater statistical fluctuations create more u-modes and uncertainty. In the absence of an egg, only i-modes were obtained for 20 and 100 OPV, with mild false egg detection at 4 OPV. The egg hunt allows time complexity to be benchmarked. As shown in Supplementary Figure S12, CPU-time is sub-quadratic in *p* up to 1000 df, despite the worse case time complexity of $$p^4 N_F N_N $$.

Marked performance gain is obtained when the vast majority of modes are u-modes because the algorithm preferentially spins d-modes and i-modes against u-modes, saving on u-u mode pairs. This bias rapidly generates new d-modes or i-modes when they exist. As more d-modes and i-modes are extracted, more mode pairs require checking. In absence of extracting new d-modes or i-modes, the efficacy of the network converges before most mode pairs are checked. In another scenario, CPU-time is greatly reduced when the initial conditions using PCA identify most i-modes and d-modes in advance of spinning mode pairs. In general, sub-quadratic dependence on *p* will occur with sparse numbers of d-modes and i-modes because convergence sets in rapidly at the wings of the spectrum where the small numbers of d-modes and i-modes reside.

Generating and indexing basis vectors are separate steps. Egg hunts employ PCA to create an initial basis set (IBS) which is indexed by the decision triad, setting a baseline efficacy. During training, efficacy increases. This gain, shown in Supplementary Figure S13, suggests the final basis set (FBS) will exhibit better egg reconstruction. Egg reconstruction percentages, along with number of d-modes and u-modes are plotted in Supplementary Figures S14 through 16 for the IBS. Better egg reconstruction is achieved using the (IBS, FBS) for (large, small) eggs. This dichotomy occurs when u-modes in the IBS are replaced by i-modes at the expense of losing d-modes. Greater portions of a localized signal are reconstructed from i-modes as *p* increases. This indicates the RANU biases predictions toward low sensitivity and high selectivity for large systems.

Extracting similarities and differences between classes creates a data-driven hypothesis for the most relevant factors that elucidate differences. PCA has the fixed hypothesis that variance explains differences. The IBS obtains excellent DR for a large egg because the PCA hypothesis is true. The IBS poorly reconstructs a small egg in CGN because as *p* increases, the variance in the top PCA-modes are dominated by the properties of the environment, thereby losing sight of the egg. These egg hunt results demonstrate SPLOC-RNN is reserved in extracting differences between systems.

### Molecular function recognition

The process of classifying 24 synthetic molecules was assessed to illustrate the feasibility of molecular function recognition. Each synthetic molecule has 29 atoms that are constrained to the xy-plane; thus, $p=58$ df. Trajectories of 500 and 20,000 frames are analyzed as two separate cases. All conformations are structurally aligned to one reference structure. Restraint forces are added between atoms to create correlated motions that emerge as geometrical signatures. The nomenclature for each molecule specifies the geometrical signatures within three structural domains. The available signatures for each domain are: Domain A: F (free), E (extended); Domain B: F (free), L (linear), S (square), T (triangular); Domain C: F (free), L (linear), T (triangular). The allowed permutations are denoted as abc where a total of 24 distinct synthetic molecules are possible. Examples of FSL and ELT are depicted in Figures 4a and 4b respectively.

#### Dimension reduction

The DR component of SPLOC (DR SPLOC) was assessed by applying standard ML binary classification methods on subsets of d-modes. For this analysis, all aLc molecules are functional. The training set consists of {FLL, FLF} as known functional systems, and {FFL, FFF} as known nonfunctional systems. To establish a benchmark, PCA and partial least squares (PLS) are employed as alternative DR methods. Each DR method presents different features to the classifiers. The quality of DR from PCA, PLS and DR SPLOC for (3, 8, 13) dimensions is compared based on how well the 20 unlabeled molecules are classified. Figure 4c shows the SPLOC-mode spectrum, yielding 31 d-modes, 25 u-modes and 2 i-modes.

Projecting trajectories into the PCA, PLS, and DR SPLOC subspaces of DIM (3, 8, 13) results in data matrices of size ($3 \times n$, $8 \times n$, $13 \times n$) for each synthetic molecule where *n* is 500 or 20,000 samples. At each DIM, six binary classification methods are applied: LDA; QDA; naive Bayes with a Gaussian kernel (GNB); support vector machines with linear kernel (LSVM), quadratic kernel (QSVM), and radial basis kernel (RBSVM). All methods were benchmarked using 4-fold cross-validation, the results are shown in Supplementary Table S1.

For all synthetic molecules the likelihood to be functional for each method, including the DL, is summarized in Supplementary Tables S2 through S7. The tabulated results reveal several quantitative trends. There is no classification method that singles out as being the worst or best. Clear trends are revealed by taking an average over all six classification methods. On average, for all DIM (3, 8, 11), PLS has (poor, good) DR quality at (500, 20,000) samples, whereas PCA has poor DR quality in all cases, except for DIM 13 at 20,000 samples where it has marginally good DR quality. DR SPLOC across all classification methods yields better DR quality, ranging from (good, excellent) at (500, 20,000) samples. Generally, DR quality improves for PCA and PLS as DIM increases, but is insensitive to DIM for DR SPLOC.

These trends become apparent when evaluating classifier performance with Cohen’s kappa statistic^43^ summarized in Table 1 using a threshold of 1/2. Similar results are obtained with thresholds between 1/5 and 4/5. Cohen’s kappa statistic overcomes accuracy bias with regards to imbalanced data. Given 6/24 synthetic molecules are functional and 18/24 are nonfunctional, this metric provides a quantitative evaluation of how each of the six standard classifiers perform within the subspaces spanned by PCA, PLS, and DR SPLOC. At DIM 3 and 500 samples, classifer reliability when using PCA and PLS for DR are in more agreement with random guessing than the ground truth. At DIM 8 and 13, the gap in performance for PLS and PCA begins to close. At 20k samples and DIM 3, it becomes clear that DR using PCA does not capture discriminating characteristics. These results show that DR SPLOC provides a more generalized model, having the best overall DR characteristics.

**Table 1:** Cohen’s kappa statistic for intra-rater reliability against ground truth at 500 and 20k samples.

		**PCA**	**PLS**	**DR SPLOC**
**500 samples—Cohen’s kappa**				
**3 modes**	max	0.200	0.385	0.882
	avg.	0.181	0.247	0.835
	min	0.161	0.125	0.600
**8 modes**	max	0.600	0.600	.882
	avg.	0.394	0.285	0.774
	min	0.143	0.125	0.600
**13 modes**	max	0.600	0.882	0.882
	avg.	0.371	0.395	0.645
	min	0.231	0.125	0.333
**20k samples—Cohen’s kappa**				
**3 modes**	max	0.143	0.565	1.000
	avg.	0.060	0.345	0.9825
	min	0.000	0.125	0.895
**8 modes**	max	0.714	1.000	1.000
	avg.	0.549	0.806	1.000
	min	0.385	0.120	1.000
**13 modes**	max	0.714	1.000	1.000
	avg.	0.602	0.822	1.000
	min	0.565	0.217	1.000

Evaluated over the same scenarios, DL yields assiduous predictions when observed characteristics are foreign. This is illustrated in two cases. First, DL generally cannot correctly classify unlabeled molecules as functional or nonfunctional in the PCA or PLS subspaces, with performance worsening as sample size increases. This is because in general no clustering occurs in the emergent MFSP from PCA and PLS modes. Shown in Supplementary Figure S17, the PCA mode projections for functional and nonfunctional molecules are essentially the same. Consequently, more sampling exacerbates a wrong hypothesis noticeably. Second, for DR SPLOC, DL classification is adequate for 500 samples and virtually exact for 20,000 samples, except for molecules of the form aSc. Since aSc does not share similar functional or nonfunctional characteristics with aLc, as exemplified in Supplementary Figure S18, an experimental test on aSc for function will likely discover new knowledge.

#### Iterative learning

A function recognition pipeline (FRP) is illustrated that alternates experiments with ML predictions on digital twins created by MD simulations. Two synthetic molecules, labeled as functional (F) and nonfunctional (N), define an initial training set. The DL ranks all unlabeled digital twins. Verification was then performed on the top candidate to expand the training set. When the prediction is (true, false), the molecule is labeled as (F, N). Bootstrapping is used to create three data packets per molecule in a training set. This iterative procedure is performed 14 times, amounting to 16 “experiments” including the initial two molecules.

Consider 6 synthetic molecules of the form aLc as functional, and the remaining 18 molecules nonfunctional. Selecting 1 F molecule and 1 N molecule leads to 108 initial training sets (e.g. $6 \times 18$) that launch the FRP. Each of the 108 FRP scenarios are simulated 3 times for a total of 324 trials. In turn, 6 synthetic molecules of the form aLc, aSc, aTc and aFc are considered functional to obtain results on four cases. In addition, sample sizes of 500 and 20,000 are considered, corresponding to an OPV of 8.6 and 344.8 respectively.

**Figure 5:**
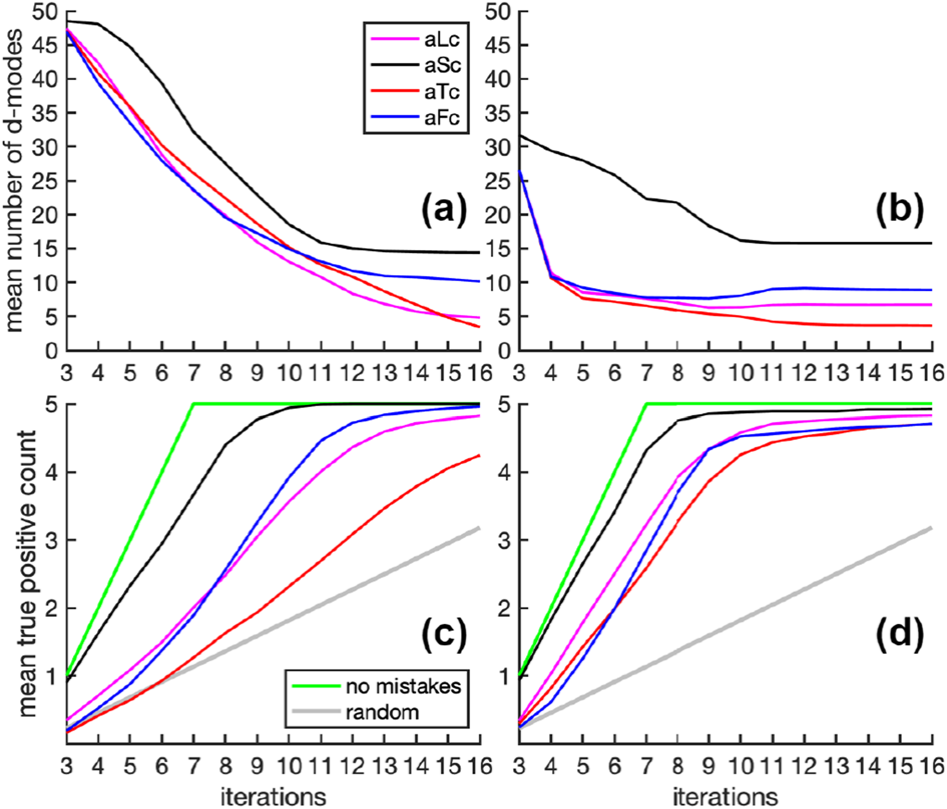
Characteristics of refining the working hypothesis by iterative learning. Mean number of d-modes versus iteration for **(a)** 500 samples and **(b)** 20,000 samples. Mean number of true positives for **(c)** 500 samples and **(d)** 20,000 samples. The result from random guessing among all remaining unlabeled molecules per iteration is shown by the gray line. The green line indicates mistake-free predictions.

Figure 5a,b shows the dimension of the discriminant subspace decreases as more training data becomes available. This shrinking occurs because the differences found between functional and nonfunctional molecules in the training dataset are not all critical for function. As more functional and nonfunctional examples are included in the training set, the working hypothesis for functional dynamics narrows. The path taken to arrive at a relevant working hypothesis depends on how learning responds to mistakes. Upon a classification error, the model is retrained with all current labeled data.

Figure 5c,d shows the true-positive discovery rate in function recognition. An increase in accuracy occurs with greater sampling because confounding physical origins with statistical fluctuations in hypothesis development is reduced. Since each molecule has distinct dynamics to some degree, even in the absence of random noise, functional dynamics are deduced by learning from mistakes. The aTc molecules have the slowest iterative learning rate because they are highly flexible (data not shown). To hone in on differences between flexible molecules requires more trial and error. The aTc molecules are particularly challenging to classify because they are not maximally or minimally flexible.

### Discriminant analysis on beta-lactamase

Many harmful bacteria secrete the enzyme beta-lactamase which provides resistance to penicillin and cephalosporin antibiotics. The TEM family of beta-lactamase contains many isoforms with varying substrate specificity^44^. The hypersensitivity of beta-lactamase to mutations creates an effective means for bacteria to survive against new antibiotics. A major medical problem is extended spectrum resistance (ESR), when beta-lactamase permissively binds to many antibiotics. Experiments show that TEM-1 and TEM-2 resist specific antibiotics, while TEM-52 exhibits ESR. With respect to TEM-1, TEM-2 differs by one point mutation (Q39K)^45^ and TEM-52 differs by three (E104K, M182T, G238S)^46^.

**Figure 6:**
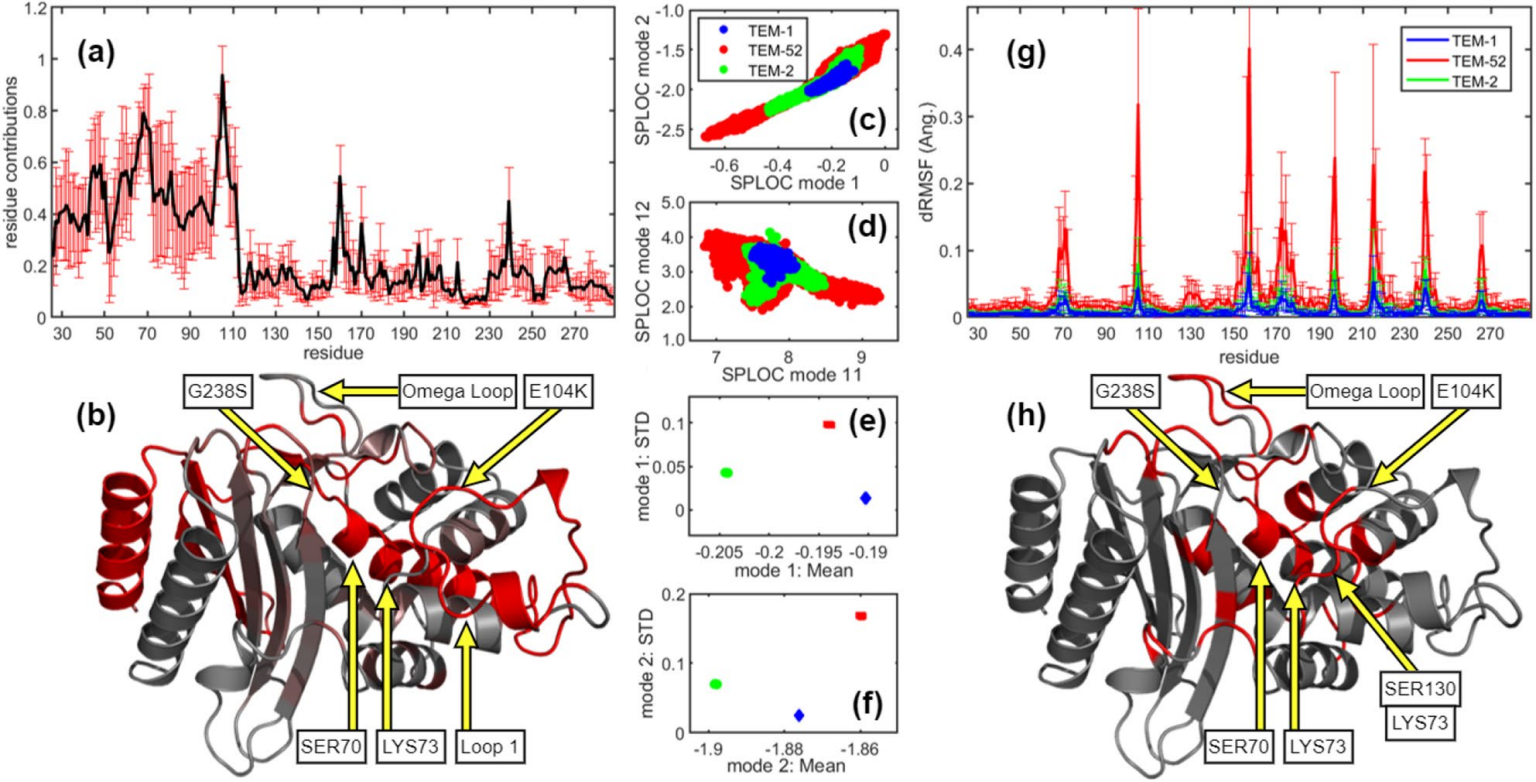
Characterizing extended spectrum resistance from TEM-family beta-lactamase mutants. **(a)** Residue contributions for differences in the functional dynamics between TEM-1 and TEM-52 beta-lactamases. Red error bars show the standard deviation over 10 trials. **(b)** Using pymol with thresholds set at 16% and 37%, the ribbon diagram locates in the structure where functional dynamics are likely to be found (red). Projections of conformational dynamics into **(c)** d-modes 1 and 2, and **(d)** d-modes 11 and 12. Similar to these examples, all d-mode projections of TEM-2 foreshadow TEM-52 behavior with reduced amplitudes. **(e)** MFSP for mode 1. **(f)** MFSP for mode 2. **(g)** The dRMSF with standard deviations is shown for TEM-1, TEM-2 and TEM-52 beta-lactamases, each averaged over 8 MD simulations. The axes in graphs c through g have units of Angstroms. **(h)** A ribbon diagram that highlights in red where dRMSF is 4 or more times greater in TEM-52 compared to TEM-1 within the structure.

Eight 500 ns MD simulations of TEM-1, TEM-2 and TEM-52 were generated. Dynamics was analyzed at the alpha carbon level to provide alignment over 263 residues involving 789 df. SPLOC was trained on TEM-1 as “functional” and TEM-52 as “nonfunctional”. Averaging over ten training trials, $69.2 \pm 12.5$ d-modes, $13.5 \pm 5.9$ u-modes, and $706.3 \pm 11.6$ i-modes were extracted. One trial takes $\approx$ 10 hrs of CPU time on a modern laptop. The discriminant subspace provides DR that captures functionally significant differences in motion between the ESR TEM-52 and non-ESR TEM-1.

Squared d-modes quantify the df responsible for ESR. Summing the squares of the *x*, *y*, *z* components for each carbon-alpha atom gives the residue contributions to ESR, shown in Fig. 6a. The functional dynamics for ESR extend from the N-terminus to the active site, including key catalytic residues SER70 and LYS73 on helix 2, the omega loop, and residues surrounding the mutation site at 104 along the loop region 1. The ribbon diagram in Fig. 6b highlights residue contributions on the beta-lactamase structure, revealing residues known to play an important role in catalytic activity^47^.

The extracted d-modes from each trial correctly classified new bootstrapped samples of TEM-1 and TEM-52, then correctly predicted the unseen TEM-2 to be more like TEM-1 than TEM-52. The greater variance in TEM-52 indicates more conformational space is explored within TEM-52 compared to TEM-1. Typical 2D projections for two sets of d-modes and the MFSP for the top two d-modes are shown in Fig. 6c–f. The differences in STD within the MFSP differentiate functional dynamics, while the scale for differences in mean displacements is insignificant. The close grouping of TEM-1 and TEM-2 correctly suggests they have similar antibiotic resistance profiles. Nevertheless, all d-mode projections of TEM-2 mirror the characteristics of TEM-52, albeit with smaller amplitudes.

Dynamical characteristics of ESR are quantified by the discriminant subspace contribution of the carbon-alpha root mean square fluctuation (RMSF), denoted as dRMSF. The dRMSF is calculated the same way as RMSF^18^, except the conformations are projected into the discriminant subspace using a projection operator comprised of $D_d$ d-modes. An average dRMSF profile over ten trials, along with STD, was calculated for each mutant from 8 MD simulations.

Figure 6g shows the same peak and valley trends in dRMSF for each mutant. In agreement with the literature^48^, dRMSF is greatest on catalytic active residues, serine 70, lysine 73, serine 130, aspartic acid 131, and near the mutation site 104. Furthermore, dRMSF for TEM-52 is much greater than TEM-1, while dRMSF for TEM-2 is slightly greater than TEM-1. Residues in TEM-52 with large dRMSF compared to TEM-1 border the binding pocket, highlighted in Fig. 6h.

The utility of SPLOC-RNN is established by its ability to differentiate two closely related enzymes by functional dynamics, while classifying TEM-2 accurately. The problems with comparative analyses reported previously^49^ entailing laborious effort are removed; replaced by an automated procedure. In addition to identifying key residues known to be critical for ESR, residues 50 and 270 are predicted to be important. These results provide guidance in designing novel antibiotics to withstand mutation pathways in beta-lactamase that cause antibiotic resistance.

## Conclusions and future directions

The projection pursuit machine learning paradigm leads to a novel recurrent neural network architecture for discriminant analysis. A turnkey MATLAB implementation is available to analyze data up to a few thousand variables without limit on sample size. Results on the function recognition pipeline using synthetic molecules, and on data-driven hypothesis development for functional dynamics in extended spectrum beta-lactamase illustrate how MD simulations can be analyzed to guide rational protein and drug design.

### Future directions

A thorough characterization of how sensitivity and selectivity are controlled by the rectified adaptive nonlinear unit (RANU) per perceptron is needed. Straightforward generalization to multi-class discriminant analysis is in progress. Optimized algorithms and new code for parallelization are being developed to support applications surpassing 10,000 variables.

## Methods

### Data packet cubes

The prototypical MD simulation data involves *p* variables (with $p=3N_a$) for the *x*, *y*, *z* coordinates of $N_a$ atoms over *n* frames, resulting in a $p \times n$ data matrix, *X*. A training dataset is defined by $N_F$ data matrices, $X_\alpha$, $\forall \; \alpha = 1,2, ... N_F$ for systems labeled as functional and $N_N$ data matrices, $X_\beta$, $\forall \; \beta = 1,2, ... N_N$ for systems labeled as nonfunctional. To calculate a covariance matrix^50^, MD trajectories are aligned to a reference structure to remove global rotational and translational dynamics. This alignment process is not needed in other applications.

Let $\mu _k$ and $\sigma _k$ define the mean and STD of the projected data on the *k*th mode. The collection of $\mu _k$ and $\sigma _k$ for all modes define a 2*p*-dimensional feature space. Let $\sigma _k = \sqrt{v_k}$, where $v_k$ is the variance. Given that the sample mean, $\mu = {\bar{X}}$, and sample covariance, $C=\frac{1}{n-1} (X - \mu )(X - \mu )^T$, are first and second rank tensors respectively, the mean and variance are readily calculated along any mode direction. Therefore, MD simulation trajectories are organized into functional (class F) and nonfunctional (class N) sets of data packets, respectively given by $\{ \mu _\alpha , C_\alpha \}_{N_F}$ and $\{ \mu _\beta , C_\beta \}_{N_N}$.

### Emergent versus aleatory features

Typical ML methods work within an aleatory perspective where each member of a data stream is classified in a lower dimensional feature space constructed by data projections. Classification occurs when scattered data from different classes group into distinct clusters. Figure 2a shows an example of scattered data that does not separate using aleatory features.

SPLOC classifies a system by the mean and STD of the probability densities (see Fig. 2b,c) along a complete set of modes. The MFSP characterizes emergent properties as shown in Fig. 2d for a d-mode and in Fig. 2e for an i-mode. Although higher order statistics are ignored by tracking only $\mu _k$ and $\sigma _k$ for the k-th mode, extensions to skewness and kurtosis are possible. The output of SPLOC-RNN is a collection of d-modes and i-modes that respectively provide a multivariate description of differences and similarities between systems.

The number of *observations per variable* (OPV) is an important data characteristic for ML performance. Using data packets, there is no explicit dependence on OPV for the time complexity of the calculations, except for constructing the data packets. Nevertheless, training and classification become more accurate as OPV increases because uncertainty in emergent features decreases as $1/ \sqrt{n}$ due to the central limit theorem.

### Signal-to-noise

A scoring function is evaluated for all basis vectors then ordered from largest (rank 1) to smallest (rank *p*). The ranking is used as a mode index, where $S_k$ is the score for the *k*-th mode, such that $S_k \ge S_{k+1}$ $\forall k$. A test for whether two candidates are similar or different is framed as binary classification. The scoring function *bifurcates* the classification decision by setting $S_i$ and $S_d$ as two thresholds, with $S_i < S_d$ given as $S_i=1.3$, and $S_d = 2$. Each mode has three possible outcomes. A mode is said to be a discriminant-mode when $S_k > S_d$ or an indifferent-mode when $S_k < S_i$, corresponding to being clearly different or similar respectively. When $S_i \le S_k \le S_d$ an undetermined-mode occurs.

Let $snr(k | \alpha , \beta ) = | \mu _k(\alpha ) - \mu _k(\beta ) |/\sqrt{ v_k(\alpha ) + v_k(\beta )}$ define the signal-to-noise ratio for the *k*-th mode when comparing the $\alpha$-th functional system to the $\beta$-th nonfunctional system, and $sbn(k | \alpha , \beta ) = \max {(0,snr(k | \alpha , \beta ) - 1)}$ is signal beyond noise. Let $rex(k | \alpha , \beta ) = \max { (\sigma _k(\alpha ) / \sigma _k(\beta ) , \sigma _k(\beta ) / \sigma _k(\alpha ) ) } -1$ be the excess ratio of STD from the two systems being compared. Let $S_m = \sqrt{S_i S_d}$ be the geometric mean of the two thresholds, representing a bifurcation reference. With the *k*, $\alpha$ and $\beta$ dependencies suppressed in the functions *snr*, *sbn* and *rex*, the scoring function is defined as:

1 $$\begin{aligned} S_k(\alpha ,\beta ) = \left\{ \begin{array}{lr} \sqrt{sbn^2 + rex^2} + 1 &{} \quad \text{ when } > S_d \\ \sqrt{snr^2 + rex^2} + 1 &{} \quad \text{ when } < S_i \\ S_m &{} \quad \text{ otherwise } \\ \end{array} \right. \end{aligned}$$

The greater or less than conditions in the piecewise function for $S_k$ in Eq. (1) are mutually exclusive because $sbn < snr$. Note that $S_k(\alpha , \beta )$ enforces conservative decisions by using more demanding threshold conditions. Upon failure of an indisputable decision, the score of $S_m$ represents maximum uncertainty. The score for the *k*-th mode is given by $S_k = \exp { ( \, \langle \ln { S_k(\alpha , \beta ) } \rangle _{\alpha ,\beta } \, } )$. The averaging process, denoted by $\langle \cdot \rangle _{\alpha ,\beta }$, is over all $N_F \times N_N$ pairs of functional and nonfunctional systems being compared.

### Statistical significance

The mean score of a mode is sensitive to outliers when the contribution from a pair of functional and nonfunctional systems dominate the average. To mitigate false positives resulting from fluctuations, two sigmoid-like vote activation functions are defined: $f_d$ and $f_i$ for d-modes and i-modes respectively. Using $\ln { S_k }$ as the argument, these two functions are shown in Fig. 2f. Note that $f_d = 0.5$ and $f_i = 0.5$ at the boundaries of an indecision region. Conditional consensus is calculated over data packets as either $V_d = \langle f_d \rangle _{\alpha ,\beta }$ or $V_i = \langle f_i \rangle _{\alpha ,\beta }$ by assuming the basis vector is respectively a d-mode or an i-mode.

A score is statistically significant when a consensus vote exceeds a threshold, $V_t$. Using a data-driven heuristic formula, $V_t$ is automatically adjusted. Due to difficulty weighting uncertainties across data packets with varying number of samples, all data packets are restricted to have equal sampling. However, $N_F$ need not equal $N_N$, as expected in drug discovery there will typically be a class imbalance with $N_N > N_F$.

### Clustering quality

The emergent features of a data packet for the *k*-th mode are specified by a single point in an MFSP, given as $(\mu _k, \sigma _k )$. A set of scattered points reflect $N_F$ functional and $N_N$ nonfunctional data packets. How these points cluster is important to quantify. Discriminant and indifferent cluster quality factors are respectively defined as $Q_d(k)$ and $Q_i(k)$ to assess clustering properties within the MFSP for the *k*-th mode. For accurate classification, the MFSP cluster quality factor is required to exceed a minimum quality threshold, $Q_m$. Exemplar high quality clustering for a d-mode and i-mode is shown in Fig. 2d,e respectively.

The $Q_d(k)$ and $Q_i(k)$ cluster quality factors involving ratios of geometrical properties form scale invariant measures. For an i-mode, all points from functional and nonfunctional data packets cluster tightly. For a d-mode, the points in an MFSP linearly separate the two classes by forming a gap in at least one feature. High quality for $Q_d$ implies the gap is much larger than within-cluster scatter. Although the quality of clustering improves as within-class scatter is minimized, both clusters need not exhibit compact within-class scatter. For example, consider an MFSP describing the mechanism of action within an enzyme. A nonfunctional mutant only needs to be void of this mechanism, with no implication that nonfunctional molecules share similar dynamical features.

### Feature extraction

The efficacy of a perceptron is modeled using a rectified adaptive nonlinear unit (RANU). For the *k*-th mode, the RANU is given by

2 $$\begin{aligned} \displaystyle E_k = {\left\{ \begin{array}{ll} Q_d(k) \times r_d(x) &{} \text {if }S_k > S_m\\ Q_i(k) \times r_i(x) &{} \text {if }S_k < S_m \end{array}\right. } \end{aligned}$$

where the quality factors $Q_d(k)$ and $Q_i(k)$ govern the strength of rectification, and the functions $r_d$ and $r_i$ quantify relevance. A mode is more relevant as $S_k$ deviates farther from the bifurcation reference, $S_m$. Relevance is modeled as a function of *x*, where $x = |\ln (S_k/S_m)|$. A linear rectifier is recovered when $r_d(x) = r_i(x) = x$. The nonlinear functions used in SPLOC-RNN are shown in Fig. 2g.

### Feature selection

The decision tree shown in Fig. 2h is applied on each basis vector during training. The complete set of orthonormal basis vectors is partitioned into three subspaces sorted by the decision triad. When true, thresholds are met on signal-to-noise, statistical significance, and clustering quality concurrently to establish *qualification*. Basis vectors that fail the decision triad span the undetermined subspace, referred to as u-modes.

The decision triad identifies d-modes, u-modes and i-modes, with the discriminant and indifferent subspaces elucidating mechanistic details of how functional and nonfunctional systems are different and similar respectively. Calculations are faster when u-modes are dropped to reduce dimensionality, isolating more relevant variables. However, this risks the removal of latent information. Therefore, adaptive importance sampling is employed to balance speed and accuracy.

### Competitive learning

Directed orthogonal rotations are recurrently applied to pairs of modes. Consider modes *a* and *b*. Their combined efficacy of $(E_a + E_b)$ is denoted as $E_{ab}( \theta )$, where the modes are rotated within a plane using a 2D rotation matrix, $R(\theta )$. Paired perceptrons have an intense rivalry due to the nonlinearity in the RANU as their mode directions rotate within a plane. The perceptron with greater efficacy grows at the expense of the other. Successive spinning of mode directions increases efficacy and promotes a scree shape in the signal-to-noise relevance over all modes.

The numerical process first projects the *p*-component vector, $\mu$, into a plane defined by modes *a* and *b*. Next, the $p \times p$ covariance matrix is reduced to a $2 \times 2$ covariance matrix that describes covariance in this plane; this reduction has a complexity of $p^2$. Thereafter, regardless of system size a derivative-free search is employed that maximizes $E_{ab}(\theta )$ as 2D rotations are performed to calculate the optimal mean and variance. Successively applying optimal orthogonal rotations on mode pairs is tantamount to performing factor analysis^51^. This process monotonically increases efficacy of the perceptron network. Projecting the initial two *p*-dimensional vectors into a 2D subspace and reconstructing the two final 2D vectors back to *p*-dimensional vectors has a complexity of *p*.

### Importance sampling

Importance sampling is based on prior history of monitoring successes and failures for spinning pairs of modes. All prior history is erased per epoch. To maximize network efficacy, more than one spin per distinct pair of modes is generally required. However, a tiny fraction of the $p(p-1)/2$ distinct mode pairs is considered in one epoch. A spin is unproductive when it yields a negligible increase in efficacy. A small tilt angle between the current and previous planes formed by a pair of modes (*a* and *b*) leads to an unproductive spin. Therefore, it is critical to control the spin rate of each distinct pair of modes for efficient training performance.

Without importance sampling, mode *a* is iterated from 1 to *p* by an outer loop. An inner loop sweeps over mode *b*, from 1 to *p* with $b \ne a$. Importance sampling employs two ergodic stochastic processes, each governed by kinetic equations. First, mode pairs with high probability to yield an unproductive spin are skipped during a sweep. Second, the outer loop is replaced by selecting mode *a* from a prioritized queue that favors modes with greater past efficacy yields.

Starting from a randomized initialization per epoch, spin rates tend toward kinetic equilibrium. The kinetic equations drive d-modes and i-modes with greater efficacy to converge more rapidly. This accelerates network convergence because the effective dimension decreases with continuing iterations. Convergence is reached when the percent increase in network efficacy is less than 5% for three successive epochs.

### Creative exploration

The frequency of *directed* orthogonal rotations (DOR) applied to u-modes in competitive learning is greatly reduced due to importance sampling. To mitigate the risk of missing latent information, *undirected* orthogonal rotations (UOR) are applied to inferior u-modes as a source of random noise before each sweep. Random rotations are generated using a Cayley transformaion^52^ and applied on a randomized subset of u-modes. Successive UOR produce a random walk in basis vector directions, yielding diffusive exploration within the undetermined subspace *without judgement*. This causes d-modes and i-modes to appear stochastically, while increasing tilt angles between current and previous pairs of modes. Random noise is a source of *creativity* that enables barriers in perception to be crossed. A transduction of creativity to perception takes place by the RANU as UOR extracts unstructured latent information, and DOR drives u-modes with improved quality to pass the decision triad filter.

### Discovery likelihood

A discriminant subspace of dimension $D_d > 1$ elucidates the *multivariate attributes* that differentiate functional and nonfunctional systems in the training set. For $D_d > 0$, SPLOC-RNN classifies the training data perfectly due to the decision triad. Therefore, obtaining a null discriminant subspace indicates either more samples are needed to increase OPV, most variables are irrelevant, or higher order statistics are required to detect differences.

To quantify functional (F) and nonfunctional (N) characteristics for the *k*-th d-mode, a set of univariate probability density functions (PDFs) are calculated^53^ as $f_F( x_k | k )$ and $f_N( x_k | k )$ respectively. Here, $x_k$ is a random variable characterizing a projection in the *k*-th mode, $\forall \, k$, ranging from 1 to $D_d$. The $(N_F + N_N) \times D_d$ different PDFs quantify key factors necessary for a system to function in a comparative context. In a molecular design scenario, consider $N_U$ simulated systems proposed to be functional. The data from these simulations are projected into d-modes to yield $N_U \times D_d$ PDFs given by $f_U( x_k | k )$. From Bayesian inference, *p*(*U*, *k*) and *q*(*U*, *k*) respectively give the probability that an unknown system *U* is functional and not nonfunctional.

The product, $p(U,k) \, q(U,k)$ sets a baseline likelihood for system *U* to be functional and not nonfunctional with respect to the *k*-th mode. Small *q*(*U*, *k*) implies small *p*(*U*, *k*). However, as $q(U,k) \rightarrow 1$, *p*(*U*, *k*) has a range on [0, 1] because not all differences between *F* and *N* systems are functionally relevant. It is desirable to have a *U* system with (many, few) similar characteristics to *F* systems, resulting in a relatively (high, low) DL. A much lower DL occurs when system *U* has similar characteristics to any *N* system. When characteristics of system *U* differ from systems *F* and *N*: $\text{ DL } \rightarrow 1$ because it is prudent to search unseen examples for the discovery of functionally relevant characteristics. Defining $\text{ DL } = \left[ 1 - {\bar{p}}(U,k) \, {\bar{q}}(U,k) \right] ^2$ achieves all these desired properties, giving a bias toward exploring unknown situations.

### Multiple solutions

The perception of high dimensional data is determined by *p* projections from a complete orthonormal basis set. The way data is perceived depends on the viewpoint for how the data is interpreted. SPLOC has three operational modes ($M_-$, $M_0$ and $M_+$) that control the viewpoint by modifying the RANU. To learn what is similar between systems and identify conserved mechanisms, Eq. (2) is modified by setting $Q_d = -0.1$ in operational mode $M_-$. When differences are of upmost importance, Eq. (2) is modified by setting $Q_i = -0.1$ in operational mode $M_+$. In operational mode $M_0$, the RANU defined in Eq. (2) extracts similarities and differences simultaneously.

Incompatible perceptions to varying degrees occur when the basis vectors of different solutions are not shared. Obtaining a different perception depending on viewpoint is analogous to the incompatibility of certain simultaneous measurements found in quantum theory. The fundamental origin of multiple perceptions derives from linear algebra, manifesting as different complete basis sets depending on the RANU, which controls selectivity and sensitivity.

After the rectifying function is selected to answer a question of interest, a basis set that yields a local maximum is a solution. As an inverse problem, SPLOC generally extracts multiple solutions consistent with the training data. The initial basis set creates a *preconceived bias* that may influence solutions. Multiple solutions generate competing data-driven hypotheses for the underlying mechanisms leading to similar classification results^54^. Obtaining a consensus over an ensemble of solutions provides a statistically sound method to reach an informed conclusion with quantitative confidence levels.

### Initial basis set and training protocols

Any orthonormal complete basis set can be specified as input for the initial basis set. Otherwise, two options are available. (1) A standard basis set is used corresponding to the original variables. (2) PCA is applied to three sets of pooled data, where (all functional, all nonfunctional, all) systems are pooled separately. The complete set of PCA-modes from the pooling case that maximizes the objective function is selected as the initial basis set. Option 2 is employed in this work.

Multiple data packets from a single data stream can be created in two ways: *Partitioning* divides a system with *n* samples into *m* non-overlapping sets of *n*/*m* samples yielding *m* data packets for a single system. *Bootstrapping* shuffles *n* samples, then uses each half (or another defined subset) as a data packet. Repeat shuffling generates replicas to increase the number of data packets representing a single system.

A prudent training protocol is to first train using operational mode $M_+$. Second, using the $M_+$ output basis set as an initial perception, train further using operational mode $M_0$. Except for the egg hunt benchmark, all other training has been done as a 1 step process in operational mode $M_0$.

### Iris/wine data packets

There are 50 samples of Setosa, Virginica and Versicolor classes in the Iris dataset. The data was reconfigured into data packets by randomly selecting 25 samples from Setosa (*F*) and Virginica (*N*). This data partition is then bootstrapped by creating $N_F = N_N = 30$ data packets, each with $n=10$ samples obtained by random subsampling 10 of the 25 samples with replacement for classes *F* and *N*. Using the same procedure, 30 data packets were created by subsampling 10 of the remaining 25 samples that comprise the testing set for Setosa and Virginica, and $N_U = 30$ data packets for unlabeled systems created by subsampling 10 of 50 samples from the third class Versicolor. The wine dataset consists of three classes (1, 2, 3) respectively with (59, 71, 48) samples and labeled as (*F*, *N*, *U*). The same procedure was used to obtain $N_F = N_N = N_U = 30$ data packets all containing $n=15$ samples.

### Egg hunt setup

The SGN covariance matrix is a *p*-dimensional identity matrix. The CGN covariance matrix is constructed in two steps. First, the diagonal elements are given as: $C_{jj} = 1/\sqrt{j}$. Second, the off-diagonal elements are populated as $C_{ij} = \frac{ C_{ii} }{ \sqrt{|i-j|} } \forall j > i$, where $C_{ji} = C_{ij}$. Then $n = \text{ OPV } \times p$ is the number of samples generated to build a $p \times n$ data matrix.

Placing an “egg” means the concealing environment is modified to embed a signal. The variable at the 80-th percentile is selected along with the proceeding 5 variables to define a 6-dimensional (6D) subspace. For example, if $p=100$, variables 75 through 80 are selected, and for $p=10$, variables 3 through 8 are selected. A $6 \times 6$ submatrix of matrix *C* gives the covariance for the 6D subspace. Diagonalizing the submatrix yields 6 orthogonal eigenvectors with corresponding eigenvalues ${v_1, v_2, ... , v_6}$ labeled in descending order of variance. The eigenvectors are used to express the data within the 6D subspace along the 6 principal coordinates. A large egg is placed in a plane defined by the first two eigenvectors, while a small egg is placed in a plane defined by the last two eigenvectors. The STD for each direction is scaled by a factor of 4, then the data only within this plane is regenerated. The final step rotates the data back into the original coordinates to scramble the egg.

The training protocol consists of three steps. Given $n = \text{ OPV } \times p$ samples, the data is divided into three partitions, labeled P1, P2 and P3. P1 contains all samples, while P2 has two data packets each with 1/2 of the samples, and P3 has three data packets each with 1/3 of the samples. Functional and nonfunctional systems each have these three partitions available. Operational mode $M_+$ was selected to find d-modes on the first step using P1, then operational mode $M_0$ was used on the next two steps using P2 and P3.

### Synthetic molecule dynamics

Atomic bonds are modeled by harmonic interactions. Short-range pairwise repulsive interactions prevent atomic clashing. Geometrical shapes are maintained by weak harmonic restraints. Monte Carlo simulation was employed to generate a 500 and 20,000 frame trajectory per molecule.

### Beta-lactamase dataset

From the protein data bank, eight structures with PDB-codes (1ERM, 1ERO, 1ERQ, 1HTZ, 1JWP, 1LHY, 1XPB, 3JYI) were computationally mutated as needed to create 8 initial structures for TEM-1, TEM-52 and TEM-2 mutants having 263 residues. A 500 ns MD production run was performed on each mutant in apo form. Collecting one frame every 50 ps produces 10,000 frames. Simulations were done with GROMACS in explicit TIP3P water using previously described protocols^49^. For each trajectory, 10,000 frames are randomly shuffled, and the first 5000 frames were combined per mutant resulting in 40,000 samples for each data packet. This process creates 16 data packets per mutant, each with 50.7 OPV.

## Supplementary information

Supplementary Information.
