# Supplementary material for: Molecular function recognition by supervised projection pursuit machine learning
Source: Sci Rep. 2021 Feb 19;11:4247. doi: 10.1038/s41598-021-83269-y (PMC7895977; doi:10.1038/s41598-021-83269-y)
Supplement: Supplementary file 1 — Supplementary Information. [file 41598_2021_83269_MOESM1_ESM.pdf]

Supplementary Information for

Molecular Function Recognition by Supervised Projection Pursuit Machine Learning

Tyler Grear, Chris Avery, John Patterson and Donald J. Jacobs

Donald J. Jacobs.
E-mail: djacobs1@uncc.edu

This PDF file includes:

Supplementary Technical Details
Supplementary Figures S1 to S18
Supplementary Tables S1 to S7
Supplementary References

Technical Details

Separable Objective Function. Implemented as an iterative process to change the directions of a complete set of basis vectors, projection pursuit (PP) is employed to maximize an objective function. Using Dirac bra-ket notation, the i-th basis vector on the s-th step is denoted as $|i,s\rangle$. Note that the "ket", denoted as $|i,s\rangle$, represents a column vector. The "bra", denoted as $\langle i,s|$, represents a row vector. The molecular conformation of N_a atoms is described by a single state vector having $3N_a$ components. The complete set of vectors $\{\,|i,s\rangle\,\}\ \forall\, i = 1, 3N_a$ span the $3N_a$ dimensional space. Let $p = 3N_a$ as the number of variables in the system. Through successive orthogonal rotations, the set of basis vectors remain orthonormal and complete, $\forall\, s$, such that $\langle j,s|i,s\rangle = \delta_{ji}$ and $\sum_{i=1}^{p} |i,s\rangle\langle i,s| = I$, where δ_{ji} is the Kronecker delta and I is the identity matrix. A scoring function indexes the basis vectors, which are subsequently rank-ordered from highest to lowest score. A basis vector is referred to as a mode after it is indexed. The objective function represents efficacy, E, which is separable such that $E = \sum_{k=1}^{p} E_k$, where E_k is the efficacy of the k-th mode.

The function E_k depends on features of the projected data with respect to the k-th mode only. Therefore, it is convenient to consider two arbitrary modes, $|a\rangle$ and $|b\rangle$, that define a plane. An orthogonal rotation on these two vectors within the plane will generally change E_a and E_b, while mode efficacy for all other modes remain constant. By choosing the rotation angle that maximizes $(E_a + E_b)$, total efficacy monotonically increases. This strategy is found in factor analysis, which can be viewed as a form of PP where a specific objective function is being maximized. The objective function and the method of optimizing the objective function is what sets apart the various PP methods. Herein, the mathematical details underlying the supervised projective learning with orthogonal completeness (SPLOC), and as a recurrent neural network (SPLOC-RNN) are explained.

Signal-to-noise. A scoring function is evaluated for all basis vectors then ordered from largest (rank 1) to smallest (rank p). The ranking is used as a mode index, where S_k is the score for the k-th mode, such that $S_k \geq S_{k+1}\ \forall k$. A test for whether two candidates are similar or different is framed in binary classification. The scoring function *bifurcates* the classification decision by setting S_i and S_d as two thresholds, with $S_i < S_d$ given as $S_i = 1.3$, and $S_d = 2$. Each mode has three possible outcomes. A mode is said to be a discriminant mode when $S_k > S_d$ or an indifferent mode when $S_k < S_i$, corresponding to being clearly different or similar respectively. When $\underline{S_i \leq S_k \leq S_d}$ an undetermined mode occurs.

Let $snr(k|\alpha,\beta) = |\mu_k(\alpha) - \mu_k(\beta)|/\sqrt{v_k(\alpha) + v_k(\beta)}$ define the signal-to-noise ratio for the k-th mode when comparing the α-th functional system to the β-th nonfunctional system, and $sbn(k|\alpha,\beta) = \max\left(0, snr(k|\alpha,\beta) - 1\right)$ is signal beyond noise. Let $rex(k|\alpha,\beta) = \max\left(\sigma_k(\alpha)/\sigma_k(\beta), \sigma_k(\beta)/\sigma_k(\alpha)\right) - 1$ be the excess ratio of STD from the two systems being compared. Let $S_m = \sqrt{S_i S_d}$ be the geometric mean of the two thresholds, representing a bifurcation reference. With the k, α and β dependencies suppressed in the functions snr, sbn and rex, the scoring function is defined as:

$$S_k(\alpha,\beta) = \begin{cases} \sqrt{sbn^2 + rex^2} + 1 & \text{when} > S_d \\ \sqrt{snr^2 + rex^2} + 1 & \text{when} < S_i \\ S_m & \text{otherwise} \end{cases} \qquad [1]$$

The greater or less than conditions in the piecewise function for S_k in equation 1 are mutually exclusive because $sbn < snr$. Note that $S_k(\alpha,\beta)$ enforces conservative decisions by using more demanding threshold conditions. Upon failure of an indisputable decision, the score of S_m represents maximum uncertainty. The score for the k-th mode is given by $S_k = \exp\left(\,\langle \ln S_k(\alpha,\beta)\rangle_{\alpha,\beta}\,\right)$. The averaging process, denoted by $\langle\cdot\rangle_{\alpha,\beta}$, is over all $N_F \times N_N$ pairs of functional and nonfunctional systems being compared.

Statistical significance. Outliers influence the mean score of a mode when the contribution from a pair of functional and nonfunctional systems dominate the average. To mitigate false positives resulting from fluctuations, a voting activation function (VAF) is introduced. A consensus vote accepts or rejects a proposal from the scoring function as to whether a basis vector is a discriminant-mode (d-mode) or an indifferent-mode (i-mode). Let f_d and f_i define a VAF for d-modes and i-modes respectively. Both f_d and f_i are calculated for each functional and nonfunctional system comparison.

Two conditional consensus votes are calculated as either $V_d = \langle f_d\rangle_{\alpha,\beta}$ or $V_i = \langle f_i\rangle_{\alpha,\beta}$ assuming the basis vector is respectively a d-mode or an i-mode. Let $x = \ln S_k(\alpha,\beta)$, $x_d = \ln S_d$ and $x_i = \ln S_i$. Further, let $p_d(x) = \max\left[1, (2 - x/x_d)\right]$ and $p_i(x) = \min\left[1, (x/x_i)^2\right]$. The VAFs are given as:

$$f_d(x) = \left[1 + \exp\left(16(x_d - x)\right)\right]^{-p_d(x)} \qquad [2]$$

$$f_i(x) = \left[1 - \left[1 + \exp\left(16(x_i - x)\right)\right]^{-1}\right]^{p_i(x)} \qquad [3]$$

The powers $p_d(x)$ and $p_i(x)$ ensure the functions $f_d(x)$ and $f_i(x)$ change more rapidly than a sigmoid function when respectively $x < x_d$ and $x < x_i$.

A score is statistically significant when the relevant consensus vote exceeds a threshold, V_t. Arguably, V_t should depend on the number of samples in each data-packet along with the number of data-packets being compared. Due to difficulty weighting uncertainties across data-packets with varying number of samples, all data-packets are restricted to have equal sampling. However, N_F need not equal N_N, allowing for class imbalance in data-packets.

A heuristic formula to determine V_t has been implemented. Qualitatively, V_t should be a slowly varying function with a range between 0.5 and 1, depending on N_F, N_N, n and p. For practical considerations, 0.95 sets the maximum level. As $n \to \infty$,

a majority vote is satisfied when $V_t = 50\%$. For finite sample size V_t increases as n decreases, this safeguards against overfitting due to fluctuations. For strong data imbalances (e.g. $N_F \gg N_N$ or $N_F \ll N_N$), uncertainty will be tied to $\min(N_F, N_N)$. When p increases with fixed sampling, V_t increases to account for more fluctuations as OPV decreases.

Guided by the desired trends just summarized, let $y = p/N_{eff}$; $n_1 = n\sqrt{N_F}$; $n_0 = n\sqrt{N_N}$; $a = \min[n_1, n_0]$ and $b = \max[n_1, n_0]$. Then an effective sample number is given as $N_{eff} = 0.5(a+a(2b/(a+b))^2)$. Let $p_v = \max[0, 0.5 - 0.49999\sqrt{p/N_{eff}}]$. The heuristic vote threshold is given by:

$$V_t = \min\left(0.95, 0.5 + 0.7 * (p/N_{eff})^{p_v}\right) \qquad [4]$$

The VAF increases as the signal-to-noise score deviates farther from the bifurcating reference. If the conditional consensus vote does not exceed V_t, the basis vector is placed in the undetermined subspace.

Clustering quality. The two features, μ_k and σ_k, characterize how a data-packet projects onto the k-th mode. Together, (μ_k, σ_k) is a point on a plane that is a cross-section in a $2p$-dimensional feature space. Each mode produces a scatter plot in a mode feature space plane (MFSP) showing N_F functional and N_N nonfunctional systems. To achieve accurate classification, quality clustering is enforced by requiring the clustering characteristics within each MFSP to exceed a minimum quality threshold, Q_m.

The desirable clustering characteristics depend on the type of mode. A d-mode discerns differences between functional and nonfunctional systems, while an i-mode discerns similarities. Discriminant and indifferent quality factors are defined as $Q_d(k)$ and $Q_i(k)$ respectively for the k-th mode. Geometrical properties of the scattered points from two different classes within a MFSP are used to calculate quality factors. Among many models tested, nonlinear functions for $Q_d(k)$ and $Q_i(k)$ performed better. For example, if two attributes (say $a_1 \geq 0$ and $a_2 \geq 0$) are desirable, then the function $a_1 + a_2 + a_1 a_2$ is more effective than $a_1 + a_2$ by inducing cooperative behavior. Both $Q_d(k)$ and $Q_i(k)$ are symmetric functions with respect to an exchange of labels (e.g. functional $\rightleftarrows$ nonfunctional). With ample plasticity in how $Q_d(k)$ and $Q_i(k)$ can be defined, certain conditions found to be necessary are described next.

Cluster quality is expressed by ratios of geometrical properties to form scale invariant measures. For a d-mode, the points in a MFSP must separate the two classes by forming a gap in at least one feature. Furthermore, the points within at least one class must cluster tightly. Although the quality of clustering improves when the spread among points within both classes is small relative to the gap that separates them, both clusters need not exhibit compact within-class scatter. The only requirement is that one class linearly separates from the other within the MFSP for any d-mode.

To detect two classes, at least one tight cluster (or preferably two tight clusters) should form with a gap between the points from different classes. The gap length should be larger than the smallest within-class spread. It is instructive to compare d-mode clustering in a MFSP to clustering by LDA and SVM. Within-class variance is minimized for each class in LDA, limiting its utility whenever there is asymmetry in variance between classes. LDA does not guarantee a gap will form between clusters of different classes. In SVM, a hard or soft boundary between clusters of two classes can be obtained, but within-class variance is unconstrained.

Quality clustering for an i-mode ensures the scattered points from both classes within an MFSP mix well, where there is no linear separation, and no gap. Minimizing the spread of scatter improves the quality factor, but due to scale invariance, there is no absolute length scale. The critical criterion is to optimize the mixing of both classes within an MFSP.

Feature extraction. Each basis vector is assigned one perceptron that attempts to maximize mode efficacy using a rectified adaptive nonlinear unit (RANU). Mode efficacy quantifies how well binary linear classification is performed within the MFSP. The total efficacy of the perceptron network quantifies how well a complete basis set stratifies p-dimensional data into different and similar emergent features across all systems.

Mode efficacy as a RANU for the k-th mode is given by

$$E_k = \begin{cases} Q_d(k) \times r_d(x) & \text{if } S_k > S_m \\ Q_i(k) \times r_i(x) & \text{if } S_k < S_m \end{cases} \qquad [5]$$

where the quality factors, $Q_d(k)$ and $Q_i(k)$ govern the strength of rectification, and the functions r_d and r_i quantify relevance. A mode is more relevant as S_k deviates farther from the bifurcation reference, S_m. Relevance is modeled as a function of x, where $x = |\ln(S_k/S_m)|$. A linear rectifier is recovered when $r_d(x) = r_i(x) = x$.

To achieve greater discrimination, higher propensity is given for the extraction of d-modes compared to i-modes. When $x \ll 1$, a $\sqrt{x}$ term provides more aggressive movement away from the bifurcation reference compared to x. For $x \gg 1$, higher powers of x prolong aggressive movement away from S_m. This is important because S_k has a limited range it can displace from S_m for $S_k < S_m$, whereas for $S_k > S_m$ the range is unbounded. From these considerations, the functions for relevance are given as $r_d(x) = \sqrt{x} + x(1+x)$ and $r_i(z) = (\sqrt{z} + z(1+z))/10$ where $z = 6x^2 + 28x^4$.

Competitive learning. The learning process is competitive by employing *directed* orthogonal rotations recurrently to pairs of modes. Consider modes $|a\rangle$ and $|b\rangle$. Their combined efficacy of $(E_a + E_b)$ is denoted as $E_{ab}(\theta)$, where the modes are rotated within a plane using a rotation matrix, $R(\theta)$. The angle of rotation, θ, is limited to the range $[0, \pi/2)$ because all other angles fall into this range with a suitable swap in mode labeling and/or sign change. At θ^*, where E_{ab} is a maximum, the optimal modes are expressed as:

$$\begin{pmatrix} |a'\rangle \\ |b'\rangle \end{pmatrix} = R(\theta)\big|_{\theta^*} \begin{pmatrix} |a\rangle \\ |b\rangle \end{pmatrix} = \begin{pmatrix} cos\theta^* & -sin\theta^* \\ sin\theta^* & cos\theta^* \end{pmatrix} \begin{pmatrix} |a\rangle \\ |b\rangle \end{pmatrix} \quad [6]$$

In numerical calculations the p-component vector, μ, is projected into a plane, and the $p \times p$ covariance matrix is reduced to a 2×2 covariance matrix, such that

$$\mu \rightarrow \mu' = \begin{pmatrix} \langle a|\mu\rangle \\ \langle b|\mu\rangle \end{pmatrix} \qquad Q \rightarrow Q' = \begin{pmatrix} \langle a|Q|a\rangle & \langle a|Q|b\rangle \\ \langle b|Q|a\rangle & \langle b|Q|b\rangle \end{pmatrix} \quad [7]$$

Rotations are numerically performed using $R\mu'$ and $RQ'R^T$ in order to calculate the mean and variance respectively for new mode directions. Using a derivative-free search, $E_{ab}(\theta)$ is maximized as θ is scanned within a plane regardless of system size. Only the first step of equation 7 and the last step of equation 6 require full size vectors. Reducing the covariance matrix to a 2×2 matrix is the rate limiting step.

Importance sampling. Importance sampling is based on prior history of monitoring successes and failures for spinning pairs of modes. At each epoch all importance sampling variables are initialized to erase any prior history. More than one spin per distinct pair of modes will generally be required before network efficacy is maximized. Controlling how frequent to spin a pair of modes is critical because ensuring the intermittent spinning of each mode when paired to different mode partners is necessary to converge rapidly. Only a fraction of the distinct mode pairs given by $p(p-1)/2$ is considered in one epoch. Convergence is reached when the percent increase in network efficacy is less than 5% for three successive epochs.

A single spin is unproductive when the gain in efficacy is less than 0.01. A spin is more likely to increase efficacy as the tilt angle increases between the current and previous planes formed by a given pair of modes. How often to spin a pair of modes within an epoch is governed by a scheduling process, involving at least $\sqrt{2p}p$ spins and no more than $5\sqrt{2p}p$ spins. Without importance sampling, mode a is iterated from 1 to p by an outer loop. An inner loop sweeps over mode b, from 1 to p with $b \neq a$. Importance sampling modifies the spin selection process in two ways. First, mode pairs likely to yield an unproductive spin are skipped during a sweep. Second, the outer loop is replaced by selecting mode a from a prioritized queue. Two separate mechanistic models for the inner and outer loops are implemented to facilitate importance sampling.

A symmetric $p \times p$ matrix stores the spin-probability, $P(b,a)$, to spin the proposed pair of modes (a and b). When $P(b,a) > \frac{1}{2}$ a spin is executed, otherwise it is skipped. The initial condition given by:

$$P(a,b) = P(b,a) = \frac{1}{8} + \frac{7}{8} r^{(1.38 \ln(p) - 0.4)} \quad [8]$$

is applied per epoch, where r is a random number on $(0,1)$. This heuristic formula is an unbiased randomization for mode pairs to have high or low probability to spin while keeping the initial number of mode pairs to spin low.

Two conditions inform how to update the spin-probability:

$$\begin{cases} \text{if } P(a,b) > \frac{1}{2} \text{ then} & P(a,b) \rightarrow w_- P(a,b) \\ \text{otherwise} & P(a,b) \rightarrow \min[1, w_+ P(a,b)] \end{cases} \quad [9]$$

First consider setting $w_+ = 2^{1/5}$ and $w_- = \frac{1}{4}$. With $w_+^{10} = w_-$, equation 9 creates a delay of 10 interim skips before the next spin is executed on the same pair of modes. This delay increases the likelihood that a greater tilt angle between the current and previous planes will form.

To facilitate data-driven control let $w_- \rightarrow \frac{1}{4} w_0^{p_a + p_b}$ to obtain a generalized model. This model retains a minimum delay, while the p_a and p_b variables further reduce the spin-probability for the following reasons: mode directions converge, the decision triad consistently fails, or mode efficacy is low. Past history adaptively reduces w_0 (from its maximum value of 1) in proportion to the number of consecutive times a spin is unproductive. This mechanistic model creates an ergodic process where the initial spin-probability distribution evolves toward dynamical equilibrium.

A second mechanistic model adaptively modifies the probability to select mode a fixed over a sweep. The model assigns a waiting time, $T(k)$, and a time step, $\delta T(k)$, to each mode. Initially, $T(k) = 0$ and $\delta T(k) = 1 \; \forall \, k$. A global waiting time, T_g, is iteratively determined, with $T_g = 1$ initially. A while-loop is performed, where mode a is selected from 1 to p in one of three ways explained below. Regardless of the selection method, the waiting time is modified as $T(k) \rightarrow T(k) - \delta T(k)$. After this update, if $T(k) < 0$ the selection is accepted, otherwise another selection is made. Past performance is tracked based on the average change in efficacy per mode $\langle \delta E \rangle$ over the previous sweep.

Regular updates are then applied by tracking the global time, with $T_g \rightarrow 0.9 T_g + 0.1 \langle \delta E \rangle$, and the remaining waiting time for mode a will decrease by the increment $\delta T(a) \rightarrow \min[T_g/100, \langle \delta E \rangle]$. Furthermore, whenever the mode a is selected as the outer loop choice, the total time for waiting must be reset, which is done using the global mean waiting time, such that $T(a) \rightarrow T_g$. The process from this mechanism is also ergodic. Additional biases can be included as well depending on the selection method.

Three selection methods have been implemented. The selection method is either random (unbiased) with probability P_o or biased toward high or low ranking modes, each with probability $(1 - P_o)/2$. The probability for each selection method is adaptive, such that low success rates drive $P_o \rightarrow 1$ and high success rates drive $P_o \rightarrow 0$ to favor extreme limits of the spectrum. This bias drives the most significant d-modes and i-modes to converge with higher priority. As convergence is achieved starting at the wings of the spectrum and working inward, the effective dimension of the system decreases to accelerate the overall convergence rate.

Creative exploration. As an initial step of the outer loop described above, *undirected* orthogonal rotations (UOR) are applied to a subset of u-modes prior to each sweep as a source for random noise. The probability to select a u-mode is heuristically defined as

$$P_u(k_u) = \max[0, (1 - 16(S_m - S(k_u))^2] \times \max[0, (1 - (\,2V(k_u)\,)^2] \times \max[0, (1 - \sqrt{10E(k_u)})] \quad [10]$$

where $S(k_u)$ is the scoring function, $V(k_u)$ is the consensus vote, and $E(k_u)$ is the efficacy for a u-mode with index k_u. Let r be a uniform random number on $(0, 1)$. When $r < P_u(k_u)$, the k_u mode is selected to have its direction perturbed by random noise. Suppose J modes are selected. For $J > 2$ a Cayley transformation (1) is used to construct a $J \times J$ orthogonal rotation matrix, R_u, from a skew-symmetric matrix, A, such that $R_u = (I - A)(I + A)^{-1}$, with $A = A_o(M - M^T)$. Here, M is a $J \times J$ matrix where all elements are populated with a uniform random number on $(0, 1)$, I is a $J \times J$ identity matrix, and A_o is an amplitude that adapts to the number of unproductive spins. A failure rate, K_f, is defined as the running average for the number of unproductive spins divided by the number of spins performed within a sweep. Setting $A_o = \frac{K_f}{2}$ generates small perturbations that avoid singularities in the Cayley transformation. When $A_o = \frac{1}{2}$, an average deviation angle of 5 degrees forms between the final and initial directions of the J basis vectors.

Importance sampling reduces the frequency of directed orthogonal rotations (DOR) applied to u-modes in competitive learning. However, randomly changing the directions of low-quality u-modes generates *creativity* as successive UOR produce a random walk in basis vector directions. This yields diffusive exploration within the undetermined subspace *without judgement*. Random noise enables the crossing of barriers in perception. This uncovers unexpected d-modes and i-modes, and increases tilt angles between the current and previous mode pair planes. A transduction of creativity to perception takes place by the RANU as unstructured latent information is extracted from u-modes with the aid of UOR. Together with DOR, u-modes are driven to pass the decision triad filter.

Avoiding data preprocessing and hyperparameters. The input data does not generally need to be preprocessed because ratios of variables are used to compare systems, with the mean and variance along a mode direction having statistical consistency in practice. To prevent numerical overflows, saturation limits are applied to variance ratios, the scoring function, and clustering quality factors. For a generic variable x, the transformation $x' = x/\sqrt{(1 + (x/c)^2)}$ bounds x' between $(-c, c)$ where c is a constant. Here, $c = 10^{12}$ for a variance ratio, $c = 100$ for the scoring function, and $c = 10$ for the clustering quality factor. In the limit $x/c << 1$, note that $x' \approx x$, which occurs most frequently. The saturation limits maintain stable calculations with negligible impact on learning capacity. The observed numerical stability originates from derivative-free calculations, and rotations and inner products of unit vectors keeps propagation of numerical errors negligible.

Adaptive optimization mitigates the risk of using suboptimal parameters and removes the need for hyperparameters. Hyperparameters may replace adaptive data-driven settings such as the voting threshold, but using hyperparameters in ML tends to overfit to training data, while increasing training times. Variations in parameters appearing in heuristic formulas shift dynamical equilibrium characteristics such as spin success rate and spin skip rate, however, SPLOC performance is insensitive to these changes due to adaptive mechanisms.

Discovery likelihood. To quantify functional and nonfunctional characteristics within the training set for the k-th d-mode, a set of univariate probability density functions (PDFs) are calculated (2) as $f_F(x_k|k)$ and $f_N(x_k|k)$ respectively. Here, x_k is a random variable characterizing a projection in the k-th mode, $\forall\, k$, ranging from 1 to D_d. The $(N_F + N_N) \times D_d$ different probability densities quantify key factors necessary for a system to function in a comparative context. In a molecular design scenario, consider N_U simulated systems proposed to be functional. The data from these simulations are projected into d-modes to yield $N_U \times D_d$ probability densities given by $f_U(x_k|k)$.

Let $I_{21} = 1 - \frac{1}{2}\int_{-\infty}^{\infty} |f_2(x) - f_1(x)|\, dx$ define the overlap between a pair of PDFs. An overlap (e.g. $0 \le I \le 1$) is mapped to the likelihood that two PDFs are distinguishable using the heuristic sigmoid-like function given by

$$P_d(I) = \left[\frac{1}{1 + \exp[24.333(I - 0.561)]}\right]^{\min\left(1, \sqrt{I/0.561}\right)} \quad [11]$$

where $P_d(I) \to 0$ faster than a sigmoid function when $I \to 1$. An alternative parametric version of the classifier has also been implemented that relies only on the first and second moments of the distribution. When classifying by moments, the overlap integral is calculated in the same way as defined above, except the functions $f_1(x)$ and $f_2(x)$ are assumed to be Gaussian functions. Since the k-th mode directly gives the mean, μ_k, and STD, σ_k, the integral for overlaps are precalculated to construct look-up tables. This makes CPU-time for classification by moments virtually instantaneous compared to the time to construct the data-packets. In practice, when applied to molecular dynamics simulation trajectories, the moment-classification results are frequently similar to the results obtained by employing univariate density estimation.

For convenience, let F, N, and U denote indices for functional, nonfunctional and unlabeled data streams respectively. The probability that system F is distinct from all nonfunctional systems with respect to mode k is given by $w(F, k) = \prod_N P_d(I_{FN})$. For mode k, the F data stream is distinctly different from all known nonfunctional systems when $w(F, k) \to 1$. Let

$t(U,k|F) = P_d(I_{UF})$. Here, $t(U,k|F)$ is the conditional probability for U to share a likeness to F in mode k, given that F is functional. Then $p(U,k) = 1 - \prod_F [1 - t(U,k|F)w(F,k)]$ is the probability that for mode k, the unknown system U is functional with respect to all known functional systems. When the unknown system has an identical match to a *single* functional system in mode k, then $p(U,k) = 1$. A similar procedure is applied to w' and t', being counterparts to w and t, with $F \to N$. For mode k, the probability that the unknown system U is unlike any of the known examples of nonfunctional systems is given by $q(U,k) = \prod_N [1 - t'(U,k|N)w'(N,k)]$.

The product, $p(U,k)\, q(U,k)$ provides a good baseline model for the likelihood for system U to be functional and not nonfunctional with respect to the k-th mode. Small $q(U,k)$ implies small $p(U,k)$. When $q(U,k) \to 1$, then $p(U,k)$ can range on $[0,1]$. In general, not all differences between a pair of F and N systems are relevant to function. Notice that the naive model of having $P_F(k) = p(U,k)\, q(U,k)$ or more simply, pq, severely downgrades the likelihood that an unknown system will function if it is different from all known functional and nonfunctional examples. Therefore, saturation levels are added to obtain a discovery likelihood (DL) that biases exploring the unknown. Letting $p \to \bar{q}p + q$ and $p \to \bar{p}q + p$ leads to the current model that $P_F(k) = [\bar{q}p + q]\,[\bar{p}q + p] = (1 - \bar{p}\bar{q})^2$.

With saturation levels included, the following desirable properties are obtained. System U with (many, few) characteristics similar to known F systems will have a relatively (high, low) DL. A low DL will result when many characteristics of the U system are similar to known N systems. When characteristics of system U differ from those from systems F and N, DL $\to 1$ because it is prudent to search unseen examples to discern the relevant characteristics that support function. There is also symmetry in the quantity $P_F(k) = [1 - \bar{p}(U,k)\,\bar{q}(U,k)]^2$.

The root mean square average of $P_F(k)$ over all d-modes gives an estimate for the DL for system U, denoted as $P_F(U)$. Propagation of error due to uncertainties in overlap integrals is calculated by adding Gaussian noise to the overlap integrals which creates variation in $P_F(U)$. The average over 626 trials gives the final DL, reported as $\langle P_F \rangle$ with its uncertainty ΔP_F.

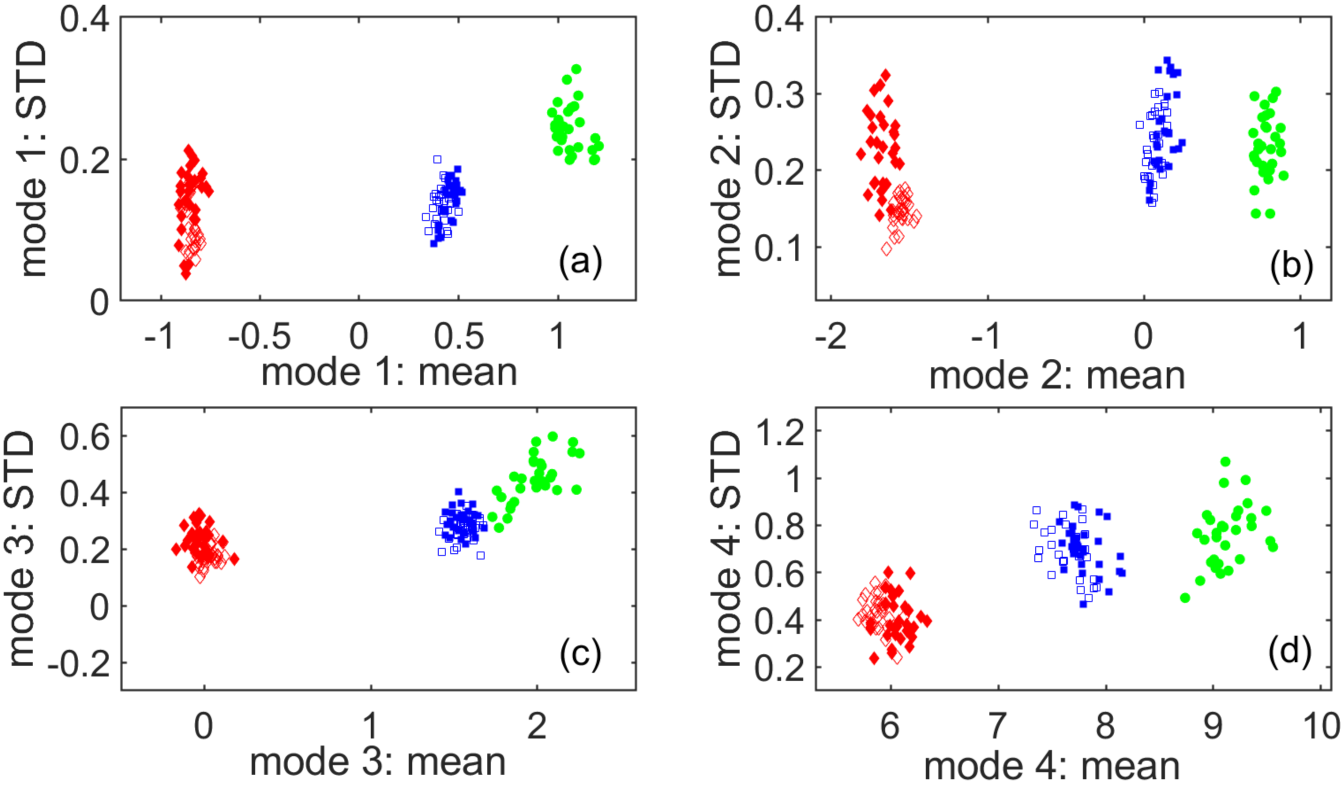

Fig. S1. The mode feature space plane (MFSP) is shown for all 4 d-modes that were extracted from the iris dataset. Training data contains Setosa (open red diamonds) and Virginica (open blue squares) while filled red diamonds and filled blue squares represent unseen Setosa and Virginica data respectively. Versicolor (filled green circles) was not trained on. Versicolor shares similar characteristic properties with Virginica for mode 3. The discriminant subspace provides a multivariate description of the differences between functional and nonfunctional systems that were trained on.

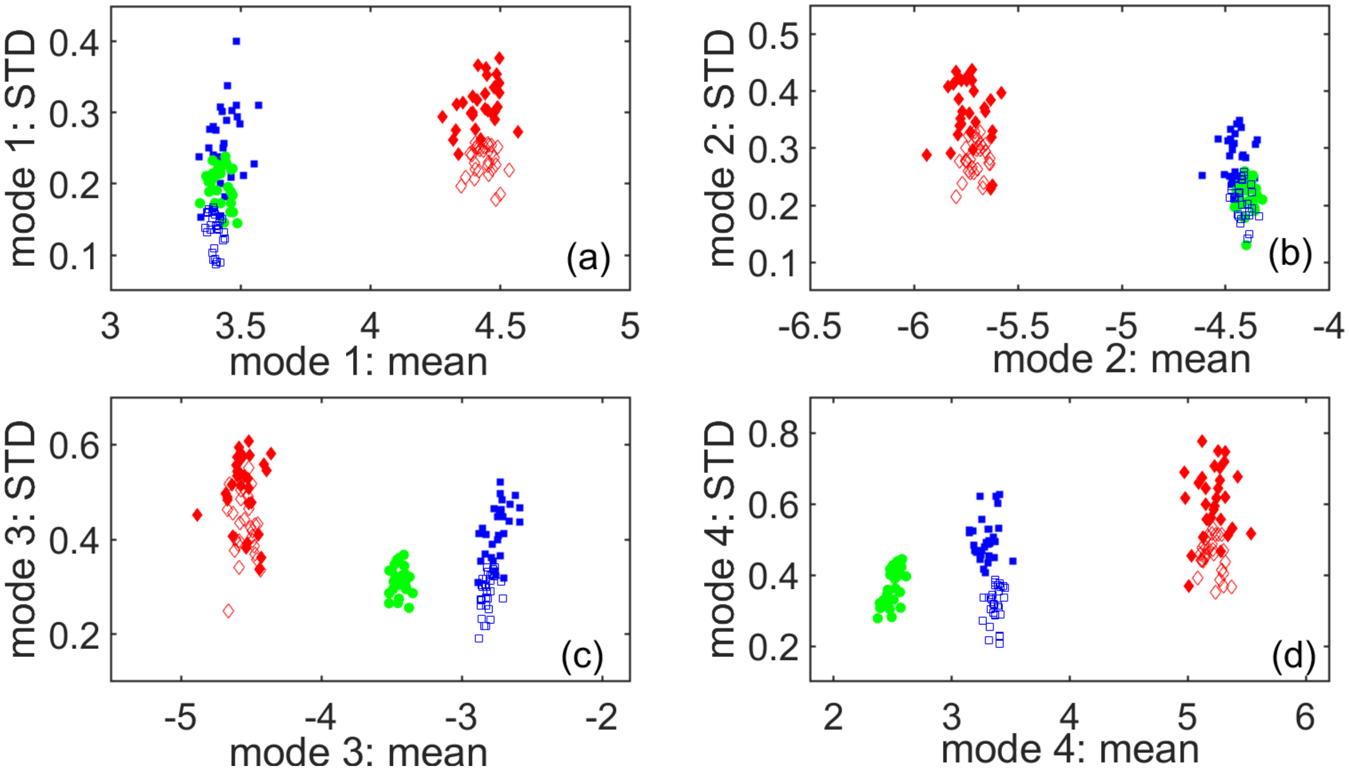

Fig. S2. The mode feature space plane (MFSP) is shown for the first 4 d-modes that were extracted from the wine dataset. Training data contains wine 1 (open red diamonds) and wine 2 (open blue squares) while filled red diamonds and filled blue squares represent unseen wine 1 and wine 2 data respectively. Wine 3 (filled green circles) was not trained on. Wine 3 shares the same characteristic properties with wine 2 for modes 1 and 2. The discriminant subspace gives a multivariate description of the differences between functional and nonfunctional systems that were trained on.

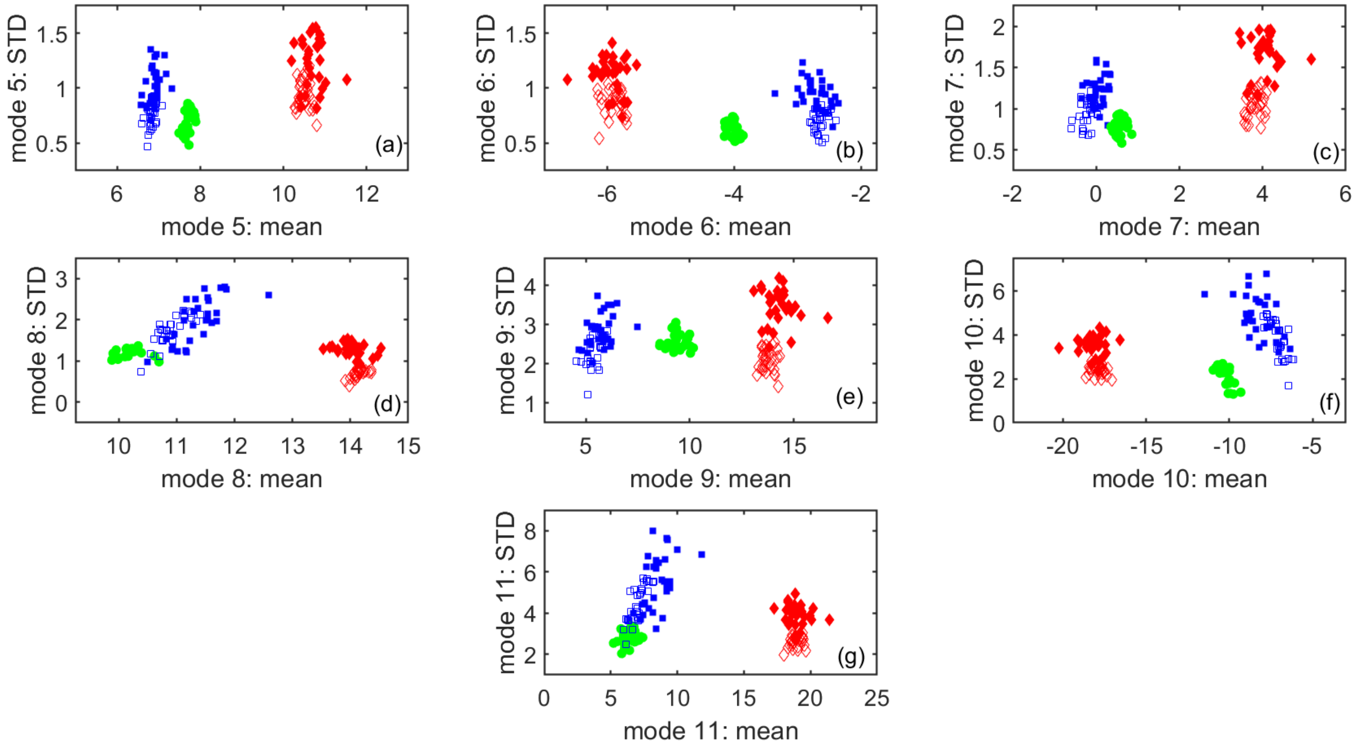

Fig. S3. Out of 11 d-modes, the mode feature space plane (MFSP) is shown for the remaining 7 d-modes that were extracted for the wine dataset. Training data contains wine 1 (open red diamonds) and wine 2 (open blue squares) while the filled red diamonds and filled blue squares represent unseen wine 1 and wine 2 data respectively. Wine 3 (filled green circles) was not trained on. Wine 3 shares similar characteristic properties with wine 2 for modes 5, 7, 8 and 11. The discriminant subspace provides a multivariate description of the differences between functional and nonfunctional systems that were trained on.

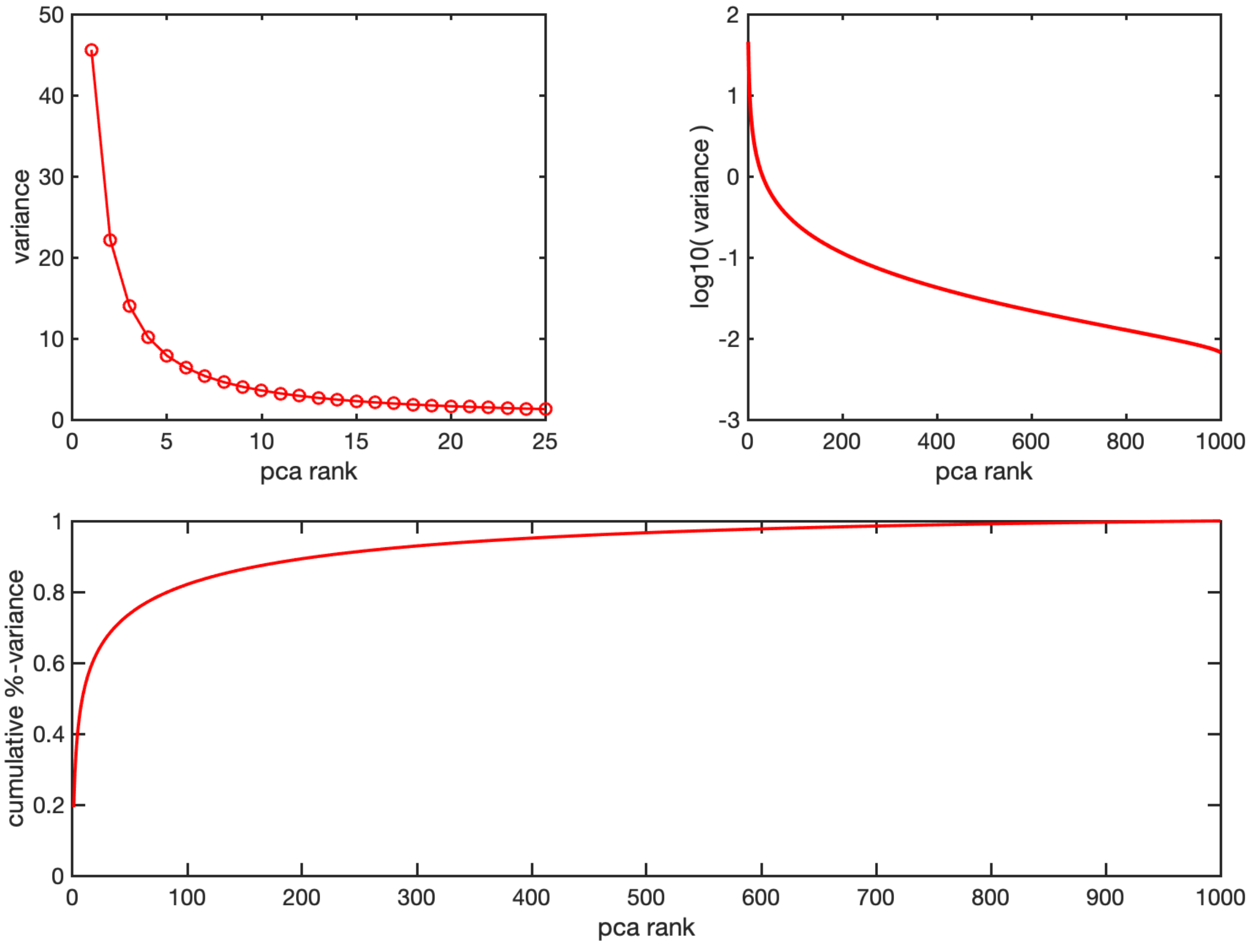

Fig. S4. PCA spectrum characteristics of the correlated Gaussian Noise (CGN) model for a system of $p = 1000$ variables. (a) The spectrum in variance highlights the scree characteristic that qualitatively arises from essential dynamics found in globular proteins. (b) The spectrum for all 1000 PCA modes is shown on a logarithmic scale. (c) Cumulative percentage of variance as a function of the number of PCA-modes.

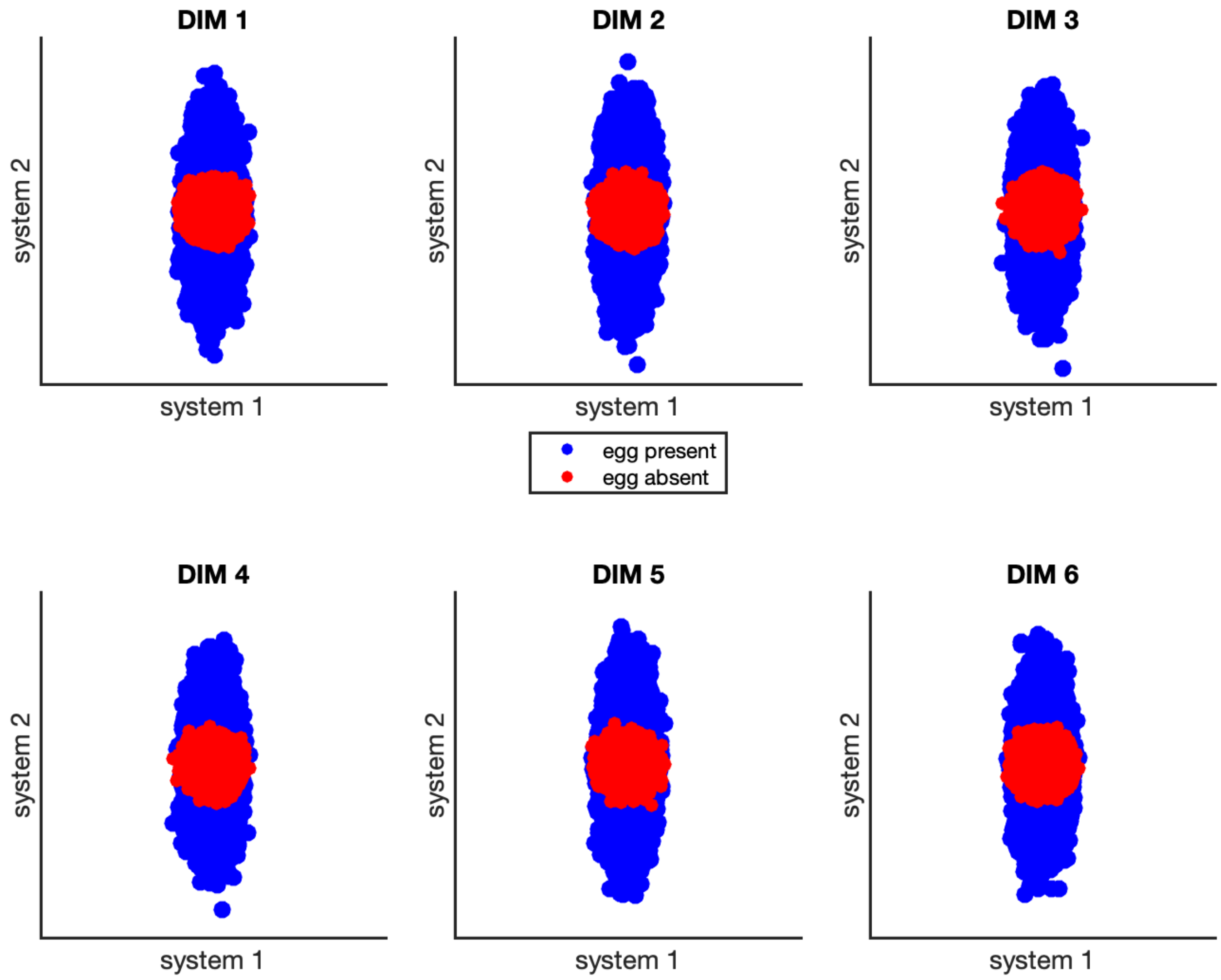

Fig. S5. Examples for $p = 500$ and 100 OPV. A large 2D egg is placed and scrambled within a 6D subspace subjected to correlated Gaussian noise (CGN). The data matrix is projected into the standard coordinates for systems 1 and 2. System 1 does not have an egg. System 2 can be absent of an egg (red) or hides an egg (blue). The circular red scatter shows that the projections of systems 1 and 2 are statistically equivalent in the absence of an egg. The elliptical blue scatter shows a large egg is readily detectable in all 6 dimensions.

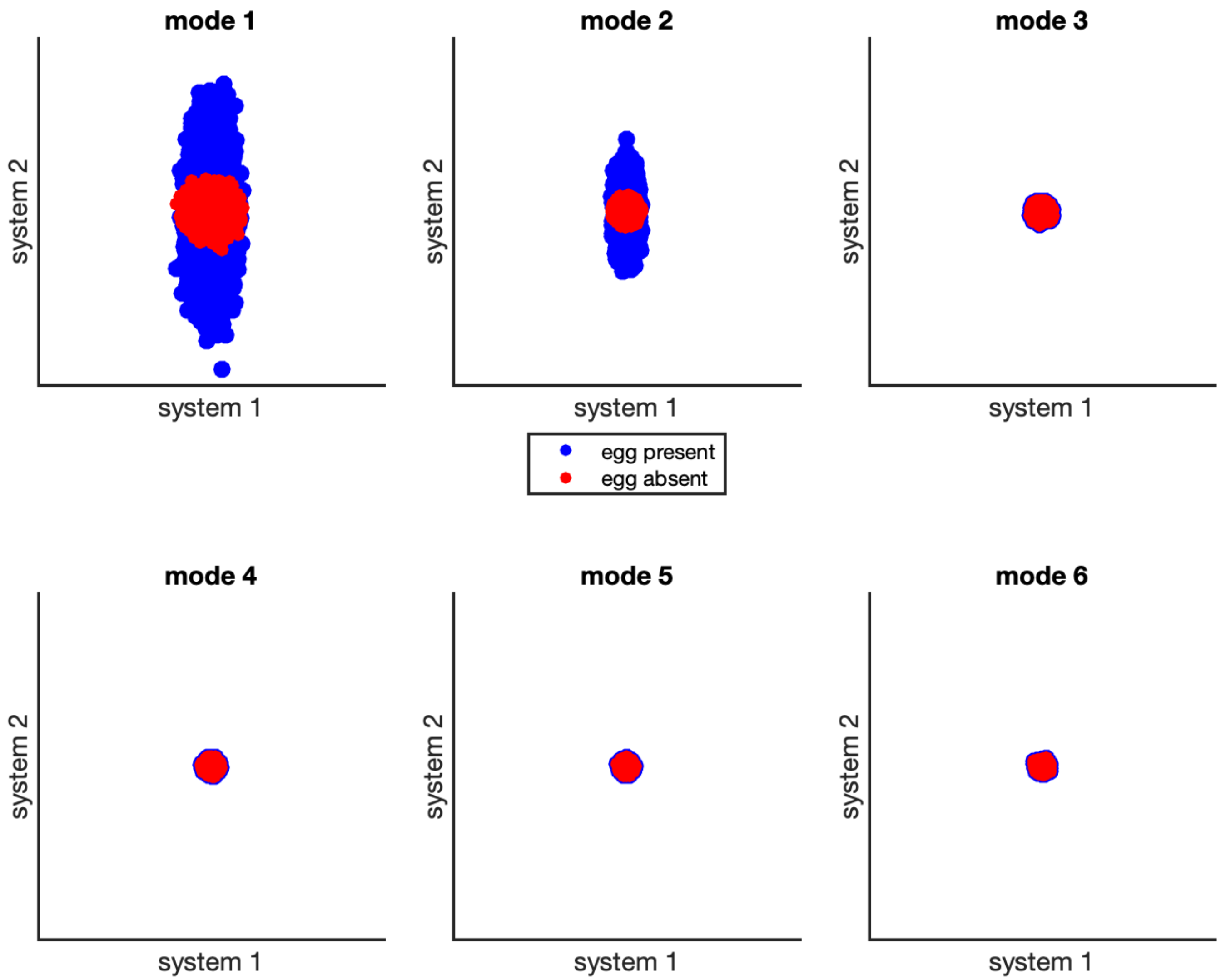

Fig. S6. The same large 2D egg that is placed in the 6D subspace shown in S7 is shown here in terms of the PCA eigenvector directions (modes) that characterize the covariance in the 6D subspace. Modes 1 to 6 label the rank-ordered PCA modes from highest to lowest variance. By construction, all modes, except for the 2 modes with the greatest variances, are not perturbed. This means the egg (i.e. the embedded signal) is fully described by two basis vectors. However, the egg is scrambled with respect to the original coordinates within the 6D space. The variables within the 6D subspace have correlations to variables outside of the 6D subspace for the CGN case, although these extended correlations are not present within a SGN concealing environment.

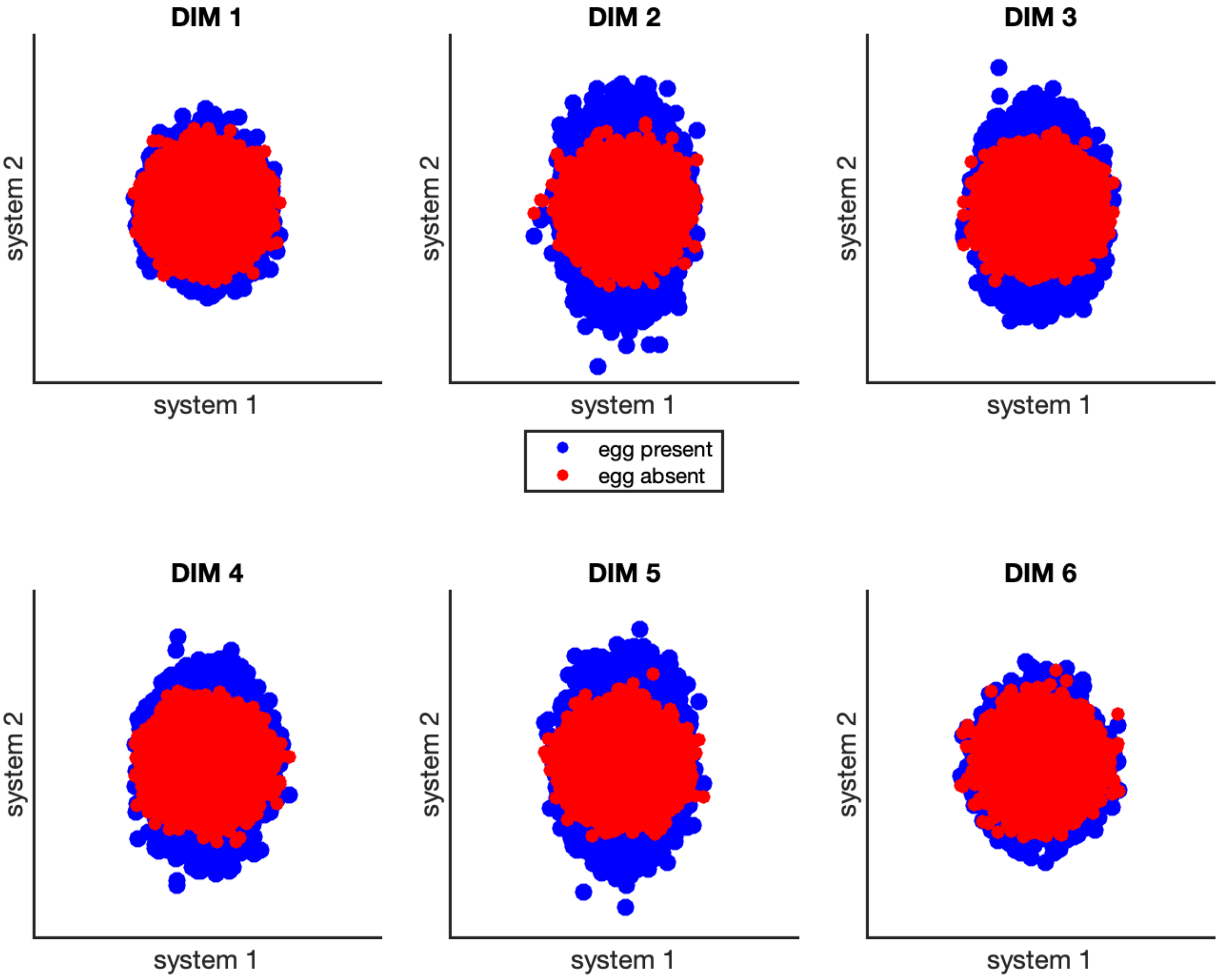

Fig. S7. A small 2D egg is placed within a 6D subspace subjected to correlated Gaussian noise (CGN) as described in Fig. S7. Despite showing the scatter plots on a different scale causing the scattering of points in this figure to look larger than what appears in Fig. S7, system 1 projections are identical between Figs. S6 and S8. Using the size of the circular red scatter as a reference, when a small egg is placed in system 2 it is difficult to detect because there is a near perfect overlap in the clusters. As such, red scatter almost covers up the blue scatter.

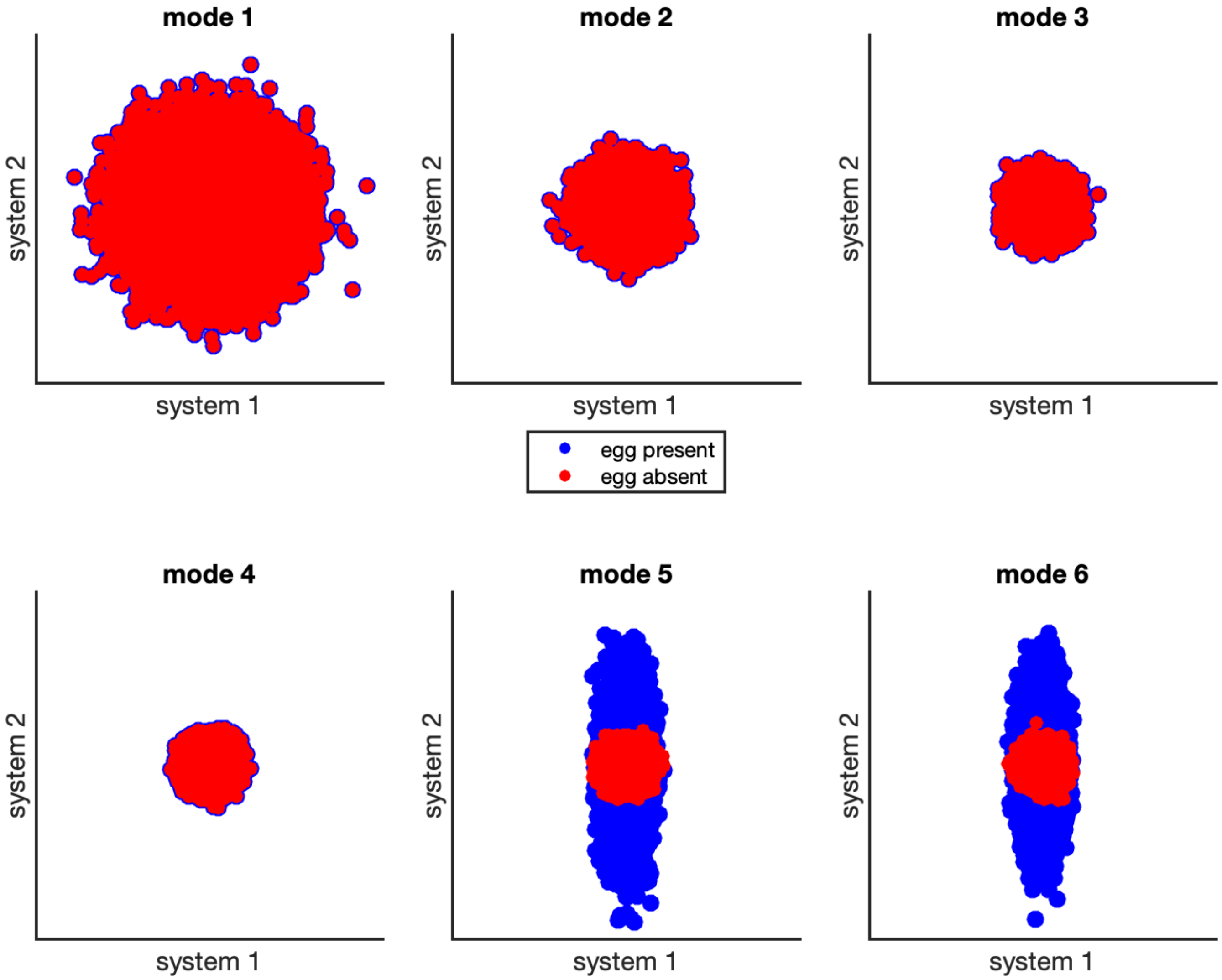

Fig. S8. The same small 2D egg is placed in the 6D subspace shown here in terms of the PCA eigenvector directions (modes) that characterize the covariance. The PCA-modes are identical to those in Fig. S7. By construction, all modes except for the 2 with smallest variance are not perturbed. This means the egg (i.e. the embedded signal) is fully described by two basis vectors. However, the egg is scrambled with respect to the original coordinates within the 6D space. The variables within the 6D subspace have correlations to variables outside of the 6D subspace for the CGN case, although these extended correlations are not present within a SGN concealing environment.

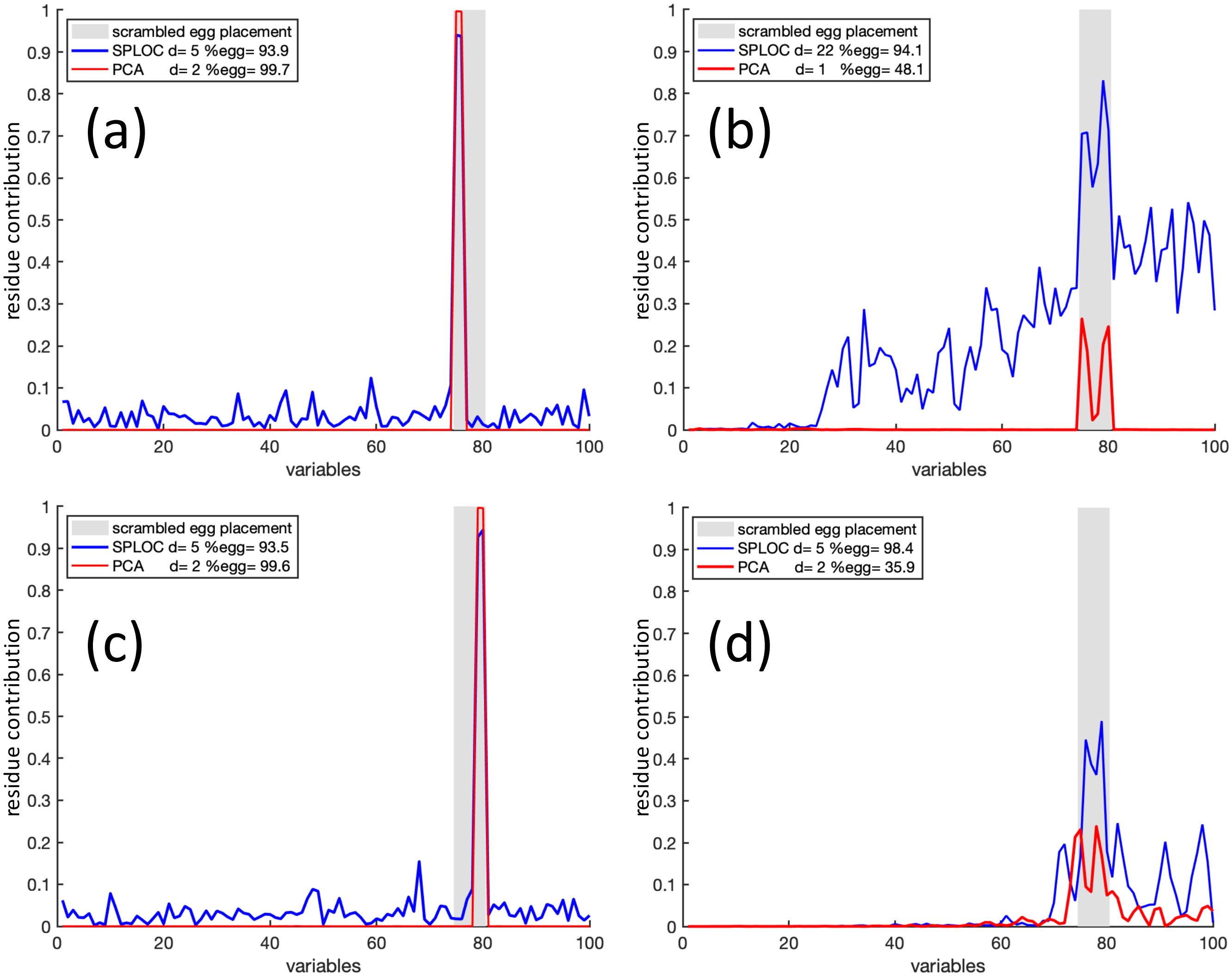

Fig. S9. Four representative egg hunt trials in 100 dimensions. Based on the sum of the squares over all d-modes, the residue contributions are compared using PCA and SPLOC for dimension reduction. Note that the total area under the curves is equal to the number of d-modes extracted during the egg hunt. The four cases constitute the SGN and CGN concealing environments that hide a large and small egg. (a) SGN and large egg. (b) CGN and large egg. (c) SGN and small egg. (d) CGN and small egg. In each panel, the legend summarizes the discriminant subspace dimension, d, and how much the initial scrambled egg is reconstructed.

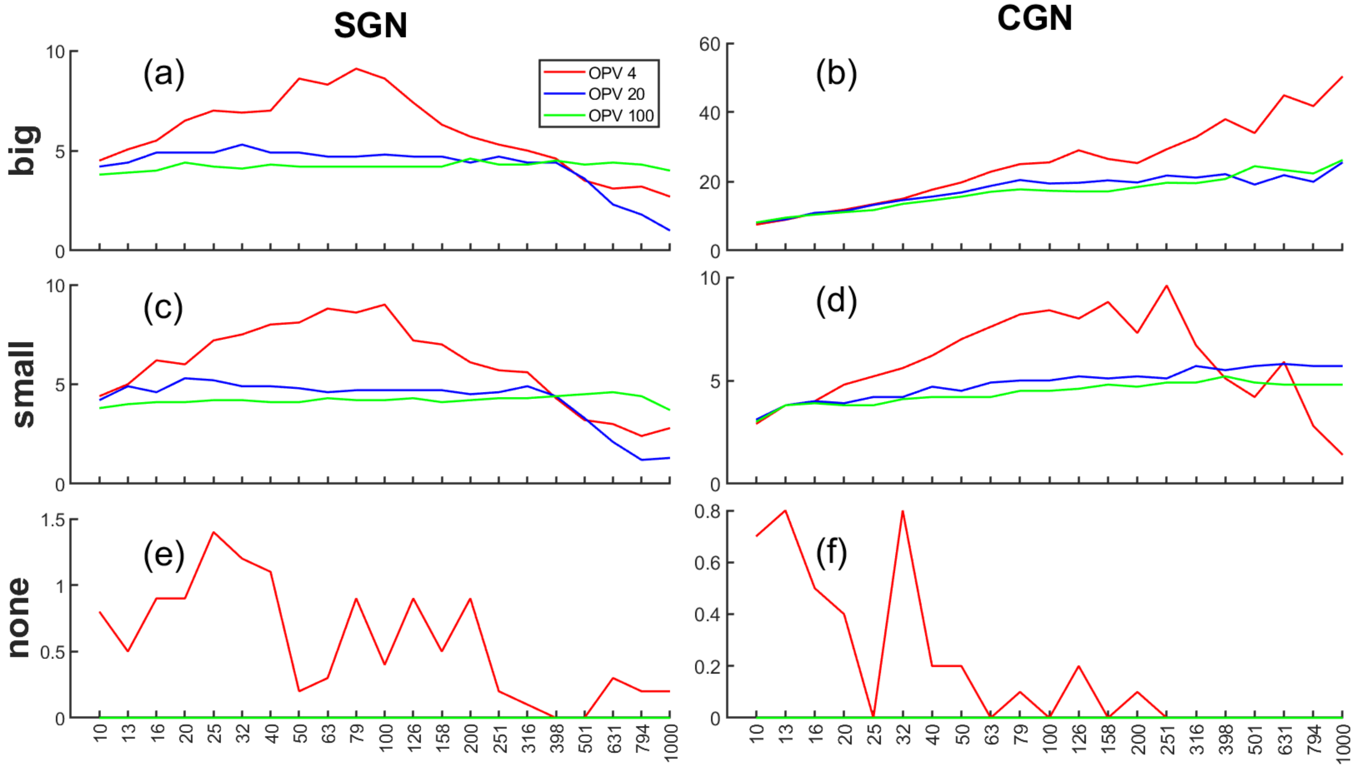

Fig. S10. SPLOC results for the number of d-modes extracted in 12 distinct egg hunts, and the control. This data represents an average over 10 trials made per egg hunt case. For the control, the greatest number of d-modes found was 3 for an OPV of 4.

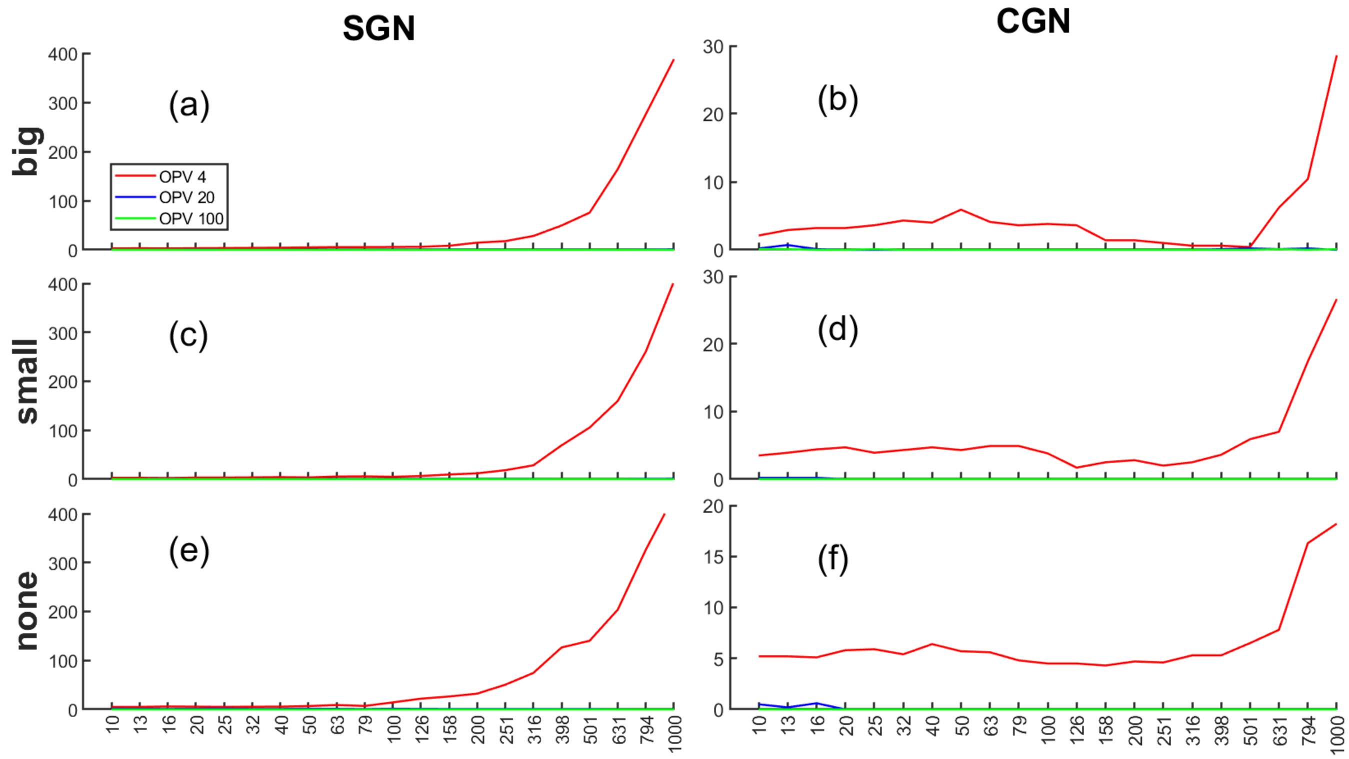

Fig. S11. SPLOC results for the number of u-modes extracted in 12 distinct egg hunts, and the control. This data represents an average over 10 trials made per egg hunt case.

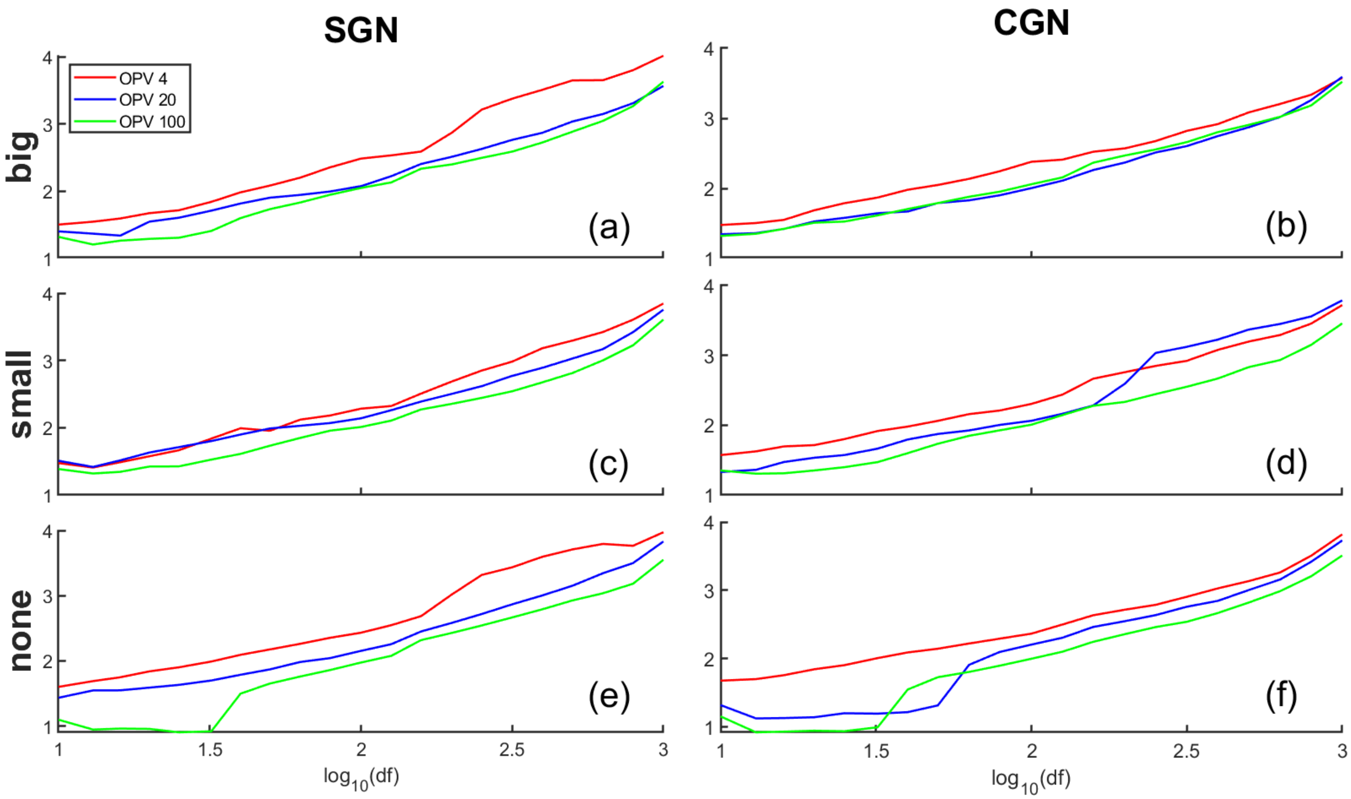

Fig. S12. SPLOC CPU-time characteristics for 12 distinct egg hunts, and the control. The CPU-time reflects the mean time for a single trial, where the average is taken over 10 trials. The vertical axis is $\log_{10}(\text{mean CPU seconds over 10 trials})$. On a high performance cluster using 16 processors, wall time for large systems is typically 4 to 8 times faster due to internal MATLAB threading with certain functions.

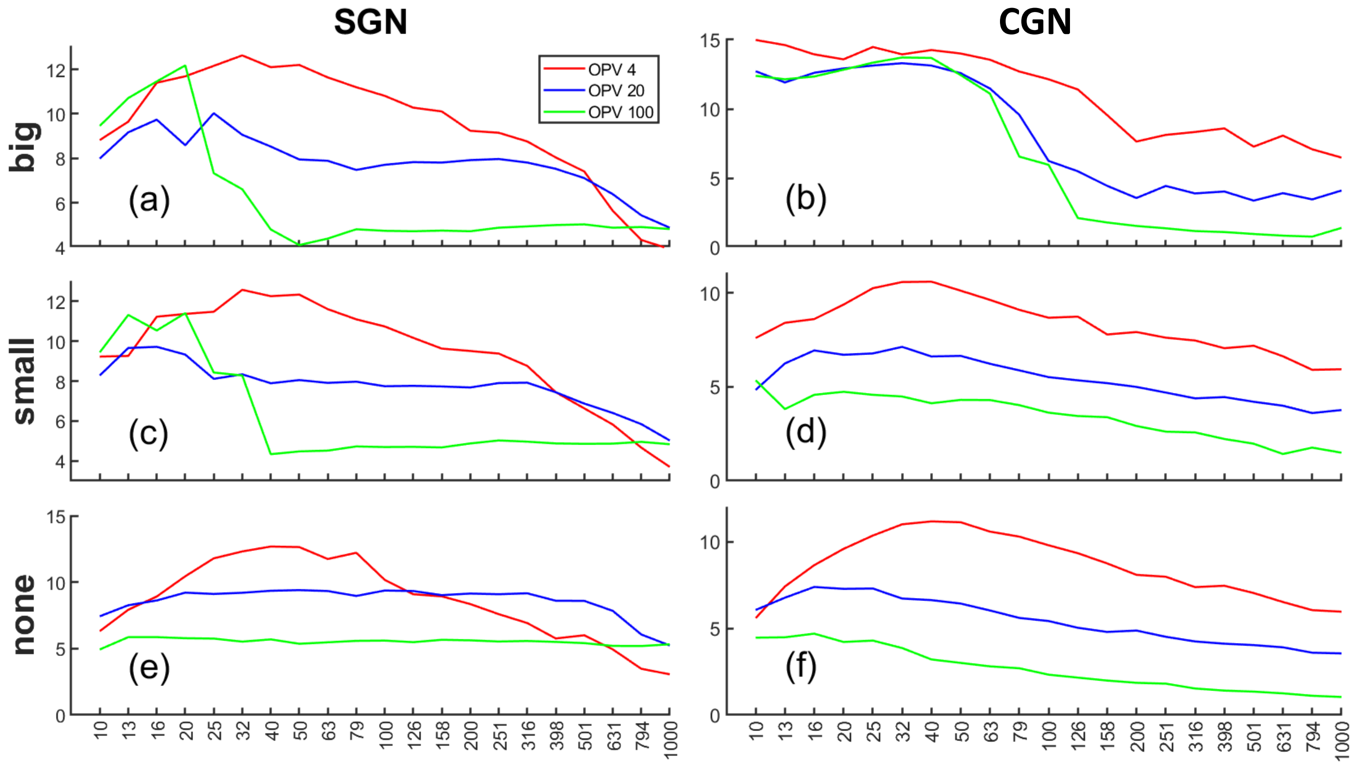

Fig. S13. $\triangle$EPM versus p is plotted. Here, $\triangle$EPM is the increase in efficacy per mode (EPM) relative to the baseline EPM from the initial basis set. Specifically, the increase in EPM is given by the EPM of the final basis set minus the EPM of the initial basis set that is generated from PCA. These results show that the process of spinning basis vectors increases the efficacy of the perceptron network, where the objective function being maximized is defined by the rectifying adaptive nonlinear unit (RANU) described in the text.

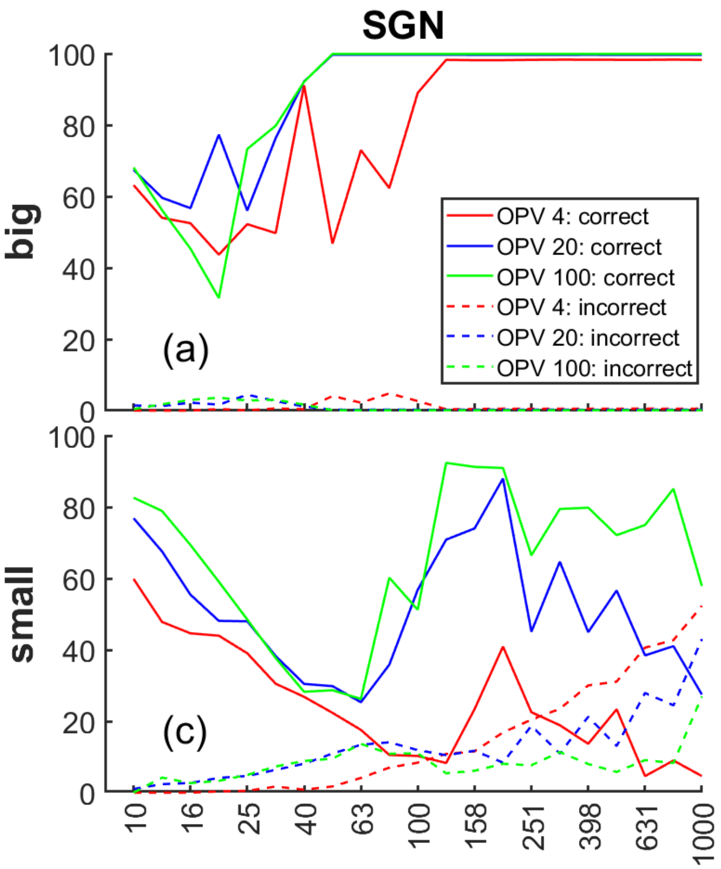

Fig. S14. Egg reconstruction using the initial basis set (IBS). As a technical note, the normal PCA protocol is not used here. The top PCA-modes, based on variance only, will generally not find the egg. For the results shown here, PCA is used only to generate a complete orthonormal basis set. The mode indexing is based on the decision triad to find the particular eigenvectors of the covariance matrix that are effective in reconstructing the egg. Lastly, the covariance matrix is one of three pooling types as explained in the main text. This data represents an average over 10 trials per egg hunt case. Essentially the same plot as Figure 4 (e-h) in the main text is shown here, except for the IBS. The small egg is more difficult for PCA to find compared to the large egg; as expected, the fluctuations are greater with the large egg. In general, the initial basis set has many u-modes.

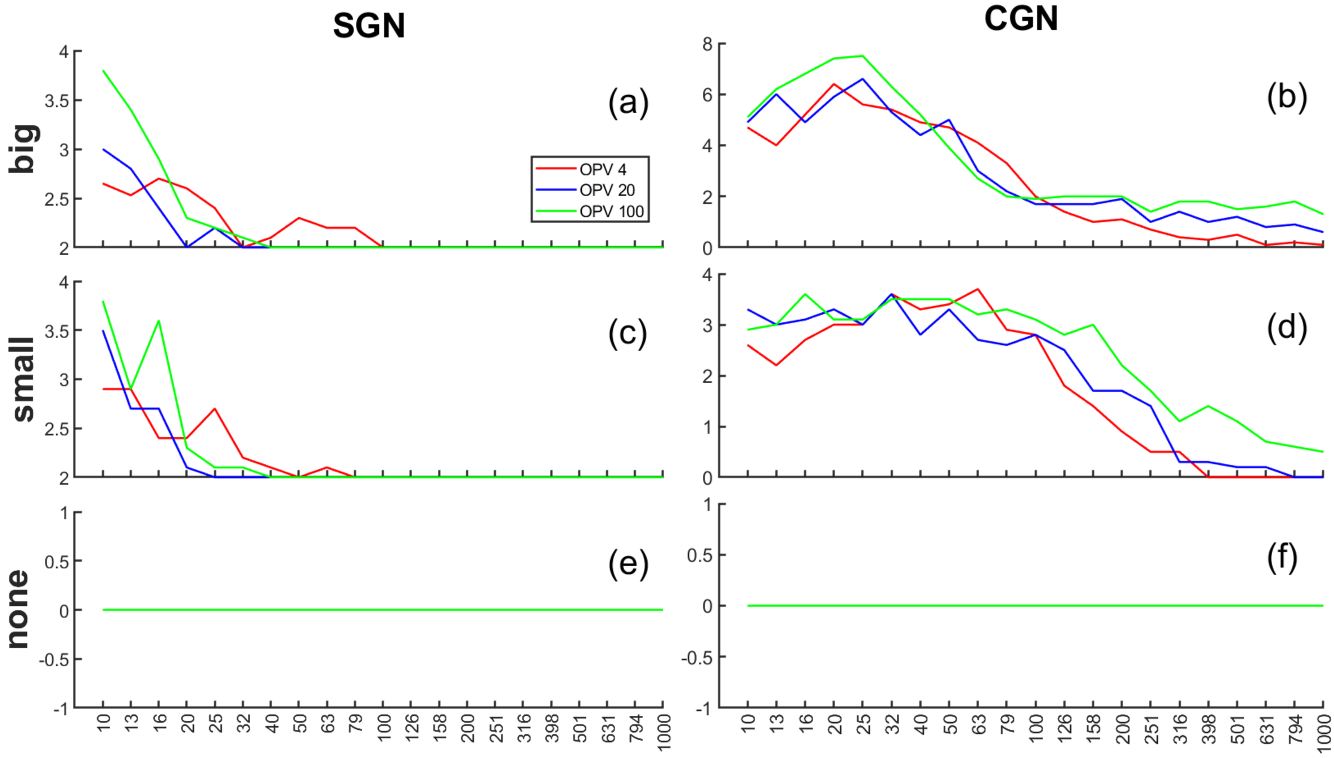

Fig. S15. Initial basis set results for the number of d-modes extracted in 12 distinct egg hunts, and for the control. This data represents an average over 10 trials per egg hunt case. As a technical note, the normal PCA protocol is not used here. The top PCA-modes, based on variance only, will generally not find the egg. For the results shown here, PCA is used only to generate a complete orthonormal basis set. The mode indexing is based on the decision triad to find the particular eigenvectors of the covariance matrix that are effective in reconstructing the egg. Lastly, the covariance matrix is one of three pooling types as explained in the main text.

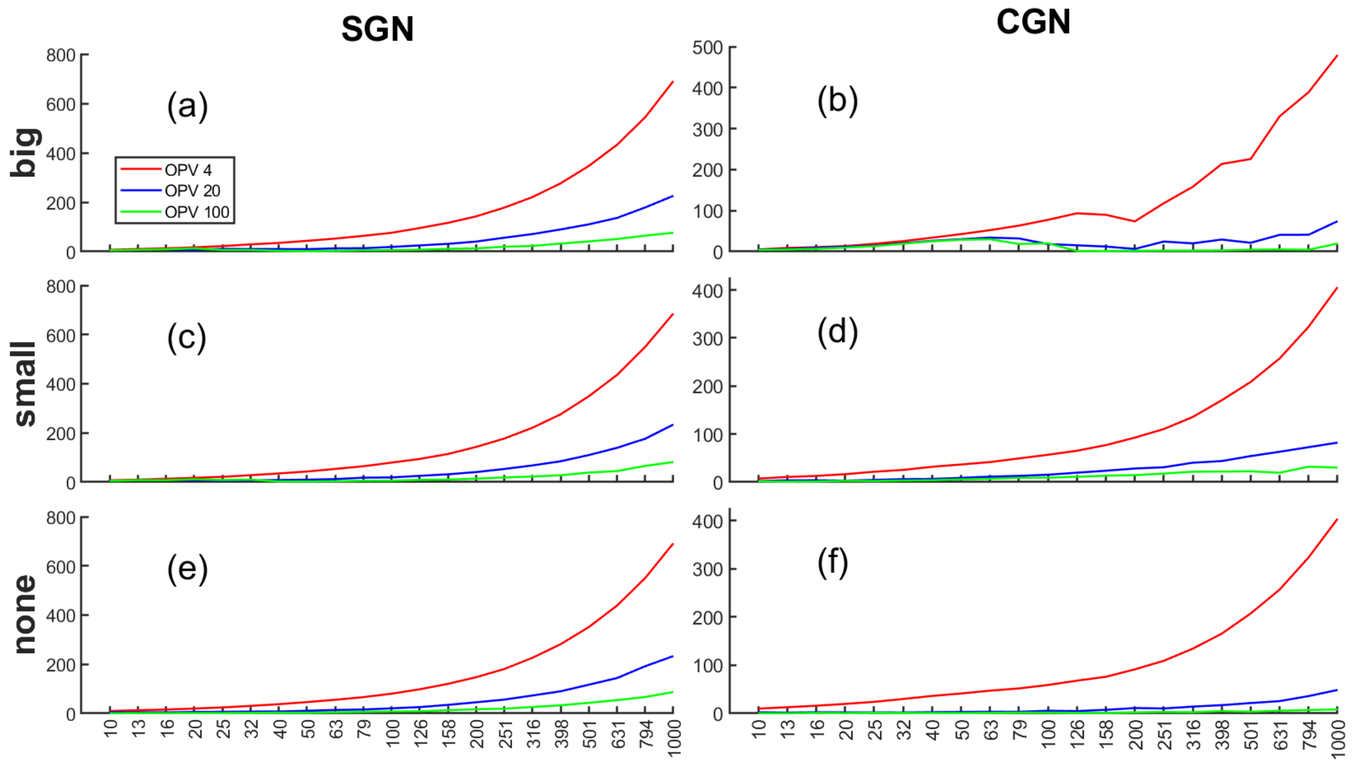

Fig. S16. Initial basis set results for the number of u-modes extracted in 12 distinct egg hunts, and for the control. This data represents an average over 10 trials per egg hunt case. As a technical note, the normal PCA protocol is not used here. The top PCA-modes, based on variance only, will generally not find the egg. For the results shown here, PCA is used only to generate a complete orthonormal basis set. The mode indexing is based on the decision triad to find the particular eigenvectors of the covariance matrix that are effective in reconstructing the egg. Lastly, the covariance matrix is one of three pooling types as explained in the main text.

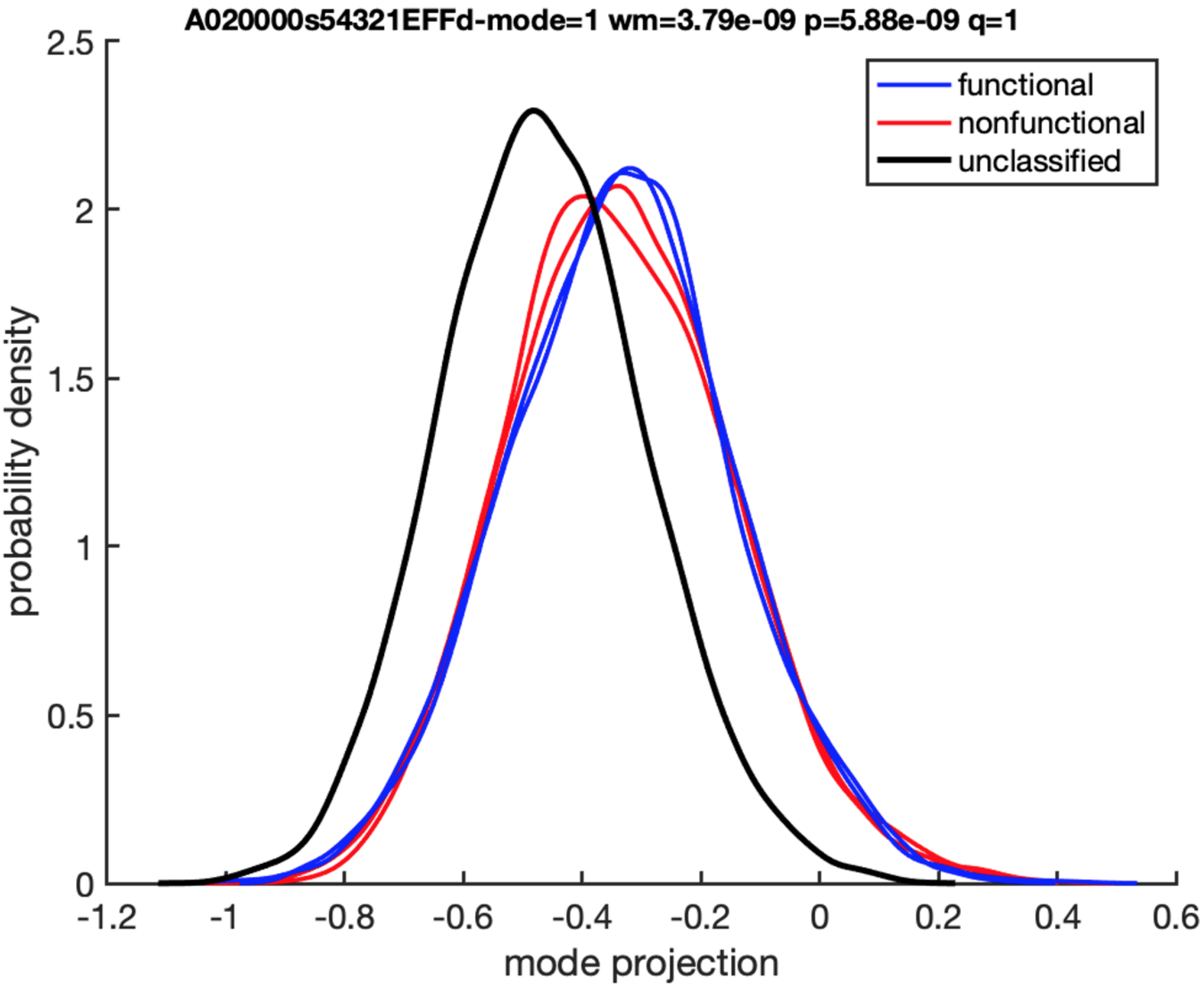

Fig. S17. Output from SPLOC showing that for the top PCA mode no discrimination is found between the functional and nonfunctional classes. The unclassified system in this example is the EFF synthetic molecule (shown in title). The EFF molecule does not look similar to either the functional (FLF or FLL) or the nonfunctional (FFF, FFL) molecules relative to the differences observed between functional and nonfunctional. Hence, the discovery likelihood becomes 1. Experimentally checking if this unknown molecule will function promotes learning, either because the prediction is correct, or because a wrong prediction will differentiate functional and nonfunctional molecules. The model developed by SPLOC is strengthened either because a novel functional molecule will be found, or because a wrong prediction will narrow the working hypothesis to better hone in on the characteristics that are critical to support function. In general, modes from PCA or PLS do not satisfy the decision triad, and are thus untrustworthy.

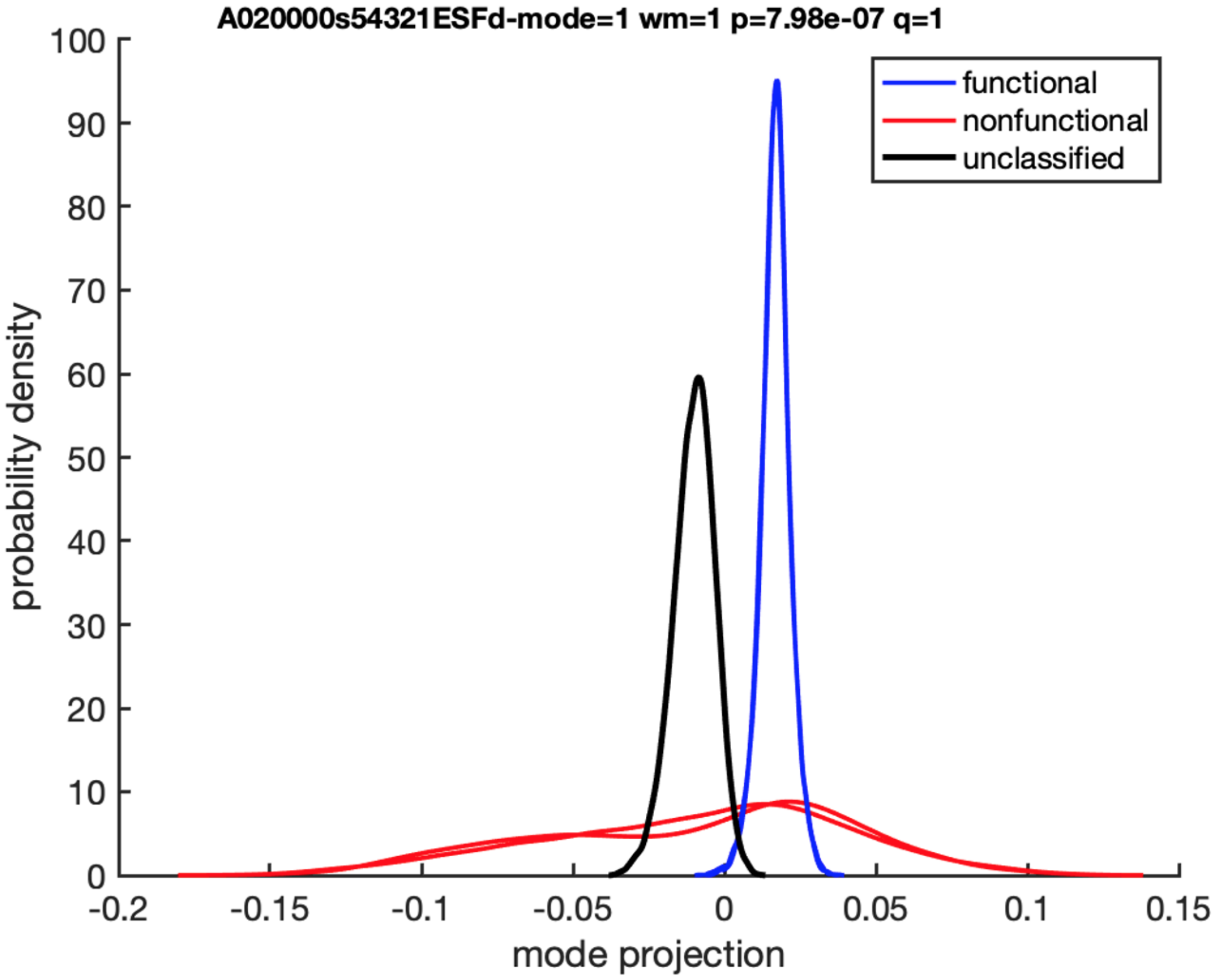

Fig. S18. Output from SPLOC showing that for the top DR SPLOC mode there is definitive differences between functional and nonfunctional classes. The unclassified molecule ESF (shown in title) does not share properties of functional or nonfunctional molecules. Hence, the discovery likelihood approaches 1 because performing an experiment to check if the unknown molecule will function promotes discovery, because a novel functional molecule will be found, or because a wrong prediction will narrow the working hypothesis to better hone in on the characteristics that are critical to support function.

Table S1. Four-fold cross-validation results are given for the dimension reduction (DR) methods: PCA, PLS and DR SPLOC. Training data was comprised of synthetic molecule trajectories with 500 and 20,000 frames (samples). For each DR method, three models are considered based on using 3, 8 and 13 features, defined as mode-projections in 3, 8 and 13 dimensions respectively. The average percent of correct predictions across the 4 validation tests is listed for each classification method. This average number is highlighted in three colors as: red $<$ 75% , 75% $\leq$ yellow $<$ 90% and green $\geq$ 90%, where (red, yellow, green) indicates (poor, good, excellent) results. To capture general trends, an average over all classifiers along with standard deviation (STD) is reported in the far right columns. The pattern in the table highlights greater classification accuracy is generally obtained with less DR. On average, the standard classifiers become less accurate when using more samples due to the aleatory nature of assigning each conformation to a specific class. Typically, DR SPLOC yields more reserved predictions for classifications by being less sensitive to the number of samples being classified.

**3 modes**	samples	LDA	QDA	GNB	LSVM	QSVM	RBSVM	**mean**	**STD**
PCA	500	94.00%	94.20%	91.50%	94.80%	96.50%	99.50%	95.08%	2.70%
	20k	57.90%	58.10%	58.00%	52.30%	51.60%	74.30%	58.70%	8.20%
PLS	500	98.10%	98.30%	87.50%	98.30%	99.60%	99.80%	96.93%	4.68%
	20k	73.50%	73.70%	60.60%	66.90%	52.70%	76.80%	67.37%	9.25%
DR SPLOC	500	87.40%	96.30%	94.20%	87.80%	95.40%	95.80%	92.82%	4.10%
	20k	70.20%	89.00%	88.60%	67.60%	88.50%	88.97%	82.15%	10.29%
**8 modes**	samples	LDA	QDA	GNB	LSVM	QSVM	RBFSVM	**mean**	**STD**
PCA	500	99.20%	100.00%	97.70%	99.30%	100.00%	100.00%	99.37%	0.90%
	20k	69.50%	73.00%	70.30%	69.90%	74.60%	99.23%	76.09%	11.51%
PLS	500	99.80%	99.90%	85.40%	99.60%	100.00%	100.00%	97.45%	5.91%
	20k	87.10%	88.40%	59.70%	87.40%	88.10%	99.31%	85.00%	13.24%
DR SPLOC	500	96.70%	99.80%	98.90%	98.90%	99.60%	99.50%	98.90%	1.14%
	20k	73.50%	90.70%	89.00%	74.50%	91.10%	91.17%	85.00%	8.56%
**13 modes**	samples	LDA	QDA	GNB	LSVM	QSVM	RBFSVM	**mean**	**STD**
PCA	500	99.20%	100.00%	98.20%	99.40%	100.00%	100.00%	99.47%	0.71%
	20k	77.30%	81.10%	78.00%	77.50%	82.60%	99.89%	82.73%	8.68%
PLS	500	100.00%	100.00%	85.40%	99.60%	100.00%	100.00%	97.50%	5.93%
	20k	89.00%	91.80%	59.20%	90.10%	91.20%	99.70%	86.83%	14.06%
DR SPLOC	500	99.50%	100.00%	99.60%	99.60%	100.00%	99.85%	99.76%	0.22%
	20k	89.10%	94.10%	93.30%	90.00%	95.30%	95.66%	92.91%	2.75%

Table S2. Classification results for 500 samples using 3 modes.
Table Description:
Header rows colored in tan highlight the training set, where the (upper, lower) header rows define (functional, nonfunctional) synthetic molecules. The rows just below the (upper, lower) header list all remaining (4,16) unseen synthetic molecules that are (functional, nonfunctional). The trailing light blue rows mark the end of the section and provide the mean and standard deviation of the data within the section. The PCA, PLS and DR SPLOC methods of dimension reduction (DR) are compared across three sections of the table. Six classifiers applied to the trajectories of unseen molecules yield the likelihood that a molecule is functional. The discovery likelihood (DL) is also listed. The mean and standard deviations for likelihood over the set of functional and nonfunctional molecules are reported. The second to last row reports the Cohen kappa statistic(3) relative to the exact know result that all molecules of the form aLc are functional, and the remainder are not. The last row is the difference in the functional mean minus the nonfunctional mean, defining a gap. Gap values are color-coded for the standard classifiers: red when (gap < 0.3), yellow when (0.3 gap < 0.7), and green when (gap > 0.7), respectively indicating poor, good, and excellent predictions on average. The gap from DL is not color-coded because DL is not a classification likelihood. A (small, large) gap in DL gives (less, more) confidence to regard an unseen molecule as nonfunctional without an experimental test. An unseen molecule with characteristics that differ from known nonfunctional characteristics will have a high likelihood that an experimental test should be performed.

PCA	500 samples			3 modes			
	LDA	QDA	GNB	LSVM	QSVM	RBSVM	DL
FLL	0.912	0.938	0.926	0.924	0.932	0.994	0.796
FLF	0.984	0.928	0.878	0.960	0.998	1.000	0.994
FLT	1.000	1.000	1.000	1.000	1.000	1.000	0.997
ELL	1.000	1.000	1.000	1.000	1.000	1.000	0.853
ELT	1.000	1.000	1.000	1.000	1.000	1.000	0.816
ELF	1.000	1.000	1.000	1.000	1.000	0.684	0.816
mean	1.000	1.000	1.000	1.000	1.000	0.921	0.871
STD	0.000	0.000	0.000	0.000	0.000	0.158	0.086
FFL	0.056	0.080	0.128	0.060	0.062	0.004	0.409
FFF	0.076	0.010	0.000	0.028	0.010	0.000	0.415
FSL	0.602	0.668	0.658	0.624	0.590	0.534	0.528
FST	0.720	0.732	0.720	0.728	0.720	0.658	0.703
FSF	0.000	0.000	0.000	0.000	0.004	0.008	0.603
FTL	0.506	0.594	0.658	0.500	0.434	0.634	0.755
FTT	0.678	0.716	0.656	0.696	0.702	0.728	0.543
FTF	0.000	0.046	0.244	0.000	0.484	0.000	0.577
FFT	0.376	0.462	0.422	0.426	0.402	0.468	0.501
ESL	1.000	1.000	1.000	1.000	1.000	1.000	0.882
EST	1.000	1.000	1.000	1.000	1.000	1.000	0.996
ESF	1.000	1.000	1.000	1.000	1.000	1.000	1.000
ETL	1.000	1.000	1.000	1.000	1.000	1.000	0.832
ETT	1.000	1.000	1.000	1.000	1.000	0.990	0.835
ETF	0.932	0.986	1.000	0.876	0.996	0.632	0.817
EFL	1.000	1.000	1.000	1.000	1.000	1.000	0.994
EFT	1.000	1.000	1.000	1.000	1.000	1.000	0.826
EFF	0.842	0.922	0.976	0.810	0.710	0.308	0.816
mean	0.729	0.758	0.771	0.729	0.753	0.685	0.763
STD	0.349	0.336	0.316	0.343	0.303	0.350	0.170
kappa	0.161	0.161	0.161	0.200	0.200	0.200	0.059
gap	0.272	0.242	0.229	0.271	0.247	0.236	0.108

PLS	500 samples			3 modes			
	LDA	QDA	GNB	LSVM	QSVM	RBSVM	DL
FLL	1.000	0.998	1.000	0.992	1.000	1.000	0.990
FLF	0.994	0.960	0.748	0.972	0.998	1.000	0.895
FLT	1.000	1.000	1.000	1.000	1.000	1.000	0.992
ELL	1.000	1.000	1.000	1.000	1.000	1.000	0.993
ELT	1.000	1.000	1.000	1.000	1.000	1.000	0.914
ELF	0.844	0.974	0.858	0.946	1.000	0.908	0.737
mean	0.961	0.994	0.965	0.987	1.000	0.977	0.909
STD	0.078	0.013	0.071	0.027	0.000	0.046	0.120
FFL	0.002	0.000	0.256	0.002	0.000	0.000	0.560
FFF	0.070	0.026	0.000	0.036	0.008	0.004	0.384
FSL	0.694	0.492	0.902	0.650	0.562	0.628	0.954
FST	0.830	0.544	0.984	0.700	0.688	0.886	0.986
FSF	0.010	0.000	0.000	0.000	0.014	0.008	0.615
FTL	0.468	0.362	0.782	0.344	0.384	0.516	0.806
FTT	0.524	0.342	0.926	0.502	0.432	0.646	0.885
FTF	0.000	0.000	0.236	0.000	0.388	0.000	0.804
FFT	0.572	0.306	0.872	0.486	0.430	0.656	0.891
ESL	1.000	0.998	1.000	1.000	1.000	0.996	0.991
EST	1.000	1.000	1.000	1.000	1.000	1.000	0.922
ESF	0.908	0.958	0.992	0.936	1.000	0.982	0.994
ETL	0.998	0.912	1.000	0.996	0.972	0.880	0.998
ETT	0.994	0.748	1.000	1.000	0.978	0.728	0.733
ETF	0.334	0.420	0.960	0.366	0.964	0.554	0.804
EFL	0.962	0.724	1.000	0.956	0.912	0.848	0.981
EFT	1.000	0.974	1.000	1.000	0.992	0.876	0.951
EFF	0.360	0.254	0.948	0.364	0.476	0.270	0.792
mean	0.666	0.565	0.850	0.644	0.700	0.655	0.882
STD	0.353	0.346	0.295	0.359	0.317	0.323	0.112
kappa	0.241	0.385	0.125	0.286	0.286	0.161	0.029
gap	0.295	0.429	0.114	0.343	0.301	0.322	0.027

DR SPLOC	500 samples			3 modes			
	LDA	QDA	GNB	LSVM	QSVM	RBSVM	DL
FLL	1.000	0.998	0.998	0.990	0.996	0.994	1.000
FLF	0.968	0.966	0.972	0.966	0.990	0.976	1.000
FLT	0.696	0.372	0.774	0.584	0.562	0.564	0.996
ELL	0.928	0.692	0.730	0.882	0.816	0.774	0.998
ELT	0.338	0.364	0.724	0.248	0.472	0.500	0.998
ELF	0.978	0.050	0.410	0.982	0.654	0.540	0.965
mean	0.735	0.370	0.660	0.674	0.626	0.595	0.989
STD	0.292	0.262	0.168	0.331	0.147	0.123	0.016
FFL	0.032	0.004	0.060	0.020	0.012	0.010	0.000
FFF	0.430	0.118	0.144	0.430	0.152	0.118	0.000
FSL	0.000	0.000	0.000	0.000	0.000	0.000	0.999
FST	0.010	0.000	0.000	0.006	0.000	0.000	0.996
FSF	0.102	0.000	0.000	0.130	0.000	0.000	0.999
FTL	0.154	0.100	0.116	0.142	0.120	0.108	0.671
FTT	0.042	0.022	0.052	0.030	0.040	0.032	0.001
FTF	0.104	0.000	0.126	0.122	0.102	0.090	0.000
FFT	0.214	0.088	0.226	0.188	0.110	0.110	0.034
ESL	0.032	0.000	0.000	0.024	0.000	0.000	0.998
EST	0.002	0.000	0.000	0.002	0.000	0.000	1.000
ESF	0.026	0.000	0.000	0.024	0.000	0.000	0.999
ETL	0.052	0.000	0.000	0.044	0.000	0.000	0.402
ETT	0.000	0.000	0.098	0.000	0.000	0.004	0.459
ETF	0.000	0.000	0.034	0.002	0.016	0.012	0.581
EFL	0.458	0.226	0.326	0.384	0.288	0.264	0.003
EFT	0.010	0.010	0.052	0.008	0.014	0.020	0.001
EFF	0.090	0.030	0.036	0.086	0.042	0.034	0.000
mean	0.081	0.030	0.067	0.075	0.046	0.042	0.509
STD	0.118	0.061	0.094	0.102	0.078	0.071	0.447
kappa	0.882	0.600	0.882	0.882	0.882	0.882	0.385
gap	0.654	0.340	0.593	0.600	0.580	0.552	0.480

Table S3. Classification results for 500 samples using 8 modes.
Table Description: (same as Supplementary Table 2)
Header rows colored in tan highlight the training set, where the (upper, lower) header rows define (functional, nonfunctional) synthetic molecules. The rows just below the (upper, lower) header list all remaining (4,16) unseen synthetic molecules that are (functional, nonfunctional). The trailing light blue rows mark the end of the section and provide the mean and standard deviation of the data within the section. The PCA, PLS and DR SPLOC methods of dimension reduction (DR) are compared across three sections of the table. Six classifiers applied to the trajectories of unseen molecules yield the likelihood that a molecule is functional. The discovery likelihood (DL) is also listed. The mean and standard deviations for likelihood over the set of functional and nonfunctional molecules are reported. The mean and standard deviations for likelihood over the set of functional and nonfunctional molecules are reported. The second to last row reports the Cohen kappa statistic(3) relative to the exact know result that all molecules of the form aLc are functional, and the remainder are not. The last row is the difference in the functional mean minus the nonfunctional mean, defining a gap. Gap values are color-coded for the standard classifiers: red when (gap < 0.3), yellow when (0.3 gap < 0.7), and green when (gap > 0.7), respectively indicating poor, good, and excellent predictions on average. The gap from DL is not color-coded because DL is not a classification likelihood. A (small, large) gap in DL gives (less, more) confidence to regard an unseen molecule as nonfunctional without an experimental test. An unseen molecule with characteristics that differ from known nonfunctional characteristics will have a high likelihood that an experimental test should be performed.

PCA	500 samples		8 modes				
	LDA	QDA	GNB	LSVM	QSVM	RBSVM	DL
FLL	1.000	1.000	0.998	1.000	1.000	1.000	0.914
FLF	0.998	1.000	0.928	1.000	1.000	1.000	0.915
FLT	1.000	0.930	1.000	1.000	1.000	1.000	0.980
ELL	0.998	0.660	0.696	0.998	0.714	0.838	0.883
ELT	1.000	0.542	0.892	1.000	0.504	0.500	0.895
ELF	0.710	0.388	0.524	0.162	0.502	0.316	0.918
mean	0.927	0.630	0.778	0.790	0.680	0.664	0.919
STD	0.145	0.229	0.211	0.419	0.235	0.312	0.043
FFL	0.000	0.000	0.000	0.000	0.000	0.000	0.677
FFF	0.032	0.000	0.000	0.026	0.000	0.000	0.681
FSL	0.622	0.250	0.748	0.562	0.186	0.476	0.786
FST	0.610	0.214	0.428	0.596	0.266	0.402	0.830
FSF	0.046	0.042	0.016	0.032	0.084	0.066	0.804
FTL	0.274	0.236	0.334	0.344	0.304	0.274	0.824
FTT	0.494	0.070	0.490	0.550	0.246	0.194	0.798
FTF	0.000	0.022	0.220	0.000	0.248	0.054	0.767
FFT	0.514	0.220	0.456	0.436	0.362	0.524	0.775
ESL	0.764	0.162	0.370	0.912	0.258	0.456	0.942
EST	1.000	0.588	0.832	1.000	0.782	0.776	0.931
ESF	0.746	0.874	0.726	0.746	0.870	0.806	0.905
ETL	0.802	0.120	0.590	0.866	0.684	0.404	0.868
ETT	0.846	0.232	0.664	0.940	0.514	0.420	0.829
ETF	0.292	0.546	0.632	0.384	0.720	0.602	0.864
EFL	0.278	0.000	0.076	0.640	0.188	0.042	0.913
EFT	0.882	0.388	0.572	0.924	0.430	0.368	0.873
EFF	0.416	0.250	0.658	0.654	0.846	0.420	0.818
mean	0.537	0.263	0.488	0.599	0.437	0.393	0.845
STD	0.300	0.235	0.238	0.306	0.262	0.230	0.056
kappa	0.333	0.600	0.385	0.143	0.500	0.400	0.000
gap	0.390	0.367	0.290	0.191	0.243	0.271	0.074

PLS	500 samples		8 modes				
	LDA	QDA	GNB	LSVM	QSVM	RBSVM	DL
FLL	1.000	1.000	1.000	1.000	1.000	1.000	0.964
FLF	1.000	1.000	0.740	1.000	1.000	1.000	0.888
FLT	1.000	1.000	1.000	1.000	1.000	1.000	0.942
ELL	0.980	0.830	0.984	1.000	0.866	0.912	0.930
ELT	0.786	0.696	1.000	0.990	0.764	0.864	0.960
ELF	0.306	0.416	0.760	0.420	0.962	0.324	0.852
mean	0.768	0.736	0.936	0.853	0.898	0.775	0.921
STD	0.323	0.247	0.118	0.288	0.106	0.306	0.048
FFL	0.000	0.000	0.318	0.000	0.000	0.000	0.718
FFF	0.018	0.004	0.000	0.018	0.000	0.000	0.668
FSL	0.612	0.464	0.824	0.518	0.752	0.796	0.959
FST	0.238	0.250	0.810	0.304	0.766	0.642	0.929
FSF	0.042	0.000	0.000	0.014	0.000	0.090	0.798
FTL	0.396	0.270	0.774	0.360	0.292	0.344	0.876
FTT	0.552	0.378	0.856	0.536	0.234	0.180	0.936
FTF	0.010	0.006	0.290	0.048	0.416	0.002	0.809
FFT	0.636	0.340	0.600	0.592	0.430	0.452	0.923
ESL	0.656	0.382	1.000	0.892	0.842	0.876	0.991
EST	0.870	0.832	1.000	0.990	1.000	0.952	0.926
ESF	0.578	0.634	0.996	0.756	1.000	1.000	0.983
ETL	0.570	0.438	1.000	0.740	0.592	0.706	0.988
ETT	0.804	0.550	0.992	0.934	0.592	0.668	0.880
ETF	0.090	0.312	0.920	0.410	1.000	0.364	0.828
EFL	0.470	0.066	1.000	0.798	0.320	0.466	0.977
EFT	0.584	0.394	1.000	0.868	0.410	0.530	0.935
EFF	0.304	0.366	0.876	0.540	0.854	0.410	0.895
mean	0.463	0.355	0.809	0.581	0.594	0.530	0.915
STD	0.261	0.219	0.287	0.299	0.308	0.298	0.062
kappa	0.231	0.600	0.125	0.143	0.333	0.280	0.000
gap	0.305	0.380	0.127	0.271	0.304	0.245	0.006

DR SPLOC	500 samples		8 modes				
	LDA	QDA	GNB	LSVM	QSVM	RBSVM	DL
FLL	1.000	1.000	0.998	1.000	1.000	1.000	1.000
FLF	1.000	0.998	0.994	1.000	1.000	1.000	1.000
FLT	1.000	0.460	0.724	0.982	0.976	0.886	0.980
ELL	1.000	0.660	0.676	0.978	0.978	0.898	0.966
ELT	1.000	0.432	0.784	1.000	0.996	0.978	0.993
ELF	0.552	0.000	0.120	0.092	0.100	0.102	0.846
mean	0.888	0.388	0.576	0.763	0.763	0.716	0.946
STD	0.224	0.278	0.307	0.447	0.442	0.411	0.068
FFL	0.024	0.000	0.024	0.004	0.004	0.008	0.000
FFF	0.110	0.004	0.014	0.034	0.008	0.008	0.000
FSL	0.258	0.000	0.002	0.434	0.168	0.074	0.968
FST	0.044	0.000	0.000	0.088	0.018	0.006	0.947
FSF	0.084	0.000	0.000	0.000	0.000	0.000	0.977
FTL	0.326	0.064	0.088	0.286	0.172	0.122	0.607
FTT	0.408	0.014	0.040	0.346	0.106	0.084	0.306
FTF	0.000	0.000	0.000	0.000	0.000	0.000	0.000
FFT	0.354	0.120	0.172	0.276	0.226	0.180	0.394
ESL	0.816	0.000	0.000	0.478	0.000	0.000	0.936
EST	0.966	0.000	0.000	0.948	0.068	0.006	0.986
ESF	0.212	0.000	0.004	0.208	0.158	0.076	0.993
ETL	0.416	0.000	0.000	0.348	0.006	0.002	0.479
ETT	0.330	0.000	0.078	0.530	0.310	0.248	0.570
ETF	0.146	0.000	0.004	0.082	0.000	0.000	0.545
EFL	0.806	0.090	0.124	0.414	0.312	0.184	0.354
EFT	0.428	0.002	0.052	0.386	0.094	0.090	0.354
EFF	0.110	0.012	0.016	0.056	0.018	0.014	0.014
mean	0.357	0.019	0.036	0.305	0.104	0.068	0.589
STD	0.287	0.038	0.053	0.243	0.110	0.080	0.345
kappa	0.714	0.600	0.882	0.684	0.882	0.882	0.333
gap	0.532	0.369	0.540	0.458	0.659	0.648	0.357

Table S4. Classification results for 500 samples using 13 modes.
Table Description: (same as Supplementary Table 2)
Header rows colored in tan highlight the training set, where the (upper, lower) header rows define (functional, nonfunctional) synthetic molecules. The rows just below the (upper, lower) header list all remaining (4,16) unseen synthetic molecules that are (functional, nonfunctional). The trailing light blue rows mark the end of the section and provide the mean and standard deviation of the data within the section. The PCA, PLS and DR SPLOC methods of dimension reduction (DR) are compared across three sections of the table. Six classifiers applied to the trajectories of unseen molecules yield the likelihood that a molecule is functional. The discovery likelihood (DL) is also listed. The mean and standard deviations for likelihood over the set of functional and nonfunctional molecules are reported. The mean and standard deviations for likelihood over the set of functional and nonfunctional molecules are reported. The second to last row reports the Cohen kappa statistic(3) relative to the exact know result that all molecules of the form aLc are functional, and the remainder are not. The last row is the difference in the functional mean minus the nonfunctional mean, defining a gap. Gap values are color-coded for the standard classifiers: red when (gap < 0.3), yellow when (0.3 gap < 0.7), and green when (gap > 0.7), respectively indicating poor, good, and excellent predictions on average. The gap from DL is not color-coded because DL is not a classification likelihood. A (small, large) gap in DL gives (less, more) confidence to regard an unseen molecule as nonfunctional without an experimental test. An unseen molecule with characteristics that differ from known nonfunctional characteristics will have a high likelihood that an experimental test should be performed.

PCA		500 samples		13 modes			
	LDA	QDA	GNB	LSVM	QSVM	RBSVM	DL
FLL	1.000	1.000	0.994	1.000	1.000	1.000	0.943
FLF	1.000	1.000	0.960	1.000	1.000	1.000	0.942
FLT	1.000	0.986	1.000	1.000	1.000	1.000	0.983
ELL	0.956	0.812	0.618	0.848	0.910	0.972	0.926
ELT	0.990	0.644	0.808	0.874	0.660	0.672	0.936
ELF	0.334	0.028	0.386	0.008	0.380	0.032	0.950
mean	0.820	0.618	0.703	0.683	0.738	0.669	0.949
STD	0.325	0.417	0.263	0.455	0.278	0.450	0.025
FFL	0.004	0.000	0.002	0.000	0.000	0.000	0.810
FFF	0.026	0.000	0.000	0.018	0.000	0.000	0.812
FSL	0.492	0.522	0.594	0.482	0.516	0.650	0.873
FST	0.384	0.238	0.240	0.368	0.408	0.216	0.898
FSF	0.080	0.008	0.000	0.192	0.100	0.042	0.879
FTL	0.290	0.294	0.364	0.300	0.296	0.368	0.895
FTT	0.524	0.072	0.516	0.520	0.466	0.326	0.876
FTF	0.000	0.000	0.184	0.000	0.058	0.018	0.857
FFT	0.592	0.198	0.314	0.506	0.392	0.446	0.868
ESL	0.554	0.232	0.210	0.434	0.594	0.532	0.964
EST	0.922	0.538	0.710	0.836	0.834	0.788	0.953
ESF	0.698	0.726	0.626	0.662	0.804	0.488	0.938
ETL	0.674	0.208	0.490	0.544	0.670	0.552	0.916
ETT	0.810	0.460	0.676	0.758	0.616	0.626	0.892
ETF	0.060	0.300	0.260	0.180	0.766	0.198	0.912
EFL	0.262	0.010	0.068	0.108	0.226	0.098	0.944
EFT	0.762	0.350	0.504	0.656	0.714	0.650	0.920
EFF	0.242	0.154	0.428	0.290	0.642	0.250	0.886
mean	0.459	0.269	0.387	0.427	0.506	0.391	0.904
STD	0.283	0.210	0.216	0.239	0.243	0.237	0.032
kappa	0.280	0.600	0.391	0.333	0.231	0.391	0.000
gap	0.361	0.343	0.317	0.255	0.231	0.279	0.044

PLS		500 samples		13 modes			
	LDA	QDA	GNB	LSVM	QSVM	RBSVM	DL
FLL	1.000	1.000	1.000	1.000	1.000	1.000	0.978
FLF	1.000	1.000	0.786	1.000	1.000	1.000	0.932
FLT	1.000	1.000	1.000	1.000	1.000	0.900	0.964
ELL	0.982	0.930	0.978	1.000	1.000	0.972	0.958
ELT	0.838	0.568	1.000	1.000	0.696	0.666	0.975
ELF	0.590	0.344	0.838	0.176	0.968	0.178	0.912
mean	0.853	0.711	0.954	0.794	0.916	0.679	0.952
STD	0.189	0.309	0.078	0.412	0.147	0.359	0.028
FFL	0.000	0.000	0.360	0.000	0.000	0.000	0.838
FFF	0.002	0.000	0.000	0.018	0.000	0.000	0.812
FSL	0.234	0.276	0.840	0.470	0.950	0.628	0.975
FST	0.000	0.000	0.876	0.256	0.744	0.416	0.957
FSF	0.022	0.064	0.000	0.004	0.020	0.044	0.881
FTL	0.292	0.268	0.800	0.410	0.596	0.200	0.925
FTT	0.508	0.088	0.828	0.550	0.364	0.130	0.961
FTF	0.000	0.040	0.280	0.000	0.448	0.088	0.887
FFT	0.414	0.202	0.560	0.408	0.570	0.276	0.953
ESL	0.632	0.056	1.000	0.998	1.000	0.824	0.995
EST	0.812	0.226	1.000	1.000	1.000	0.992	0.956
ESF	0.144	0.216	0.838	0.700	1.000	0.704	0.989
ETL	0.258	0.034	1.000	0.874	0.976	0.624	0.993
ETT	0.418	0.012	1.000	0.972	0.722	0.568	0.929
ETF	0.262	0.118	0.898	0.422	1.000	0.316	0.899
EFL	0.652	0.052	0.992	0.832	0.822	0.162	0.986
EFT	0.244	0.126	1.000	0.920	0.932	0.674	0.961
EFF	0.250	0.176	0.808	0.710	0.986	0.414	0.937
mean	0.321	0.122	0.795	0.595	0.758	0.441	0.949
STD	0.239	0.093	0.286	0.333	0.290	0.285	0.037
kappa	0.636	0.882	0.125	0.231	0.161	0.333	0.000
gap	0.531	0.533	0.159	0.199	0.158	0.238	0.003

DR SPLOC		500 samples		13 modes			
	LDA	QDA	GNB	LSVM	QSVM	RBSVM	DL
FLL	1.000	1.000	1.000	1.000	1.000	1.000	0.977
FLF	1.000	1.000	1.000	1.000	1.000	1.000	0.992
FLT	1.000	0.666	0.798	1.000	1.000	1.000	0.884
ELL	1.000	0.666	0.750	1.000	1.000	1.000	0.918
ELT	0.998	0.444	0.782	1.000	1.000	0.996	0.948
ELF	0.372	0.000	0.004	0.114	0.028	0.014	0.713
mean	0.843	0.444	0.584	0.779	0.757	0.753	0.866
STD	0.314	0.314	0.387	0.443	0.486	0.492	0.105
FFL	0.000	0.000	0.006	0.000	0.000	0.000	0.000
FFF	0.022	0.002	0.012	0.018	0.002	0.002	0.000
FSL	0.190	0.000	0.000	0.480	0.486	0.210	0.847
FST	0.082	0.000	0.000	0.334	0.404	0.188	0.855
FSF	0.006	0.000	0.000	0.000	0.010	0.000	0.787
FTL	0.284	0.022	0.078	0.334	0.344	0.236	0.532
FTT	0.334	0.014	0.054	0.436	0.200	0.146	0.449
FTF	0.002	0.000	0.000	0.000	0.000	0.000	0.157
FFT	0.390	0.086	0.136	0.562	0.492	0.358	0.495
ESL	0.882	0.000	0.000	0.924	0.664	0.234	0.926
EST	0.916	0.000	0.000	0.994	0.924	0.682	0.942
ESF	0.266	0.000	0.002	0.344	0.362	0.250	0.915
ETL	0.260	0.000	0.000	0.516	0.146	0.064	0.632
ETT	0.392	0.002	0.066	0.762	0.670	0.440	0.596
ETF	0.014	0.000	0.000	0.086	0.010	0.004	0.626
EFL	0.682	0.010	0.082	0.668	0.626	0.366	0.481
EFT	0.354	0.000	0.066	0.754	0.148	0.110	0.480
EFF	0.116	0.000	0.006	0.148	0.062	0.014	0.149
mean	0.323	0.008	0.031	0.459	0.347	0.206	0.617
STD	0.286	0.022	0.043	0.310	0.282	0.188	0.251
kappa	0.600	0.750	0.882	0.333	0.524	0.778	0.286
gap	0.519	0.436	0.553	0.320	0.410	0.546	0.249

Table S5. Classification results for 20000 samples using 3 modes.
Table Description: (same as Supplementary Table 2)
Header rows colored in tan highlight the training set, where the (upper, lower) header rows define (functional, nonfunctional) synthetic molecules. The rows just below the (upper, lower) header list all remaining (4,16) unseen synthetic molecules that are (functional, nonfunctional). The trailing light blue rows mark the end of the section and provide the mean and standard deviation of the data within the section. The PCA, PLS and DR SPLOC methods of dimension reduction (DR) are compared across three sections of the table. Six classifiers applied to the trajectories of unseen molecules yield the likelihood that a molecule is functional. The discovery likelihood (DL) is also listed. The mean and standard deviations for likelihood over the set of functional and nonfunctional molecules are reported. The mean and standard deviations for likelihood over the set of functional and nonfunctional molecules are reported. The second to last row reports the Cohen kappa statistic(3) relative to the exact know result that all molecules of the form aLc are functional, and the remainder are not. The last row is the difference in the functional mean minus the nonfunctional mean, defining a gap. Gap values are color-coded for the standard classifiers: red when (gap < 0.3), yellow when (0.3 gap < 0.7), and green when (gap > 0.7), respectively indicating poor, good, and excellent predictions on average. The gap from DL is not color-coded because DL is not a classification likelihood. A (small, large) gap in DL gives (less, more) confidence to regard an unseen molecule as nonfunctional without an experimental test. An unseen molecule with characteristics that differ from known nonfunctional characteristics will have a high likelihood that an experimental test should be performed.

	PCA	20000 samples		3 modes			
	LDA	QDA	GNB	LSVM	QSVM	RBSVM	DL
FLL	0.488	0.492	0.500	0.715	0.768	0.898	1.000
FLF	0.633	0.721	0.642	0.518	0.634	0.961	1.000
FLT	0.315	0.366	0.331	0.639	0.630	0.478	1.000
ELL	0.721	0.740	0.794	0.938	0.999	0.568	1.000
ELT	0.705	0.736	0.758	0.966	0.993	0.508	1.000
ELF	0.950	0.973	0.969	0.922	0.458	0.624	1.000
mean	0.673	0.704	0.713	0.866	0.770	0.545	1.000
STD	0.264	0.251	0.271	0.153	0.270	0.065	0.000
FFL	0.360	0.364	0.369	0.590	0.660	0.099	1.000
FFF	0.442	0.524	0.451	0.431	0.625	0.051	1.000
FSL	0.300	0.383	0.291	0.699	0.575	0.482	1.000
FST	0.147	0.225	0.153	0.551	0.455	0.466	1.000
FSF	0.529	0.532	0.534	0.634	0.716	0.597	1.000
FTL	0.283	0.383	0.284	0.693	0.641	0.474	1.000
FTT	0.275	0.299	0.286	0.609	0.573	0.488	1.000
FTF	0.688	0.714	0.692	0.643	0.498	0.589	1.000
FFT	0.271	0.302	0.302	0.691	0.661	0.496	1.000
ESL	0.838	0.839	0.897	0.970	1.000	0.526	1.000
EST	0.648	0.591	0.720	0.936	0.987	0.530	1.000
ESF	0.907	0.936	0.932	0.901	0.484	0.633	1.000
ETL	0.572	0.767	0.662	0.973	0.995	0.547	1.000
ETT	0.409	0.660	0.510	0.970	0.984	0.547	1.000
ETF	0.939	0.962	0.958	0.857	0.437	0.592	1.000
EFL	0.602	0.776	0.678	0.969	0.997	0.534	1.000
EFT	0.465	0.702	0.541	0.980	0.973	0.536	1.000
EFF	0.788	0.839	0.815	0.878	0.625	0.615	1.000
mean	0.541	0.619	0.578	0.810	0.725	0.541	1.000
STD	0.249	0.239	0.258	0.157	0.224	0.052	0.000
kappa	0.120	0.000	0.037	0.029	0.032	0.143	0.000
gap	0.132	0.084	0.135	0.057	0.045	0.004	0.000

	PLS	20000 samples		3 modes			
	LDA	QDA	GNB	LSVM	QSVM	RBSVM	DL
FLL	0.717	0.783	0.575	0.850	0.689	0.917	0.999
FLF	0.828	0.869	0.805	0.601	0.711	0.946	0.999
FLT	0.588	0.658	0.364	0.931	0.669	0.499	0.999
ELL	0.815	0.874	0.843	0.972	0.788	0.752	1.000
ELT	0.640	0.741	0.751	0.956	0.794	0.615	1.000
ELF	0.878	0.912	0.977	0.608	0.699	0.728	1.000
mean	0.730	0.796	0.734	0.867	0.738	0.648	1.000
STD	0.138	0.118	0.264	0.173	0.063	0.116	0.001
FFL	0.243	0.296	0.388	0.661	0.429	0.109	0.999
FFF	0.361	0.408	0.569	0.353	0.538	0.110	0.999
FSL	0.613	0.695	0.342	0.947	0.660	0.511	0.999
FST	0.456	0.522	0.181	0.939	0.700	0.426	0.999
FSF	0.784	0.838	0.646	0.794	0.807	0.688	0.999
FTL	0.064	0.127	0.265	0.799	0.392	0.148	0.999
FTT	0.041	0.070	0.246	0.692	0.302	0.096	0.999
FTF	0.291	0.335	0.712	0.267	0.468	0.310	0.999
FFT	0.143	0.180	0.273	0.664	0.372	0.177	0.999
ESL	0.717	0.846	0.937	0.967	0.885	0.733	1.000
EST	0.623	0.734	0.807	0.958	0.859	0.650	1.000
ESF	0.912	0.940	0.974	0.578	0.760	0.758	1.000
ETL	0.140	0.229	0.529	0.846	0.612	0.214	1.000
ETT	0.079	0.130	0.319	0.851	0.471	0.116	1.000
ETF	0.393	0.439	0.951	0.205	0.650	0.450	1.000
EFL	0.257	0.321	0.559	0.793	0.527	0.285	1.000
EFT	0.207	0.264	0.383	0.779	0.480	0.225	0.999
EFF	0.452	0.496	0.854	0.493	0.616	0.476	1.000
mean	0.386	0.448	0.561	0.723	0.598	0.391	1.000
STD	0.278	0.287	0.282	0.233	0.177	0.228	0.001
kappa	0.565	0.500	0.185	0.125	0.241	0.455	0.000
gap	0.345	0.348	0.173	0.144	0.140	0.257	0.000

	DR SPLOC	20000 samples		3 modes			
	LDA	QDA	GNB	LSVM	QSVM	RBSVM	DL
FLL	0.867	0.966	0.966	0.989	0.949	0.963	1.000
FLF	0.893	0.971	0.971	0.994	0.957	0.973	1.000
FLT	0.887	0.970	0.970	0.993	0.956	0.970	1.000
ELL	0.848	0.965	0.965	0.988	0.944	0.958	1.000
ELT	0.863	0.957	0.957	0.989	0.934	0.953	0.999
ELF	0.838	0.957	0.957	0.982	0.928	0.950	1.000
mean	0.859	0.962	0.962	0.988	0.940	0.958	1.000
STD	0.021	0.006	0.006	0.004	0.012	0.009	0.001
FFL	0.305	0.195	0.195	0.466	0.150	0.151	0.000
FFF	0.317	0.190	0.190	0.457	0.143	0.147	0.000
FSL	0.030	0.005	0.005	0.033	0.000	0.001	0.999
FST	0.042	0.009	0.009	0.043	0.001	0.002	0.998
FSF	0.081	0.004	0.004	0.070	0.001	0.001	0.999
FTL	0.203	0.148	0.148	0.329	0.074	0.081	0.000
FTT	0.236	0.185	0.185	0.405	0.114	0.123	0.000
FTF	0.261	0.137	0.137	0.359	0.083	0.085	0.000
FFT	0.264	0.207	0.207	0.501	0.133	0.135	0.000
ESL	0.016	0.003	0.003	0.014	0.000	0.000	0.999
EST	0.043	0.003	0.003	0.036	0.000	0.000	0.999
ESF	0.061	0.005	0.005	0.050	0.000	0.001	0.999
ETL	0.129	0.111	0.111	0.253	0.054	0.061	0.000
ETT	0.200	0.147	0.147	0.313	0.083	0.090	0.000
ETF	0.208	0.126	0.126	0.253	0.068	0.074	0.000
EFL	0.233	0.188	0.188	0.365	0.129	0.135	0.000
EFT	0.213	0.171	0.171	0.356	0.109	0.115	0.000
EFF	0.294	0.189	0.189	0.394	0.141	0.147	0.000
mean	0.157	0.102	0.102	0.236	0.062	0.066	0.375
STD	0.097	0.082	0.082	0.166	0.055	0.057	0.499
kappa	1.000	1.000	1.000	0.895	1.000	1.000	0.500
gap	0.702	0.860	0.860	0.752	0.879	0.892	0.625

Table S6. Classification results for 20000 samples using 8 modes.
Table Description: (same as Supplementary Table 2)
Header rows colored in tan highlight the training set, where the (upper, lower) header rows define (functional, nonfunctional) synthetic molecules. The rows just below the (upper, lower) header list all remaining (4,16) unseen synthetic molecules that are (functional, nonfunctional). The trailing light blue rows mark the end of the section and provide the mean and standard deviation of the data within the section. The PCA, PLS and DR SPLOC methods of dimension reduction (DR) are compared across three sections of the table. Six classifiers applied to the trajectories of unseen molecules yield the likelihood that a molecule is functional. The discovery likelihood (DL) is also listed. The mean and standard deviations for likelihood over the set of functional and nonfunctional molecules are reported. The mean and standard deviations for likelihood over the set of functional and nonfunctional molecules are reported. The second to last row reports the Cohen kappa statistic(3) relative to the exact know result that all molecules of the form aLc are functional, and the remainder are not. The last row is the difference in the functional mean minus the nonfunctional mean, defining a gap. Gap values are color-coded for the standard classifiers: red when (gap < 0.3), yellow when (0.3 gap < 0.7), and green when (gap > 0.7), respectively indicating poor, good, and excellent predictions on average. The gap from DL is not color-coded because DL is not a classification likelihood. A (small, large) gap in DL gives (less, more) confidence to regard an unseen molecule as nonfunctional without an experimental test. An unseen molecule with characteristics that differ from known nonfunctional characteristics will have a high likelihood that an experimental test should be performed.

PCA	20000 samples				8 modes		
	LDA	QDA	GNB	LSVM	QSVM	RBSVM	DL
FLL	0.682	0.723	0.706	0.733	0.847	0.995	0.999
FLF	0.763	0.823	0.797	0.795	0.823	1.000	0.999
FLT	0.552	0.628	0.582	0.618	0.711	0.512	0.999
ELL	0.752	0.860	0.784	0.805	0.905	0.604	0.999
ELT	0.633	0.803	0.636	0.700	0.832	0.534	0.999
ELF	0.851	0.822	0.835	0.885	0.817	0.572	0.999
mean	0.697	0.778	0.709	0.752	0.816	0.555	0.999
STD	0.131	0.103	0.119	0.117	0.080	0.040	0.000
FFL	0.288	0.279	0.306	0.327	0.367	0.009	0.999
FFF	0.378	0.346	0.387	0.405	0.324	0.000	0.999
FSL	0.446	0.523	0.423	0.496	0.611	0.479	0.999
FST	0.393	0.586	0.417	0.465	0.643	0.442	0.999
FSF	0.668	0.705	0.705	0.706	0.737	0.525	0.999
FTL	0.142	0.202	0.185	0.174	0.209	0.242	0.999
FTT	0.128	0.186	0.145	0.161	0.234	0.189	0.999
FTF	0.377	0.435	0.399	0.401	0.381	0.367	0.999
FFT	0.190	0.253	0.212	0.222	0.281	0.198	0.999
ESL	0.683	0.863	0.715	0.749	0.896	0.554	0.999
EST	0.591	0.867	0.585	0.670	0.859	0.463	0.999
ESF	0.896	0.927	0.898	0.921	0.887	0.621	0.999
ETL	0.232	0.303	0.286	0.276	0.309	0.354	0.999
ETT	0.204	0.278	0.212	0.252	0.269	0.221	0.999
ETF	0.449	0.500	0.457	0.489	0.435	0.317	0.999
EFL	0.319	0.390	0.352	0.357	0.395	0.375	0.999
EFT	0.275	0.379	0.294	0.319	0.399	0.327	0.999
EFF	0.500	0.534	0.503	0.540	0.482	0.336	0.999
mean	0.406	0.496	0.424	0.450	0.502	0.376	0.999
STD	0.220	0.240	0.214	0.223	0.239	0.130	0.000
kappa	0.565	0.385	0.565	0.565	0.500	0.714	0.000
gap	0.291	0.283	0.285	0.302	0.315	0.180	0.000

PLS	20000 samples				8 modes		
	LDA	QDA	GNB	LSVM	QSVM	RBSVM	DL
FLL	0.953	0.928	0.500	0.949	0.982	0.998	1.000
FLF	0.967	0.943	0.806	0.960	0.886	1.000	1.000
FLT	0.960	0.924	0.321	0.961	0.973	0.599	1.000
ELL	0.992	0.976	0.722	0.989	0.989	0.726	1.000
ELT	0.987	0.962	0.698	0.981	0.984	0.663	1.000
ELF	0.969	0.950	0.961	0.963	0.915	0.628	1.000
mean	0.977	0.953	0.676	0.973	0.965	0.654	1.000
STD	0.015	0.022	0.264	0.014	0.034	0.055	0.000
FFL	0.205	0.161	0.334	0.196	0.224	0.007	1.000
FFF	0.230	0.173	0.582	0.216	0.157	0.001	0.999
FSL	0.029	0.016	0.296	0.027	0.070	0.455	0.999
FST	0.060	0.072	0.151	0.060	0.199	0.474	1.000
FSF	0.130	0.069	0.639	0.073	0.040	0.489	0.999
FTL	0.003	0.002	0.240	0.001	0.005	0.204	0.999
FTT	0.002	0.006	0.237	0.003	0.017	0.269	1.000
FTF	0.034	0.010	0.691	0.014	0.003	0.350	0.999
FFT	0.141	0.126	0.225	0.138	0.186	0.278	1.000
ESL	0.094	0.075	0.819	0.078	0.346	0.602	1.000
EST	0.069	0.083	0.579	0.066	0.403	0.598	1.000
ESF	0.190	0.122	0.935	0.113	0.123	0.621	1.000
ETL	0.011	0.003	0.470	0.005	0.019	0.256	1.000
ETT	0.003	0.002	0.299	0.001	0.014	0.266	1.000
ETF	0.055	0.020	0.912	0.020	0.016	0.362	1.000
EFL	0.221	0.180	0.505	0.203	0.243	0.342	1.000
EFT	0.207	0.173	0.381	0.196	0.250	0.364	1.000
EFF	0.337	0.262	0.829	0.294	0.273	0.402	1.000
mean	0.099	0.076	0.513	0.081	0.138	0.396	1.000
STD	0.098	0.078	0.266	0.087	0.134	0.132	0.000
kappa	1.000	1.000	0.120	1.000	1.000	0.714	0.000
gap	0.878	0.877	0.163	0.893	0.827	0.259	0.000

DR SPLOC	20000 samples				8 modes		
	LDA	QDA	GNB	LSVM	QSVM	RBSVM	DL
FLL	0.917	0.956	0.978	0.937	0.970	0.977	0.996
FLF	0.942	0.968	0.983	0.958	0.982	0.985	0.997
FLT	0.927	0.962	0.980	0.947	0.977	0.980	0.996
ELL	0.899	0.958	0.979	0.922	0.967	0.973	0.997
ELT	0.913	0.955	0.973	0.935	0.969	0.971	0.996
ELF	0.900	0.954	0.974	0.923	0.968	0.970	0.996
mean	0.910	0.957	0.976	0.932	0.970	0.973	0.996
STD	0.013	0.004	0.004	0.012	0.005	0.005	0.001
FFL	0.235	0.109	0.171	0.246	0.105	0.108	0.000
FFF	0.255	0.106	0.164	0.265	0.103	0.108	0.000
FSL	0.039	0.001	0.017	0.053	0.001	0.002	0.987
FST	0.036	0.001	0.023	0.043	0.001	0.004	0.989
FSF	0.104	0.001	0.013	0.119	0.001	0.002	0.991
FTL	0.054	0.010	0.109	0.046	0.015	0.024	0.000
FTT	0.066	0.013	0.123	0.065	0.024	0.029	0.000
FTF	0.086	0.012	0.096	0.076	0.021	0.029	0.000
FFT	0.143	0.083	0.165	0.162	0.087	0.097	0.000
ESL	0.027	0.000	0.010	0.038	0.000	0.001	0.992
EST	0.051	0.000	0.012	0.061	0.000	0.002	0.992
ESF	0.073	0.001	0.016	0.089	0.001	0.002	0.994
ETL	0.020	0.004	0.079	0.019	0.007	0.011	0.000
ETT	0.047	0.010	0.101	0.045	0.018	0.023	0.000
ETF	0.067	0.010	0.098	0.055	0.022	0.030	0.000
EFL	0.175	0.097	0.156	0.187	0.093	0.099	0.000
EFT	0.158	0.085	0.155	0.172	0.084	0.092	0.000
EFF	0.238	0.120	0.176	0.244	0.113	0.120	0.000
mean	0.086	0.028	0.084	0.092	0.030	0.035	0.372
STD	0.062	0.042	0.061	0.065	0.039	0.041	0.495
kappa	1.000	1.000	1.000	1.000	1.000	1.000	0.500
gap	0.823	0.929	0.892	0.840	0.940	0.938	0.625

Table S7. Classification results for 20000 samples using 13 modes.
Table Description: (same as Supplementary Table 2)
Header rows colored in tan highlight the training set, where the (upper, lower) header rows define (functional, nonfunctional) synthetic molecules. The rows just below the (upper, lower) header list all remaining (4,16) unseen synthetic molecules that are (functional, nonfunctional). The trailing light blue rows mark the end of the section and provide the mean and standard deviation of the data within the section. The PCA, PLS and DR SPLOC methods of dimension reduction (DR) are compared across three sections of the table. Six classifiers applied to the trajectories of unseen molecules yield the likelihood that a molecule is functional. The discovery likelihood (DL) is also listed. The mean and standard deviations for likelihood over the set of functional and nonfunctional molecules are reported. The mean and standard deviations for likelihood over the set of functional and nonfunctional molecules are reported. The second to last row reports the Cohen kappa statistic(3) relative to the exact know result that all molecules of the form aLc are functional, and the remainder are not. The last row is the difference in the functional mean minus the nonfunctional mean, defining a gap. Gap values are color-coded for the standard classifiers: red when (gap < 0.3), yellow when (0.3 gap < 0.7), and green when (gap > 0.7), respectively indicating poor, good, and excellent predictions on average. The gap from DL is not color-coded because DL is not a classification likelihood. A (small, large) gap in DL gives (less, more) confidence to regard an unseen molecule as nonfunctional without an experimental test. An unseen molecule with characteristics that differ from known nonfunctional characteristics will have a high likelihood that an experimental test should be performed.

	PCA	20000 samples			13 modes		
	LDA	QDA	GNB	LSVM	QSVM	RBSVM	DL
FLL	0.800	0.834	0.813	0.814	0.869	1.000	0.999
FLF	0.819	0.842	0.827	0.821	0.877	1.000	0.999
FLT	0.752	0.728	0.770	0.760	0.725	0.663	0.999
ELL	0.867	0.893	0.876	0.871	0.910	0.772	0.999
ELT	0.820	0.866	0.795	0.811	0.880	0.819	0.999
ELF	0.822	0.731	0.821	0.823	0.766	0.701	0.999
mean	0.815	0.805	0.815	0.816	0.820	0.739	0.999
STD	0.048	0.087	0.046	0.046	0.089	0.070	0.000
FFL	0.250	0.223	0.261	0.256	0.243	0.001	0.999
FFF	0.276	0.209	0.257	0.276	0.195	0.000	0.999
FSL	0.507	0.566	0.445	0.486	0.590	0.566	0.999
FST	0.451	0.595	0.526	0.442	0.656	0.658	0.999
FSF	0.463	0.413	0.512	0.436	0.490	0.438	0.999
FTL	0.077	0.102	0.106	0.072	0.098	0.191	0.999
FTT	0.070	0.112	0.091	0.067	0.123	0.183	0.999
FTF	0.129	0.168	0.178	0.112	0.139	0.193	0.999
FFT	0.171	0.181	0.206	0.172	0.180	0.210	0.999
ESL	0.785	0.862	0.826	0.771	0.893	0.853	0.999
EST	0.584	0.797	0.602	0.580	0.867	0.798	0.999
ESF	0.663	0.614	0.698	0.647	0.697	0.575	0.999
ETL	0.080	0.118	0.133	0.074	0.138	0.266	0.999
ETT	0.038	0.069	0.070	0.035	0.079	0.183	0.999
ETF	0.180	0.204	0.217	0.159	0.215	0.292	0.999
EFL	0.274	0.298	0.298	0.268	0.298	0.316	0.999
EFT	0.233	0.289	0.253	0.232	0.304	0.346	0.999
EFF	0.392	0.362	0.404	0.376	0.361	0.364	0.999
mean	0.319	0.359	0.348	0.308	0.383	0.402	0.999
STD	0.235	0.256	0.233	0.231	0.279	0.223	0.000
kappa	0.636	0.565	0.565	0.714	0.565	0.565	0.000
gap	0.497	0.445	0.468	0.508	0.437	0.337	0.000

	PLS	20000 samples			13 modes		
	LDA	QDA	GNB	LSVM	QSVM	RBSVM	DL
FLL	0.980	0.952	0.442	0.982	0.979	0.999	1.000
FLF	0.987	0.956	0.816	0.987	0.966	1.000	1.000
FLT	0.984	0.921	0.310	0.991	0.975	0.623	1.000
ELL	0.994	0.966	0.624	0.991	0.961	0.729	1.000
ELT	0.996	0.978	0.593	0.998	0.978	0.630	1.000
ELF	0.982	0.921	0.954	0.980	0.902	0.539	1.000
mean	0.989	0.946	0.620	0.990	0.954	0.630	1.000
STD	0.007	0.030	0.264	0.007	0.035	0.078	0.000
FFL	0.194	0.123	0.291	0.175	0.163	0.004	1.000
FFF	0.207	0.114	0.598	0.187	0.128	0.000	1.000
FSL	0.002	0.000	0.267	0.000	0.000	0.429	1.000
FST	0.001	0.000	0.138	0.000	0.031	0.447	1.000
FSF	0.033	0.000	0.663	0.001	0.022	0.474	1.000
FTL	0.013	0.001	0.208	0.002	0.002	0.229	1.000
FTT	0.005	0.000	0.183	0.002	0.015	0.242	1.000
FTF	0.034	0.001	0.698	0.004	0.010	0.374	1.000
FFT	0.135	0.097	0.220	0.126	0.155	0.259	1.000
ESL	0.001	0.000	0.662	0.000	0.014	0.571	1.000
EST	0.000	0.000	0.485	0.000	0.049	0.516	1.000
ESF	0.046	0.001	0.958	0.001	0.090	0.580	1.000
ETL	0.008	0.001	0.378	0.002	0.005	0.437	1.000
ETT	0.006	0.000	0.203	0.001	0.009	0.368	1.000
ETF	0.038	0.001	0.913	0.003	0.031	0.389	1.000
EFL	0.176	0.125	0.462	0.152	0.160	0.435	1.000
EFT	0.164	0.119	0.339	0.150	0.178	0.411	1.000
EFF	0.266	0.174	0.798	0.221	0.223	0.395	1.000
mean	0.058	0.033	0.473	0.042	0.062	0.410	1.000
STD	0.081	0.059	0.274	0.074	0.074	0.103	0.000
kappa	1.000	1.000	0.217	1.000	1.000	0.714	0.000
gap	0.931	0.914	0.147	0.949	0.892	0.221	0.000

	DR SPLOC	20000 samples			13 modes		
	LDA	QDA	GNB	LSVM	QSVM	RBSVM	DL
FLL	0.993	0.959	0.985	0.986	0.988	0.995	0.967
FLF	0.994	0.967	0.987	0.988	0.990	0.995	0.972
FLT	0.996	0.963	0.985	0.991	0.988	0.992	0.971
ELL	0.995	0.964	0.983	0.986	0.989	0.992	0.979
ELT	0.993	0.952	0.974	0.980	0.987	0.990	0.975
ELF	0.991	0.954	0.975	0.975	0.985	0.987	0.976
mean	0.994	0.958	0.979	0.983	0.987	0.990	0.975
STD	0.002	0.006	0.005	0.007	0.002	0.002	0.003
FFL	0.196	0.084	0.121	0.170	0.068	0.062	0.000
FFF	0.198	0.085	0.115	0.176	0.069	0.063	0.000
FSL	0.000	0.000	0.000	0.000	0.000	0.000	0.881
FST	0.001	0.000	0.000	0.000	0.000	0.000	0.855
FSF	0.001	0.000	0.000	0.000	0.000	0.000	0.883
FTL	0.010	0.000	0.000	0.002	0.000	0.000	0.000
FTT	0.003	0.000	0.000	0.001	0.000	0.000	0.000
FTF	0.012	0.000	0.000	0.003	0.000	0.000	0.000
FFT	0.118	0.058	0.085	0.104	0.051	0.048	0.000
ESL	0.000	0.000	0.000	0.000	0.000	0.000	0.920
EST	0.001	0.000	0.000	0.000	0.000	0.000	0.896
ESF	0.000	0.000	0.000	0.000	0.000	0.000	0.907
ETL	0.006	0.000	0.000	0.001	0.000	0.000	0.000
ETT	0.008	0.000	0.000	0.003	0.000	0.000	0.000
ETF	0.014	0.000	0.000	0.003	0.000	0.000	0.000
EFL	0.169	0.079	0.104	0.145	0.066	0.062	0.000
EFT	0.138	0.061	0.092	0.116	0.055	0.049	0.000
EFF	0.199	0.097	0.128	0.174	0.080	0.073	0.000
mean	0.043	0.018	0.026	0.034	0.016	0.014	0.334
STD	0.070	0.034	0.047	0.061	0.029	0.026	0.445
kappa	1.000	1.000	1.000	1.000	1.000	1.000	0.500
gap	0.951	0.940	0.954	0.949	0.972	0.976	0.641

References

1. Arthur Cayley. Sur quelques propriétés des déterminants gauches. *Journal für die reine und angewandte Mathematik*, 32: 119–123, 1846.
2. Jenny Farmer and Donald Jacobs. High throughput nonparametric probability density estimation. *PloS one*, 13(5), 2018.
3. Jacob Cohen. A coefficient of agreement for nominal scales. *Educational and psychological measurement*, 20(1):37–46, 1960.
